# High quality chromosomal genome assemblies of three human Plasmodium species directly from natural infections

**DOI:** 10.1371/journal.ppat.1014754

**Published:** 2026-10-30

**Authors:** Sunil Kumar Dogga, Jesse C. Rop, Alex Makunin, Fiona Teltscher, Damon-Lee Pointon, Ying Sims, Marcela Uliano-Silva, James Torrance, Thomas C. Mathers, Jonathan M. D. Wood, Sekou Sissoko, Antoine Dara, Dinkorma T. Ouologuem, Arthur M. Talman, Abdoulaye A. Djimdé, Mara K. N. Lawniczak

**Affiliations:** 1 Tree of Life, Wellcome Sanger Institute, Hinxton, United Kingdom; 2 Swiss Tropical and Public Health Institute, Kreuzstrasse 2, Allschwil, Switzerland; 3 Malaria Research and Training Center (MRTC), Faculty of Pharmacy, Université des Sciences, des Techniques et des Technologies de Bamako (USTTB), Bamako, Mali; 4 MIVEGEC, University of Montpellier, IRD, CNRS, Montpellier, France; Oregon State University, UNITED STATES OF AMERICA

## Abstract

Malaria, caused by mosquito-transmitted *Plasmodium* parasites, remains a major global health challenge. While *P. falciparum* accounts for most malaria deaths, the lesser-studied *P. ovale* spp. and *P. malariae* also contribute to disease burden across many endemic regions, typically causing milder but chronic infections. Here, we generated high quality, chromosome-level reference genomes for *P. ovale wallikeri*, *P. malariae,* and *P. falciparum* directly from blood samples taken from natural infections. Using PacBio Ultra-Low-Input HiFi long-read sequencing and Hi-C chromatin conformation capture, we overcome limitations posed by low parasite biomass and the absence of *in vitro* culture for *P. ovale wallikeri* and *P. malariae.* These high quality reference genomes resolve previously inaccessible subtelomeric regions that include expanded gene families likely involved in host–parasite interactions, and they enhance our understanding of parasite persistence, transmission, immune evasion and comparative evolutionary studies. This work provides a critical foundation for including these neglected species in global malaria elimination efforts.

## Author summary

Our study presents high-quality, chromosome-level reference genomes for *Plasmodium ovale*, *Plasmodium malariae* and *Plasmodium falciparum* sampled directly from natural infections. While *P. falciparum* has been extensively studied due to its major role in severe malaria and global health burden, *P. ovale* spp. and *P. malariae* remain comparatively neglected with limited genomic resources, despite their wide distribution, chronic infections, and diagnostic challenges. Using PacBio Ultra Low Input HiFi long-read sequencing directly on enriched parasites from clinical samples collected in Mali, we overcome the challenges of low parasite biomass and the lack of in vitro culture systems for these neglected species. The resulting assemblies resolve previously inaccessible subtelomeric regions and reveal new insights into gene families implicated in host–parasite interactions. Alongside comprehensive gene annotations and structural insights, these resources lay the groundwork for better diagnostics, therapeutic target discovery, and comparative evolutionary studies of malaria parasites. This work provides tools to expand our understanding of *P. ovale* and *P. malariae* biology and provides an essential foundation for integrating these species into broader malaria research and elimination strategies.

## Introduction

Malaria remains a major global health issue, with half of the world’s population thought to be at risk (WHO). In humans, the disease is caused by *Plasmodium falciparum*, *P. vivax*, *P. knowlesi*, *P. ovale*, and *P. malariae* species. While *P. falciparum* continues to be the primary cause of morbidity and mortality in sub-Saharan Africa (SSA), non-*falciparum* species such as *P. ovale* and *P. malariae* are increasingly recognized as contributors to the malaria burden. These species were often overlooked due to misdiagnosis, limited molecular surveillance, and a lack of dedicated research focus [1,2]. *P. ovale* is also understood to be two genetically distinct but morphologically identical species, *P. ovale curtisi* and *P. ovale wallikeri,* which are sympatric, non-recombining, and differ subtly in latency periods [3,4]. Across West Africa, where both *P. ovale* species circulate, the relative contribution of each remains poorly characterized due to the absence of accurate diagnostic markers. These parasites are believed to form hypnozoites in the liver, similar to *P. vivax*, allowing them to cause relapsing infections long after the initial exposure [5], although the evidence for this is minimal [6]. *P. malariae*, on the other hand, is capable of establishing chronic blood infections that may persist asymptomatically for years or even decades [7]. Although traditionally considered to cause relatively mild illness, *P. ovale* and *P. malariae* have both been associated with serious complications, including severe anemia, splenomegaly, and in rare cases, congenital malaria [8–12]. *P. malariae* has also been specifically implicated in nephrotic syndrome [13,14]. Morbidity associated with long-term carriage of these malaria species is understudied and these infections are likely underreported, in part due to diagnostic limitations and frequent co-infections with *P. falciparum*, which obscures clinical attribution. Recent reports suggest that over half of *P. ovale* and *P. malariae* infections in SSA occur as mixed infections with *P. falciparum* [1,2,15].

The prevalence of *P. ovale* spp. and *P. malariae* has been shown to be stable even in areas where *P. falciparum* incidence is diminishing [16–18], marking these species as a distinct challenge in eradication efforts. Despite this growing epidemiological relevance, the biology and genetics of *P. ovale* spp. and *P. malariae* remain insufficiently studied due to the absence of continuous *in vitro* culture systems and challenges in accessing clinical samples due to the their typical occurrence as mixed infections and the difficulty of accurate species diagnosis in field settings.

From a global health perspective, the neglect of these parasites poses several risks. They were increasingly identified in travel-related malaria cases, often evading standard prophylaxis, which highlights their potential for silent spread and reintroduction into previously malaria-free regions [19–21]. Moreover, reports of resistance-conferring mutations, such as those in the *dhfr* gene, raise concerns about the future efficacy of current antimalarial regimens [22,23], especially when these parasites are often co-exposed to *P. falciparum* therapeutic regimen which may be suboptimal and provide good resistance-selecting conditions.

The molecular toolkits available to study these species have only recently begun to expand. For *P. falciparum*, the 3D7 reference genome was first assembled over two decades ago [24] and later improved using PacBio long-read sequencing [25]. The nearly telomere-to-telomere representation of all 14 chromosomes resolves most centromeric and subtelomeric regions and remains a highly curated, gold-standard reference, albeit from a laboratory-adapted strain. On the other hand, for *P. ovale* spp. and *P. malariae*, the first reference assemblies (PocGH01, PowCR01, and PmUG01) were published in 2017 [26]. The *P. malariae* PmUG01 assembly was generated using PacBio long reads and iterative polishing, markedly improving contiguity (61 scaffolds with 14 gapless chromosome contigs, scaffold N50 ≈ 2.3 Mb), whereas the *P. ovale curtisi* and *wallikeri* assemblies in this study were derived largely from short-read Illumina data. Subsequent updates have incorporated long-read sequencing and selective whole-genome amplification (sWGA) to enhance contiguity and gene annotation, particularly in subtelomeric multigene families [27–29]. The recent *P. ovale curtisi* and *wallikeri* reference genomes (Poc221 and Pow222) generated from clinical samples obtained from travellers returning to the UK, were produced using a hybrid approach that sequenced both sWGA-enriched and non-enriched material with Oxford Nanopore Technology (ONT) MinION under adaptive sampling conditions to deplete human DNA [28]. The ONT reads had mean lengths of 1.15 Kbp and 1.17 Kbp for Poc221 and Pow222, respectively. The resulting assemblies (scaffold N50s of 217 Kb for Poc221 and 270 Kb for Pow222) represent improvements in contiguity and gene annotation, particularly across subtelomeric multigene families such as *pir*, *phist*, and *resa*-like genes. However, sWGA introduces uneven coverage across the genome [30], resulting in fragmented assemblies and incomplete or inaccurate gene annotations, evidenced by the high number of scaffolds (1177 and 787) and the large fraction of genome present as unlocalised contigs (~35%). Also, subtelomeric multigene families were often poorly covered by sWGA primer sets and may therefore be underrepresented in sWGA-derived data. Moreover, the absence of Hi-C or other chromatin-conformation data for *P. ovale* spp. and *P. malariae* has prevented chromosome-scale scaffolding, leaving numerous contigs unlocalised and not associated with chromosomes and structural breaks across all published references. Collectively, these factors continue to limit comprehensive analyses of genome architecture, synteny, and subtelomeric gene family diversity. Overall, the best reference genomes currently available are the sWGA-enriched ONT assemblies Poc221 and Pow222 for *P. ovale curtisi* and *P. ovale wallikeri*, respectively, and the PacBio long-read based *P. malariae* PmUG01.

Here, we present high quality, chromosome-level genome assemblies of *P. ovale wallikeri*, *P. malariae*, and *P. falciparum*, generated from clinical infections in Faladie, Mali, a hyperendemic region characterized by intense seasonal malaria transmission. After the elimination of leukocytes and uninfected RBCs, we obtained enriched parasite material from clinical infections, generating long read PacBio data from as little as 2 ng of DNA without culture adaptation or sWGA. Using the Ultra-Low Input (ULI) PacBio long-read sequencing kit and Hi-C, we reconstructed near-complete genomes with greatly improved contiguity and annotation, particularly in subtelomeric regions enriched for multigene families. These assemblies provide an essential foundation for understanding the biology, gene function, and evolutionary relationships of these neglected malaria parasites.

## Results

### Processing, sequencing, and curation of genome assemblies from natural malaria infections

To generate high quality genome references, we enriched parasite fractions from the blood samples of eighteen study participants in Faladie (Mali), representing both symptomatic and asymptomatic malaria cases with varying mixed species infection status. Eight of these samples yielded sufficient parasite material to generate high-quality reference genome assemblies (Table 1, S1A Fig). Intravenous whole blood was processed without further culturing using a previously published protocol that enriches parasites using Magnetic-activated cell sorting (MACS) and Streptolysin-O (SLO) (S1A Fig) [31]. High Molecular Weight DNA was then sequenced with PacBio’s Ultra Low Input High-Fidelity (ULI-HiFi) long-read sequencing protocol. Mapping HiFi reads to the *P. ovale* spp. reference genomes (PocGH01, https://plasmodb.org/: v68 and Pow222 [28],), and examining the *cox1* locus confirmed all *P. ovale* spp. isolates as *P. ovale wallikeri* (S1B Fig).

**Table 1:** Assembly summary for all the specimens, in comparison to current published references.

	Donor ID	ID (Source)	tolID	Full assembly					Main Chromosomes					chrM(bp)	chrA(bp)
				n	Size(Mbp)	Gain(Mbp)	GC%	Gaps	Size(Mbp)	Gain(Mbp)	GC%	Gaps	Chr Ends		
***P. ovale wallikeri, Pow***	*MSC23	PowML03	pxPlaOval3	15	38.05	3.66	28.37	36	37.83	12.01	28.42	9	3 (14)	5975	34363
	MSC32	PowML04	pxPlaOval4	17	34.26	-0.13	28.85	32	32.03	6.21	29.38	32	4 (11)	5980	29156
	MSC31	PowML08	pxPlaOval8	83	34.9	0.51	28.99	166	29.53	3.71	30.38	165	0 (13)	----	32892
	MSC25	PowML09	pxPlaOval9	24	37.99	3.6	28.36	58	34.95	9.13	28.99	58	2 (14)	5970	33620
	-----	Pow222 (Nigeria)	-----	787	34.39	---	29.13	182	25.82	---	31.58	182	---	5975	34310
	-----	PowCR01 (Ghana)	-----	779	33.53	-0.86	29.26	1255	20.92	-4.9	33.14	1167	---	5584	31160
***P. malariae, Pm***	*MSC29	PmML03	pxPlaMala3	14	36.78	3.2	23.93	13	36.78	7.24	23.93	9	7 (13)	5966	35642
	MSC19	PmML04	pxPlaMala4	17	35.2	1.62	24.16	18	34.94	5.4	24.2	18	4 (13)	5967	33199
	MSC28	PmML07	pxPlaMala7	75	26.08	-7.5	25.05	422	22.64	-6.9	25.98	399	0 (8)	----	----
	-----	PmUG01(Uganda)	-----	61	33.58	---	24.39	0	29.54	---	25.12	0	---	5969	34324
*P. Brasilianum(Bolivian I)*			-----	43	31.37	---	24.81	8	29.08	---	25.16	7	---	6302	28948
***P. falciparum, Pf***	MSC28	PfML46	pxPlaFalc46	34	23.88	0.59	19.61	24	23.21	-0.08	19.32	23	6 (14)	5967	32080
	MSC61	PfML47	pxPlaFalc47	16	23.37	0.08	19.4	7	23.3	0.01	19.37	7	8 (14)	5966	34244
	MSC29	PfML72	pxPlaFalc72	14	23.21	-0.08	19.35	31	23.21	-0.08	19.35	31	9 (14)	----	32282
	-----	Pf3D7	-----	14	23.29	---	19.34	0	23.29	---	19.34	0	13 (14)	5967	34250

The highest-quality assemblies for each species are indicated in green. Specimens used for generating Hi-C data are indicated with a * next to the participant ID, and colored boxes are used to indicate those with mixed infections.”Mbp” - Megabase pair,”chrM” - mitochondrial chromosome,”chrA” - apicoplast chromosome, “Chr Ends” - number of chromosomes with telomeric repeat sequences on both ends, in parentheses noting down the number of chromosomes with both ends harboring a region of subtelomeric gene families.

Two Arima Hi-C libraries were generated and sequenced on Illumina platforms, one from a participant with a *P. ovale wallikeri* infection (MSC23) and one from a participant with a mixed *P. malariae*/*P. falciparum* infection (MSC29). These datasets were used to scaffold PacBio ULI contigs (Fig 1, S1A, S1C–S1E Fig, Methods). The ULI approach enabled high-quality genome assemblies from specimens with low DNA quantities for *P. ovale wallikeri* (MSC23, 25, 31, 32), *P. malariae* (MSC 19, 28, 29), and *P. falciparum* (MSC28, 29, 61) (S1A Fig). PacBio HiFi reads were depleted of host sequences by mapping to the GRCh38 (GENCODE v32/Ensembl 98) human reference genome, retaining only unmapped reads for assembly with hifiasm (v0.16) [32] (S1 Table) with the *-l 0* option to disable purging. MSC28 and MSC29 were from *P. falciparum*/*P. malariae* mixed species infections, enabling recovery of assemblies for both parasite species, resulting in ten *Plasmodium* genomes from the eight participants.

**Fig 1:**
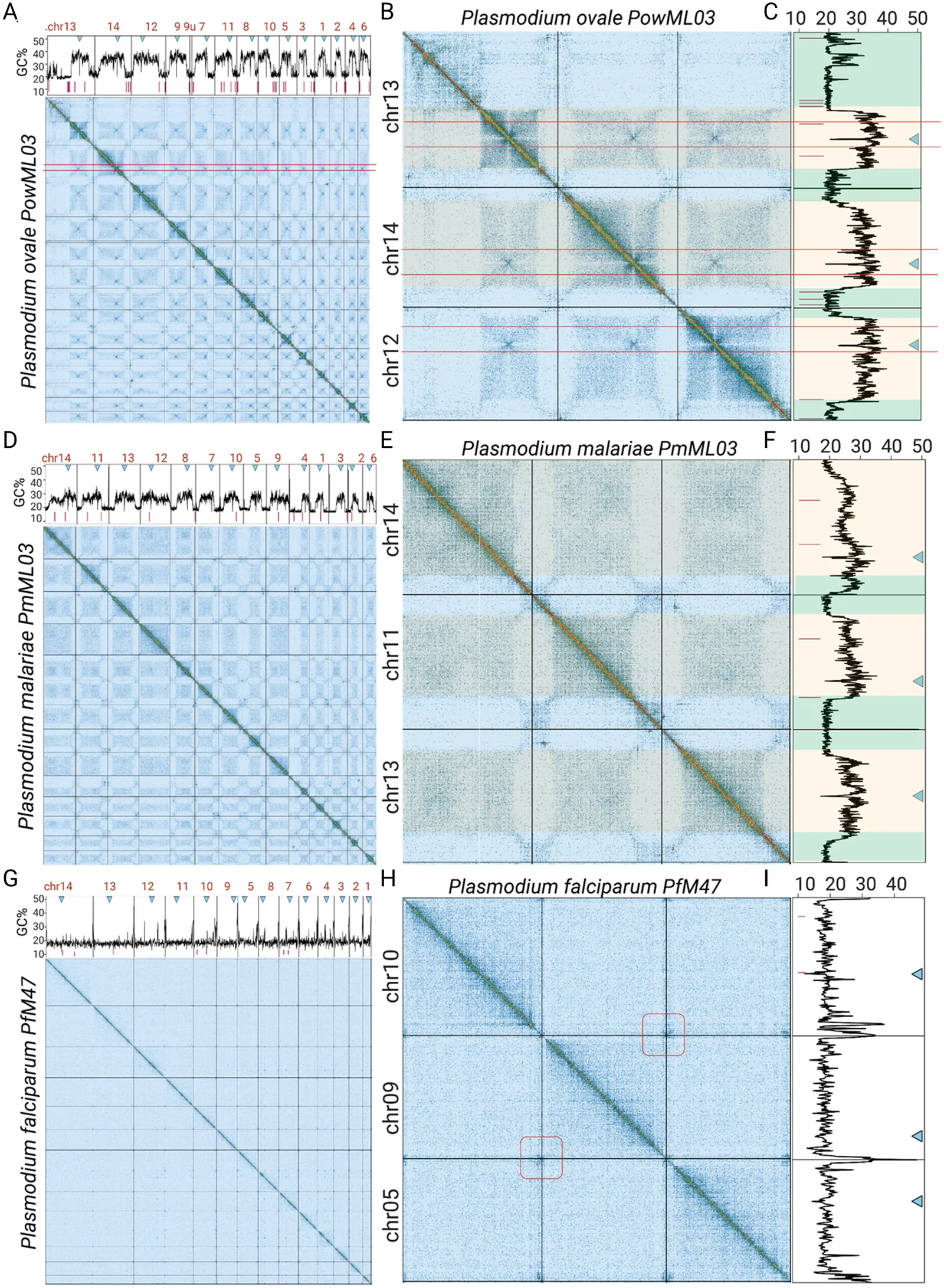
Hi-C and PacBio HiFi enable chromosomal assemblies for *P. ovale wallikeri*, *P. malariae,* and *P. falciparum*, and reveal distinct chromosomal architecture and centromere clustering patterns. **A,D,G,** Pretext file snapshot of Hi-C contact maps of super scaffolds (chromosomes) of **(A)** PowML03, **(D)** PmML03, and **(G)** PfML47, arranged by size. GC content was calculated in 10,000 bp non-overlapping bins and plotted along each chromosome, with vertical black lines indicating chromosome boundaries. Maroon colored lines along the plot in (**A**) illustrate the cross-like patterns in putative centromeres driven by interchromosomal interactions and centromere clustering. Chromosome ID is displayed on top of each panel. **B,E,H**, A zoomed in snapshot of three chromosomes further illustrates the features of the Hi-C contact maps. **C,F,I**, The GC content profile mirrors chromosome-scale patterns observed in the Hi-C contact maps, with a high-GC core in central regions of chromosomes (yellow) and reduced GC content in the subtelomeric regions (green) for PowML03 and PmML03. Centromere coordinates, listed in S2 Table, of PowML03 and PmML03 were determined using the cross-shaped interaction patterns typically associated with centromeric domains, indicated by blue inverted triangles. GC content was used to infer centromeric regions in *P. falciparum*, by selecting loci where GC content dropped below 5% across two consecutive 1000 bp bins. While interchromosomal interactions are not apparent in PfML47, indications of interactions at the subtelomeric regions of PfML47 are highlighted with maroon boxes in **(H).** Red segments under the GC lineplot in corresponding to breaks in the assembly.

Heterozygosity estimates revealed a minor allele fraction below 0.10 across all samples, except for pxPlaOval8 (0.18, assembly PowML08) and *P. malariae* fraction of the mixed infection sample pxPlaFalc46 (0.37, assembly PmML07) (S1 Table). The estimates for the latter two samples should be interpreted with caution due to their low sequencing depth; notably, assemblies PowML08 and PmML07 are also among the lowest-quality assemblies in the dataset, exhibiting the most gaps and unplaced contigs. Purging normally removes divergent haplotig sequences that the assembler interprets as low frequency noise or ordinary heterozygosity. However, even with purging disabled, if a clone is present at low relative abundance, the assembler can still interpret it as background error and exclude it from the primary assembly graph. In this case, the primary contig output that is used for scaffolding, reflects only the majority clone’s sequence. If the minority clone’s sequence is distinct enough to survive, the assembler’s internal filtering will report its sequences in the alternate contig file. If the clones are divergent, hifiasm can fail to recognize the two divergent sequences as alternate haplotypes and instead places both into the primary contig file, like they are two unrelated regions of the genome, rather than considering one as an alternate haplotype. We did not observe evidence of alternate haplotypes that remained in the primary contig file. These will be apparent as duplications in the dotplots and in Hi-C contact maps during scaffolding, as discussed below (S2A Fig).

Genome contiguity was assessed via N50 statistics and comparison with existing references (Fig 2, S2A Fig, S1 Table, Table 1). Initial hifiasm assemblies ranged from 51-605 contigs, with contig N50 values from 149,862 bp to 1,839,254 bp, the latter being nearly two-thirds the size of the largest chromosome of *P. falciparum* 3D7 (3.29 Mbp) and larger than the theoretical maximum N50 for *P. falciparum* 3D7*,* which is ≈ 1.688 Mb, corresponding to chromosome 10, indicating high assembly contiguity (S1 Table). Hi-C data from MSC23 (a *P. ovale wallikeri* infection) was used to scaffold all *P. ovale wallikeri* assemblies, and that from MSC29 (participant with *P. falciparum/P. malariae* mixed infection) was used to scaffold all *P. malariae* and *P. falciparum* assemblies with the tool YaHS [33], followed by manual curation. This approach enabled the scaffolding of the hifiasm contigs from the *P. ovale wallikeri* assembly (PowML03), *P. malariae* assembly (PmML03) and *P. falciparum* assembly (PfML47) into the 14 chromosomes of the final assemblies, which together represented the highest-quality assemblies for the three species, with improvements in genome size, contiguity, and chromosomal completeness (Fig 2). A single contig of PowML03, which likely belongs to chr09 based on Hi-C contact maps, remained unplaced and labelled chr09u. PowML03 and PmML03 were each scaffolded using Hi-C data derived from their respective specimens, while PfML47 was scaffolded using Hi-C data from another participant, MSC29 (which yielded the PmML03 and PfML72 assemblies) (S1A Fig). Mitochondrial and apicoplast genomes (chrM, chrA) were identified from the contigs using MitoHiFi (Table 1) [34].

**Fig 2:**
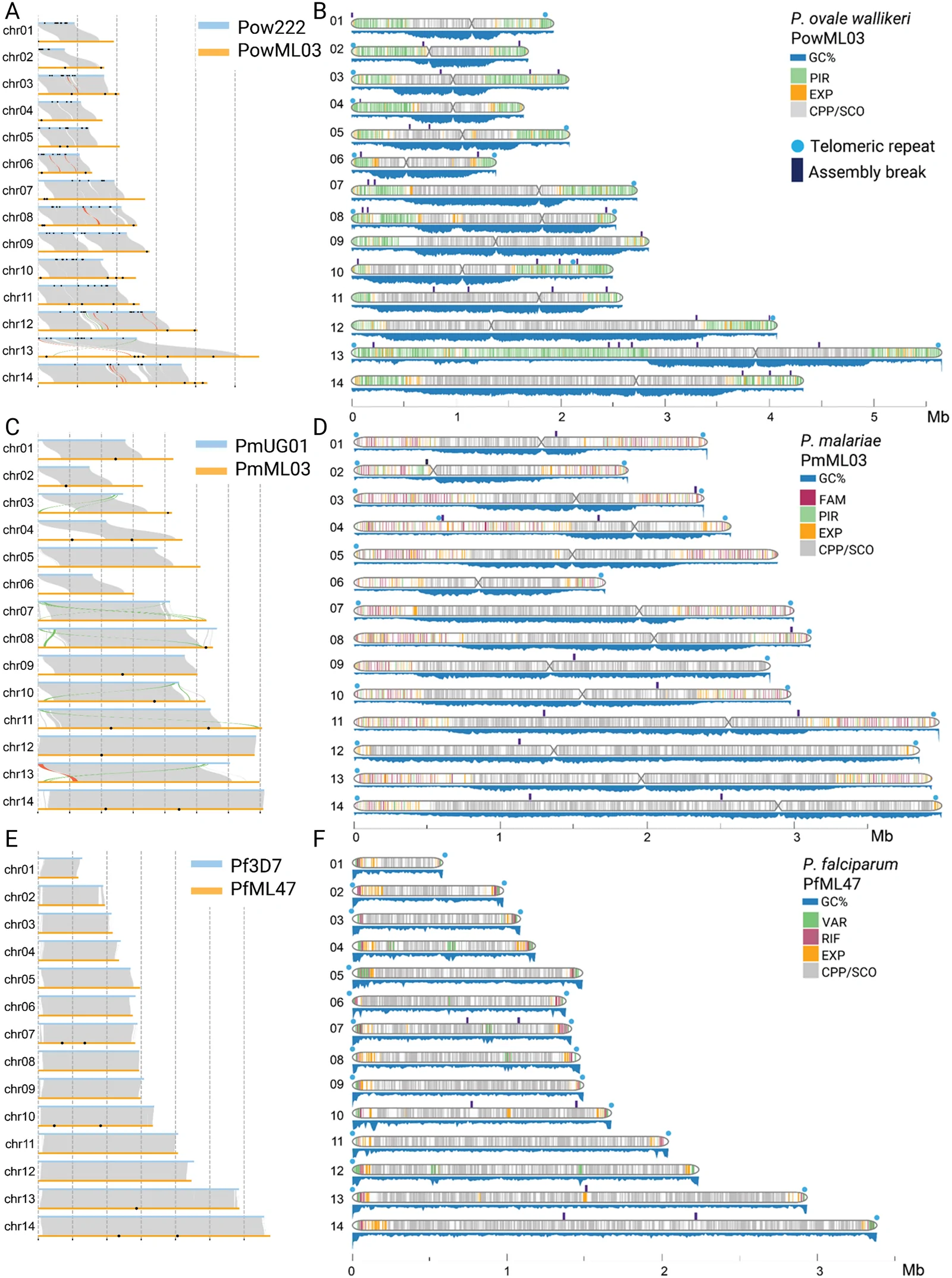
Comprehensive annotation of refined reference assemblies reveal marked localisation of conserved genes and multigene families. **A,C,E**, SyRI synteny plot of each species’ best assembly for **(A)** *P. ovale wallikeri* (PowML03), **(C)** *P. malariae* (PmML03) and **(E)** *P. falciparum* (PfML47) in comparison to the existing best references, Pow222, PmUG01 and Pf3D7, respectively. **B,D,F,** Chromosome plots for (**B)** *P. ovale wallikeri* (PowML03), **(D)** *P. malariae* (PmML03), and **(F)** *P. falciparum* (PfML47) showing the GC content (blue) in 10 kbp bins scaled from a minimum of 15%, presence of genes coding for multigene families (in colour) and single copy orthologs (SCO) and/or conserved *Plasmodium* proteins (CPP) (grey), across the 14 chromosomes. Breaks in the assemblies (black tick marks) and presence of telomeric repeats (blue circles) are displayed above the chromosome ideograms. Regions of low GC content in PowML03 and PmML03 correspond to multigene family clusters that overlap with domains observed in the Hi-C contact maps.

### Refined reference assemblies with improved contiguity for *P. ovale wallikeri, P. malariae* and *P. falciparum*

Collinearity with published *Plasmodium* genomes was assessed with genome alignment dotplots, using D-Genies [35] which allowed us to orient the chromosomes and manually assign IDs to each assembled chromosome (S2A–S2C Fig). In addition, pairwise whole genome comparisons with best existing references were performed using SyRI [36] revealing extended subtelomeric regions in the new assemblies (Fig 2, S1G Fig). Coverage analysis, calculated in 10 kbp bins, revealed median depths of 167x, 72x, and 150x for PowML03, PmML03, and PfML47, respectively (S2D–S2F Fig). Fewer than 2% of bins fell outside the 1–99 percentile range, consistent with homogeneous coverage distribution across each genome. Amongst bins with low coverage, fewer than 1% had coverage below 25x, corresponding to 16, 33, and 0 bins for PowML03, PmML03, and PfML47, respectively. Mitochondrial copy number was estimated as the ratio of median coverage of mitochondrial to nuclear genomes: the resulting values ranged from 12–86 in *P. ovale wallikeri*, 13–84 in *P. malariae*, and 24–637 in *P. falciparum*, likely reflecting differences in stage composition and associated mitochondrial copy number of the samples (S2F, S2G Fig). Apicoplast copy number estimates were less clear. With the exception of PfML47 (1.68), all samples yielded ratios below 1. *P. ovale* and *P. malariae* were derived from parasite enrichment fractions comprising mixed lifecycle stages, as expected from venous blood. Among the *P. falciparum* samples, PfML47 was derived from a MACS-purified gametocyte fraction, while PfML46 and PfML72 came from mixed infection samples in the fraction which is expected to be dominated by ring stages (S2F, S2G Fig).

In comparison to published references (PowCR01, Pow222, PmUG01, Pf3D7), we noticed a considerable gain in genome assembly span across both *P. malariae* and *P. ovale wallikeri* (Table 1, Fig 2, S2A–S2C Fig). For both these species, a majority of the contiguity gains were in sub-telomeric regions (Fig 2, Table 1, S2A–S2C Fig). For *P. ovale wallikeri*, we saw a reduction in the number of gaps from 1167 (PowCR01) and 182 (Pow222) to 36 (PowML03). With PmML03, 13 gaps remain, compared to 0 gaps in PmUG01 (annotated on Figs 1 and 2). We observed a good contiguity concordance of PfML47 with the *P. falciparum* 3D7 reference and only 7 gaps remaining (vs 0 for 3D7). Chromosome lengths were extended for all chromosomes of PowML03 when compared to Pow222 and 11 of the 14 chromosomes of PmML03 when compared to PmUG01 (S2H Fig), with the biggest increase seen for chr13 (+ 3,100,788 bp) for PowML03 and for chr04 (+ 1,197,753 bp) for PmML03. Further illustrating the completeness of the chromosomes, repeat sequences (GGGTT[T/C]A) [37] associated with *Plasmodium* telomeres were identified at chromosomal ends in a majority of the curated chromosomes, with 14, 22 and 21 loci (of 28 expected) present in chromosomes for PowML03*,* PmML03 and PfML47*,* respectively (Fig 2, S2I Fig). 3, 7, and 8 chromosomes in PowML03, PmML03, and PfML47, respectively, had these repeat sequences on both ends of the chromosome. BUSCO (Benchmarking Universal Single-Copy Orthologs) analysis against the Plasmodium lineage (odb12) showed consistently high scores across the dataset. All three *P. falciparum* assemblies achieved 99% completeness, while PowML03 and PmML3 reached 98.1% and 98.5%, respectively. The remaining *P. ovale* and *P. malariae* assemblies showed similarly high completeness, exceeding 97% (except for PmML07 with 81.3%, which is a secondary assembly derived from a mixed-infection sample) (S1 Table). Altogether, PowML03 and PmML03 constitute the most complete genomes to date for *P. ovale wallikeri* and *P. malariae*, respectively and PfML46, PfML47 and PfML72 represent high-contiguity, chromosome-level assemblies for uncultured *P. falciparum* isolates from Mali.

### Distinct chromosomal architecture and centromere clustering patterns among *P. ovale wallikeri*, *P. malariae*, and *P. falciparum*

Hi-C contact maps used for scaffolding the assemblies for *P. ovale wallikeri*, *P. malariae* and *P. falciparum* exhibit strong chromosome-scale diagonals with internal substructure (Fig 1, S1C-E Fig). The centromere locations in PowML03 and PmML03 were pinpointed using Hi-C contact maps visualised in PretextView, and displayed the characteristic cross-shaped interaction pattern coupled with negative signal indicating reduced interaction with surrounding regions, associated with centromeric domains (Fig 1, S1C–S1E Fig, S2 Table). In PowML03, we see x-like hotspots along all the chromosomes align with the centromere locations (Fig 1A**, example within maroon coloured lines**). For higher eukaryotes, similar signals can be driven by repetitive nature of centromeres, but in *Plasmodium*, no repeats are consistently associated with cross-like patterns in putative centromeres [38,39]. This indicates that the cross-like patterns are likely driven by interchromosomal interactions and strong centromere clustering in *P. ovale wallikeri*, and not sequence similarity. This is consistent with previous Hi-C and microscopy studies in *P. falciparum, P. berghei*, *Toxoplasma gondii and Babesia microti* where centromeres co-localise into a clustered nuclear focus [40–42]. Such clustering is less pronounced in *P. malariae* (PmML03) and *P. falciparum* (PfML47), possibly reflecting weaker Hi-C signal or more dispersed centromere organization in the stages profiled (Fig 1D, 1G). The reduced Hi-C signal observed in PmML03 and PfML47 may reflect differences in sequencing depth, sample composition, or the quality and quantity of material used for Hi-C library preparation, particularly given that the Hi-C data for these assemblies were generated from a mixed-infection sample. Taken together, these patterns suggest that *P. ovale wallikeri* retains a tightly clustered centromere organization, whereas *P. malariae* and *P. falciparum* may display multiple centromere-associated foci or weaker inter-centromeric interactions under the conditions sampled. While chromosomal interactions are not evident in PfML47, we see clear indications of interactions in the subtelomeric regions, highlighted with maroon boxes in Fig 1H.

Across *Plasmodium* species, conserved features of genome architecture include two types. One is three-dimensional nuclear clustering, in which chromosomal regions, both within and from different chromosomes, colocalise spatially within the nucleus. Centromere clustering, for example, refers to the convergence of centromeres from multiple chromosomes at one pole of the nucleus (a Rabl-like configuration). Subtelomeric clustering, described in *P. falciparum*, involves the physical colocalisation of multiple chromosome ends at the nuclear periphery, mediated by subtelomeric repeat elements. The other type refers to clustering of multigene family members within the chromosomes, such as subtelomeric virulence genes [40–42]. In PowML03 and PmML03, the local domain structure from the Hi-C contact maps within each chromosome appears to differ markedly (Fig 1A, 1B, 1D, 1E) and such 3D nuclear organisation can influence multiple aspects of the parasite biology, including the placement of virulence gene families and conserved genes essential for parasite biology within the genome, their transcriptional activity, and their propensity for recombination. In *P. falciparum*, 3D nuclear clustering is functionally relevant, where virulence gene loci on different chromosomes colocalise in the nucleus, and this spatial colocalisation is linked to their coordinated transcriptional silencing [41]. Beyond *Plasmodium*, *Babesia microti* genome shows a Rabl organization with 3D subtelomeric virulence gene colocalisation, whereas *Toxoplasma gondii* lacks virulence gene clustering of either type and is instead dominated by 3D centromeric associations [42].

The delineation of distinct chromosomal domains was further supported by analysis of GC content in PowML03 and PmML03, displaying an isochoric structure, with a marked transition between the relatively GC-rich internal chromosomal regions and the AT-rich subtelomeric regions. (Fig 1A–1F). This is also observed in *P. vivax*, *P. cynomolgi*, and *P. brasilianum* (S1F Fig). The pattern of GC content in PowML03 is similar to *P. vivax* and *P. cynomolgi*, while *P. malariae* is expectedly similar to *P. brasilianum,* which is considered to be nearly identical to *P. malariae* (S1F Fig) [43]. *P. falciparum* shows an inverted pattern, where the extreme ends of the chromosome were GC rich compared to the more AT-rich core (S1F Fig). Furthermore, the centromeres of PowML03 and PmML03, which were identified from Hi-C contact maps, coincide with the steep localised drop in GC content observed within the core genome (Fig 1C, 1F, S2 Table). Because of the low or indistinct cross-shaped interaction pattern in the Hi-C contact maps of *P. falciparum* assemblies, this characteristic drop in GC content, genomic intervals where GC content dropped below 5% across two consecutive 1000 bp bins, was used instead to infer the centromeric regions in *P. falciparum* (S2 Table). This criterion when applied to Pf3D7 is concordant with centromere positions reported earlier [38,39] (S2 Table). Other than the low GC content, the centromeric regions of PowML03 and PmML03 showed no discernible repeat patterns or intra- or inter-chromosomal sequence similarity, mirroring what is seen in *P. falciparum* [38,44].

When interpreting the described Hi-C contact maps, we should account for stage heterogeneity and the specimens used here for scaffolding the resultant assemblies. While PowML03 and PmML03 were each scaffolded with their respective Hi-C data, PfML47 was scaffolded using Hi-C data from another participant, MSC29 (S1A Fig). In such a case, while large-scale interaction patterns are expected to be robust, finer sample-specific structural variations, especially in the subtelomeric regions, should be interpreted with caution. Similarly, stage composition can influence Hi-C signal with each stage presenting a different chromosomal configuration [41,42]. The Hi-C library used to scaffold the *P. falciparum* assemblies was prepared from the enriched fraction of sample MSC29, which contains predominantly early asexual (ring-stage) parasites. On the other hand, *P. malariae* and *P. ovale wallikeri* Hi-C libraries were generated from enriched fractions that contained mixed erythrocytic stages, but largely represented by trophozoites. In *P. falciparum*, chromatin structure has been shown to change dramatically through the erythrocytic cycle, with the Hi-C contact maps of mid-stage trophozoites showing an open and intermingled chromatin conformation compared to early rings, and exhibiting increased inter-chromosomal interactions [41]. Therefore, Hi-C data from a ring-stage enriched sample might underrepresent the configuration we see in trophozoites. In PowML03 and PmML03, Hi-C from a heterogeneous parasite mixture might blur stage-specific contact patterns and show broader, less sharply defined domains. Nonetheless, we do see clear interaction patterns in these two assemblies here, likely due to the high representation of trophozoites in the participants.

### Comprehensive gene annotation of high-quality genome assemblies

Annotation of the improved assemblies with the Companion tool increased the number of coding genes and pseudogenes across nuclear, mitochondrial, and apicoplast genomes compared to the existing best reference annotations [45] (Table 2, Fig 2, S3A–S3C Fig). Companion uses Liftoff [46] to transfer annotations from an existing closely related references (PocGH01, PmUG01, Pf3D7), which resulted in annotations of 5171, 5226 and 5098 genes in PowML03, PmML03, and PfML47, respectively with high sequence identity and alignment coverage compared to the reference in exon regions. A further 1863, 1498 and 680 genes, respectively were annotated by the BRAKER2 [47] implementation in Companion that uses AUGUSTUS [48] and GeneMark [49] to perform *ab initio* gene prediction by integrating the information of protein homology using the translated amino acid sequences to reference databases into the prediction. Non-coding gene prediction in the assemblies was performed with Aragorn and Infernal implementations in Companion, in addition to the Liftoff associated annotation transfer, yielding 130, 122, and 161 genes, respectively. Companion-predicted pseudogenes accounted for the rest of the 952, 550, and 174 annotations respectively for PowML03, PmML03 and PfML47 (Table 2). Of these, 537, 264, and 85 pseudogenes respectively were conserved across the human-infecting *Plasmodium* species and suggest that they most likely represent annotation artefacts arising from sequencing or assembly errors, and need manual curation to correct, pending validation from additional evidence such as orthogonal sequencing data and transcriptomic support.

**Table 2:** Summary of Companion annotations.

			*P. ovale wallikeri*			*P. malariae*		*P. falciparum*	
			PowML03	Pow222	PowCR01	PmML03	PmUG01	PfML47	Pf3D7
**Companion Tools**		Liftoff /RATT, MetaEuk, GenomeTools (for Pow222)	5,144 mRNA,27 rRNA	4,826 mRNA	---	5,203 mRNA,23 rRNA	---	5,051 mRNA,47 rRNA	---
		AUGUSTUS	1,822 mRNA	2,280 mRNA	---	1,450 mRNA	---	676 mRNA	---
		GeneMark.hmm3	41 mRNA	---	---	48 mRNA	---	4 mRNA	---
		Pseudo	952pseudogenes	479 pseudogenes	---	550pseudogenes	---	174 pseudogenes	---
		INFERNAL /Companion (for Pow222)	15 rRNA,7 ncRNA,23 snoRNA,6 snRNA	11 rRNA,7 ncRNA,21 snoRNA5 snRNA	---	10 rRNA,8 ncRNA,22 snoRNA,6 snRNA	---	18 rRNA,31 ncRNA,28 snoRNA,6 snRNA	---
		Aragorn	79 tRNA	75 tRNA	---	76 tRNA	---	78 tRNA	---
**Gene type**		mRNA	7007 (22,3)	7182 (26,4)	6228 (19,1)	6701 (24,3)	5942 (30,3)	5731 (26,3)	5318 (30,3)
		pseudogene	952 (4,1)	479 (4,0)	715 (3,2)	550 (0,0)	637 (0,0)	174 (0,1)	158 (0,0)
	non-coding	noncoding	157 (37,12)	119 (29,0)	104 (18,0)	145 (34,7)	136 (25,9)	208 (40,22)	244 (37,39)
		rRNA	42 (4,12)	11 (0,0)	17 (1,0)	33 (4,7)	26 (2,9)	65 (7,22)	65 (4,39)
		snoRNA	23 (0,0)	21 (0,0)	23 (0,0)	22 (0,0)	20 (0,0)	28 (0,0)	41 (0,0)
		snRNA	6 (0,0)	5 (0,0)	6 (0,0)	6 (0,0)	4 (0,0)	6 (0,0)	5 (0,0)
		tRNA	79 (33,0)	75 (29,0)	58 (17,0)	76 (30,0)	68 (23,0)	78 (33,0)	78 (33,0)
		ncRNA	7 (0,0)	7 (0,0)	0 (0,0)	8 (0,0)	18 (0,0)	31 (0,0)	55 (0,0)
**Gene location**		core	4989	4989	4511	5033	4810	4923	4927
		non-core	3009	874	191	2295	1139	1072	684
		unlocalised/unplaced contigs	39	1763	2302	0	699	26	0
**Gene families**		*fam-m/fam-l*	---	---	---	720(639, 81)	679(483, 196)	---	---
		*pir*	2065(1734, 331)	1774(1584,190)	1189(1189, 0)	221(193, 28)	257(137, 120)	---	---
		*var*	---	---	---	---	---	99(75, 24)	105(65, 40)
		*rifin/stevor*	---	---	---	---	---	205(187, 18)	184(157, 27)
		*sica/stp1/surfin*	95(77, 18)	129(117,12)	78(78, 0)	75(67, 8)	166(109, 57)	10(10, 0)	10(7, 3)
		*etramp*	10(10, 0)	10(10,0)	11(11, 0)	9(9, 0)	8(8, 0)	14(14, 0)	14(14, 0)
		*exp*	101(89, 12)	128(116,12)	105(105, 0)	417(348, 69)	523(314, 209)	102(95, 7)	113(88, 25)
		*phist/resa*	62(56, 6)	62(61,1)	32(32, 0)	35(35, 0)	30(29, 1)	74(63, 11)	77(58, 19)
		*rbp*	16(13, 3)	16(12,4)	7(7, 0)	6(4, 2)	5(3, 2)	7(7, 0)	7(5, 2)
		conserved	1276(1138, 138)	1364(1310,54)	1696(1696, 0)	1286(1191, 95)	1272(1271, 1)	1284(1261, 23)	1256(1256, 0)
		hypothetical	826(659, 167)	495(485,10)	467(467, 0)	858(774, 84)	54(54, 0)	493(455, 38)	6(6, 0)

Companion annotation statistics of PowML03, PmML03 and PfML47 in comparison to the existing reference annotations. “Gene type” section counts in brackets indicate organellar genomes (chrA, chrM). “Gene families” section counts in brackets indicate (coding genes, pseudogenes). “Core” and “non-core” regions of the chromosomes were defined by the boundaries of the outermost position on each chromosome end of a 1:1 ortholog conserved across all human-infecting *Plasmodium* species, with the “non-core” regions corresponding to the sub-telomeric regions. “Unplaced” contig refers to unassembled scaffolds with known chromosomes, while “unlocalised” refers to those with unknown chromosomes. The categories of gene families displayed in the table are described in **Methods**.

In *P. falciparum*, in addition to the well-characterized *var* gene family, a number of additional loci were annotated as “hypothetical proteins” beyond the *var* genes close to the telomeric ends (S3H Fig). These short genes lack any identifiable domains. Long non-coding RNAs derived from subtelomeric regions have been reported previously [50,51]. However, these “hypothetical proteins” are characterised by short exons (median of 114 bp) and showed strong signatures of repetitive sequences in both the transcribed sequences and the resultant protein sequences, with high prevalence of several copies of repeat motifs (S3I, S3J Fig). This suggests that many of these predicted gene models may in fact represent artifacts arising from repetitive genomic regions rather than protein-coding sequences. Overall, by integrating homology-based transfer with *ab initio* prediction, the assemblies of PowML03, PmML03 and PfML47 captured a total number of 8116, 7396, and 6113 genes respectively, comprising both conserved and lineage-specific genes.

Subtelomeric regions of *Plasmodium* genomes typically harbor rapidly evolving, lineage-specific gene families associated with immune evasion and host–parasite interactions. These multigene family proteins were annotated at the sub-telomeric regions in our new assemblies, whereas in previous genome assemblies, contigs containing these gene families were largely unlocalised to chromosomes. In *P. ovale wallikeri* (PowML03), these regions were enriched in the *Plasmodium* interspersed repeat (*pir*) genes, the surfin-related subtelomeric protein 1 (*STP1*) family, the *SICAvar* family, and the *phist* family. We identified 2065 (1734 protein coding, 331 pseudogenes) *pir* genes that comprise about 25% of the genome’s total gene content (Table 2, Fig 2B). In contrast, *P. malariae* (PmML03) subtelomeric regions contained *fam-l* and *fam-m* genes, as well as *pir, STP1*, *SICAvar*, and *phist* gene families consistent with previous reports from other species and earlier incomplete assemblies (Fig 2D, Table 2). *fam-m* and *fam-l* genes, which are unique to *P. malariae*, comprise 720 of the 7396 genes in the PmML03 assembly and are presumed to be involved in host-parasite interactions (Table 2). Genes termed “*Plasmodium* exported protein, unknown function” are enriched in the non-core regions of both PowML03 and PmML03, and are highly expanded in PmML03 numbering more than 400. In *P. falciparum* (PfML47), subtelomeric regions were dominated by the *var* gene family, together with *rifins, stevor*, *STP1*, *SICAvar*, and *phist* gene families as expected (Fig 2F, Table 2).

In both PowML03 and PmML03, the distinct domains in chromosome organization observed in Hi-C contact maps, as well as patterns in GC content, are further reflected in the distribution of multigene families and single-copy orthologs (SCOs) or conserved genes (Fig 1 and 2). SCOs and conserved genes across *Plasmodium* species, in particular those labelled “conserved”, were present almost exclusively within the core genome (Fig 2) within central chromosomal blocks in Hi-C contact maps. Genes from multigene families such as *pir*, *fam-m/l, STP1*, *SICAvar*, and *phist* were clustered in the subtelomeric regions, aligning with the outer domains of the Hi-C contact maps, with the lengths of these outer domains corresponding closely to the subtelomeric regions harboring these gene families. In contrast to the *var* genes in *P. falciparum*, we did not observe internal localisation within the core genome of any of these multigene family members in *P. ovale wallikeri* and *P. malariae*. This partitioning reflects a “two-speed genome” model, described in several plant pathogens such as aphids, phytophthora and fungi [52–56], where a conserved core encodes essential parasite biology, while subtelomeric ‘adaptive zones’ harbour rapidly evolving multigene families involved in host-parasite interactions. The gene distribution provides complementary evidence for distinguishing core from non-core regions in the assemblies, and we defined the boundaries of the core genome for PowML03 and PmML03 by the outermost position on each chromosome end of a 1:1 ortholog conserved across all human-infecting *Plasmodium* species (Fig 2B, 1D, S2 Table).

In both PowML03 and PmML03, the skewed AT content within the sub-telomeric regions across the chromosomes appears to be mainly driven by the intronic regions of the multigene families, as well as the intergenic regions (S3D–S3E Fig). In *P. falciparum*, the *var*, *stevor* and *rifin* mulitigene families contribute to a higher GC content compared to the rest of genes, especially within their exonic regions (S3F Fig). In addition to the gene body, intergenic regions of PowML03 and PmML03 were equally low in their GC content in the sub-telomeric regions compared to the core genome (S3G Fig).

### Sub-telomeric gene families in *P. ovale wallikeri* and *P. malariae*

*P. ovale* spp. and *P. malariae* species exhibit striking expansions in gene families potentially linked to immune evasion and host interactions, particularly the *Plasmodium* interspersed repeat (*pir*) superfamily in *P. ovale* spp. and the *fam* genes in *P. malariae* [1,2,26,28]. These expansions highlight their distinct evolutionary trajectories and studying them can provide insights into the mechanisms underlying chronic infection, immune evasion, and host adaptations in these neglected malaria species. Gene structure and exon organisation of these multigene families have been described in earlier assemblies, and our findings described below show consistency with these prior reports while also identifying a greater number of these genes, reflecting the improved completeness of the new assemblies. In contrast, GC content patterns across *pir* and *fam* loci have not been well explored in previous work. Moreover, because our assemblies are resolved at the chromosome level, we were able to examine the chromosome-specific distribution and clustering of *pir* and *fam* loci both within and across the sequenced samples, which was not previously possible with more fragmented assemblies..

The *Plasmodium* interspersed repeat (*pir)* gene family has undergone extensive lineage-specific expansions in *Plasmodium* species, giving rise to a large and heterogeneous repertoire, ranging from ~200 in *P. chabaudi* [57], 170–224 *pirs* in *P. berghei,* [58] to over 2,100 in *P. ovale wallikeri* (PowML03) as shown here (Table 2). These expansions in PowML03, concentrated in subtelomeric regions, contribute to a ~ 30% increase in genome size compared to other *Plasmodium* species (Table 2, Fig 2B). *P. ovale wallikeri pir* genes were distributed across all chromosomes, showing no chromosome-specific clustering (Fig 2B). Transcript lengths of predicted protein coding *pirs* range from 198 to 2664 bp, with exon counts from 1 to 7 (S4A Fig). Typically, *pir* genes lack a signal peptide, the N-terminal amino acid sequence that directs proteins to the secretory pathway (S4A Fig). They contain three exons: a short first exon, a longer second exon with a transmembrane (TM) domain, and a shorter third exon (Fig 3A, 3B, S4A Fig).

**Fig 3:**
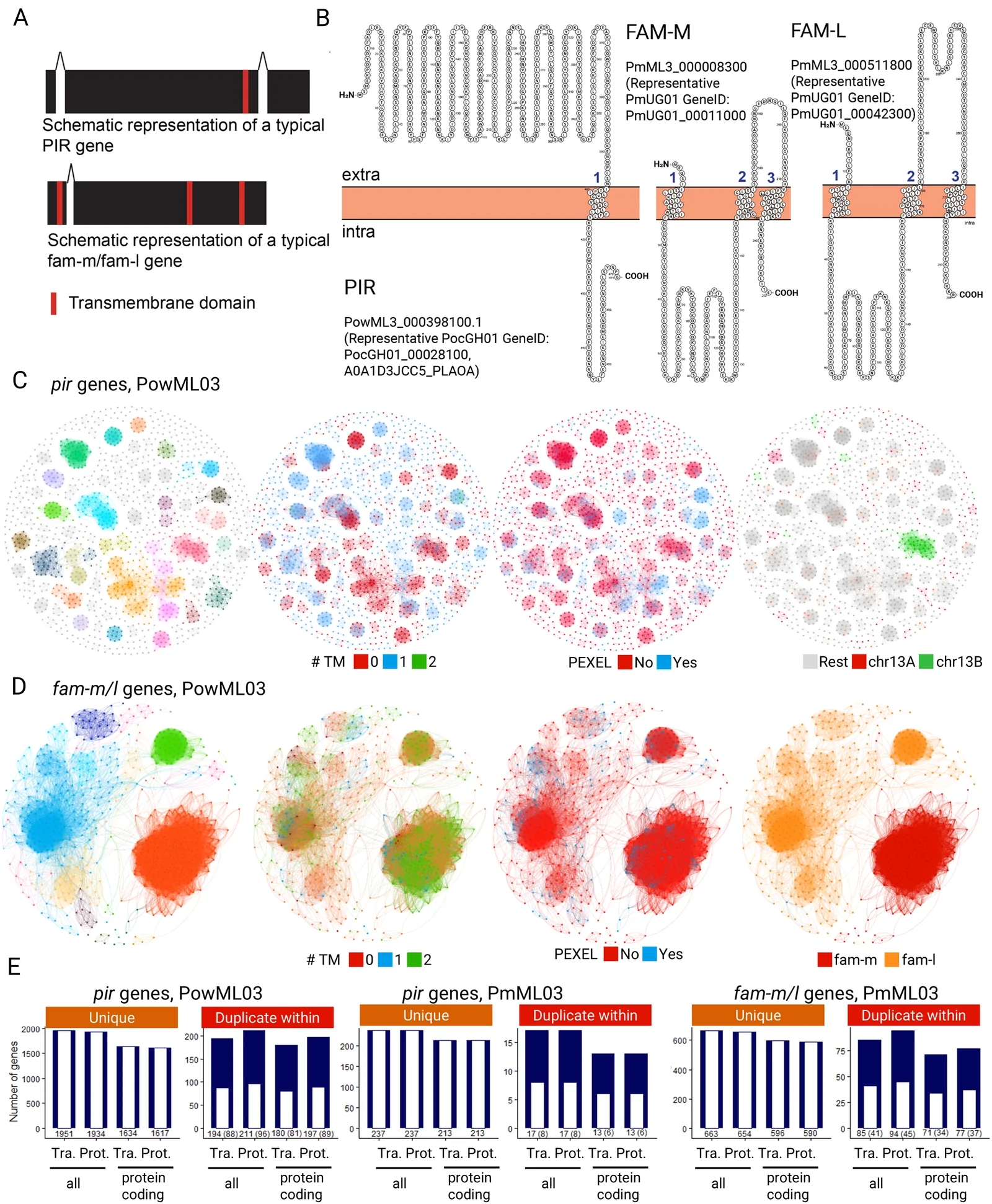
*pir* and *fam-m/l* gene families in *P. ovale wallikeri* and *P. malariae.* **A,** Typical exonic structures of *pir* genes in *P. ovale wallikeri* (PowML03) and *P. malariae* (PmML03), and *fam-m/fam-l* genes in *P. malariae* (PmML03) (black rectangles as exons, with red block indicating transmembrane domain). **B,** Example of topology of a typical PIR generated from Protter using the protein sequence ID corresponding to the sequence in *P. ovale curtisi* protein, PocGH01_00028100. **C,** Protein similarity network of PIRs in PowML03. Coloured by module, by number of transmembrane domains (TM), by presence of relaxed PEXEL motif (RxLxx[EQD] or RxLx[EQD]), and by *pir* genes location in chr13 (indicated in S5 Fig). **D,** Protein similarity network of the FAM-M/L proteins in PmML03. Coloured by module, by number of transmembrane domains (TM), by presence of PEXEL motif (relaxed motif R.L..[EQD]), and by gene description. **E,** Plot showing the total number of genes grouped by whether transcript (Tra) or amino acid (Prot) sequence is unique or duplicated within the sample. White bars within quantify the number of unique sequences amongst the duplicated sequences. Data is shown for both “all” genes and only “protein coding” genes of the indicated families.

### Pirs in *P. ovale wallikeri* and *P. malariae*

Protein similarity networks were constructed from all-versus-all BLASTP searches of *P. ovale wallikeri* (PowML03) amino acid sequences from all 1807 protein-coding *pirs*, retaining 1381 PIRS with ≥50% identity over ≥100 aligned residues. The resulting networks were visualized in Gephi [59] using a force-directed layout to reveal relationships among these multigene family members (Fig 3C). The *pir* family formed a diffuse network, with several distinct clusters, suggesting functional specialisation within the family. Furthermore, self alignment of the PowML03 subtelomeric regions revealed extensive sequence similarity across the non-core regions likely suggesting ongoing segmental duplication (S5A,D Fig). In addition, two distinct regions were evident on the uncharacteristically long *pir*-filled arm of chromosome 13, which appear to be distinct and dissimilar to the rest of the regions (S5D Fig). This is further reflected in the protein similarity network, where the subset of PIRs from the long arm of chromosome 13, grouped into a clear cluster, probably indicating local expansion events.

*P. ovale* and *P. malariae* encode all the components of the PTEX (*Plasmodium* translocon of exported proteins) machinery described in *P. falciparum* and *P. berghei*, which is involved in export of proteins to the host cell. A subset of PIR proteins contain the canonical PEXEL (*Plasmodium* export element) motif that is required for protein export beyond the parasitophorous vacuole membrane (S4A Fig), and are likely exposed on the surface of infected red blood cells, where they could play key roles in antigenic variation and immune evasion [60,61]. The protein topology prediction suggests that the larger region before the TM-domain is presented on the cell surface while the shorter C-terminal portion of the protein is oriented toward the interior of the host cell, potentially important for trafficking to the RBC surface or for downstream signaling events within the infected red blood cell (Fig 3B). Most *pir* genes contain a ~ 164 bp exon located downstream of the transmembrane region, and the C-terminal region of PIR proteins shows increased sequence conservation, consistent with a conserved functional role. However, most PIRs lacked the PEXEL motif, suggesting that not all are exported, or involve non-PEXEL mediated export.

The pirs in *P. malariae*, also display an expanded repertoire, albeit much less than *P. ovale* or *P. vivax* (Table 2, Fig 2D, S4B,C Fig), with 254 *pir* genes in PmML03 (226 protein coding, 28 pseudogenes). They display an exonic structure similar to those in *P. ovale wallikeri* and distributed chromosomal location (Fig 2D, S4B Fig). Protein similarity network comprising *pir* genes from both PmML03 and PowML03 show distinct, non-overlapping clusters for each species, indicating substantial sequence divergence among pir genes across these two species (S4D Fig).

We assessed sequence redundancy within the *pir* gene family in PowML03 across the chromosomes by classifying the transcript and protein sequences into unique singletons and those duplicated within the specimen. We noticed sequence redundancy within PowML03 across the chromosomes, identifying 194/2145 *pir* gene transcripts, comprising 88 unique sequences that were present in multiple (2–4) identical copies, indicative of recent tandem or segmental duplication events (Fig 3E). Likewise, within the *pirs* in PmML03, 17/254 (comprising 8 unique copies) occur in multiple (2–3) identical copies.

### fam-m, fam-l genes in *P. malariae*

The 720 fam-m/l genes were similarly concentrated in subtelomeric regions, along with other surface family genes, and show no chromosome-specific localisation (Table 2, Fig 2D). Transcript lengths of protein coding fams range from 252 to 805 bp for fam-m and 95–937 bp for fam-l genes (Fig 3A, S4E Fig). Both fam-m and fam-l genes range from one to four exons, with the most typical exonic structure (~ 93% of the genes) being two exons with a short first exon containing a transmembrane domain and a long second exon with two TM domains. Only a subset of *fam-m* and *fam-l* proteins (28 and 43, respectively) are predicted to contain an N-terminal signal peptide (S4E Fig). Around 10% of the *fam-m/l* genes harbor the PEXEL motif and are likely exported to the host cell (S4E Fig) [26]. The protein topology suggests a large fragment of the protein (between the first and second TM) is intracellular while a short segment between TM2 and 3 is presented on the cell surface with a short C-terminal tail within the cytoplasm (Fig 3B). fam-m/l proteins show structural homology with PfRh5, which is key for RBC invasion, and they may have a role in host cell binding [26]. Protein similarity networks were clustered from all-versus-all BLASTP searches of *P. malariae* (PmML03) protein-coding fam-m/fam-l genes (667), retaining sequence pairs with ≥50% identity over ≥100 aligned residues. The network comprising 268 genes showed clear and well-separated clusters of fam-m and fam-l sequences, as expected based on family-level classification (Fig 3D). Within the fam-l group, two major clusters were evident, suggesting the presence of distinct fam-m subfamilies or divergent lineages (Fig 3D). No clustering patterns were associated with chromosomal location or number of transmembrane domains, indicating that *fam* gene similarity primarily reflects sequence divergence rather than genomic context or structural features (Fig 3D). Similar to *pir* genes, within-genome redundancy of *fam* transcripts and derived amino acid sequences was notable. We identified 85/748 *fam-m/l* transcripts, comprising 41 unique sequences that were present in multiple (2–4) identical copies.

### Syntenic analysis & recombination within sub-telomeric regions

High quality assemblies from multiple specimens enabled us to assess synteny within and across *Plasmodium* species, using Circos and SyRI/plotsr [36,62,63] (Fig 4, S6–S9 Fig). The new reference assemblies, PowML03 and PmML03, were each scaffolded using Hi-C data derived from their respective specimens. The additional assemblies (PowML04, 08, 09 and PmML04, 07), however, were scaffolded using Hi-C data from the same specimens used for PowML03 and PmML03, respectively. In contrast, the *P. falciparum* assemblies, PfML46 and PfML47, were scaffolded using Hi-C data from a different participant, MSC29, which also yielded the PfML72 assembly (S1A Fig). Caution should therefore be exercised when interpreting several assemblies, as the Hi-C data originating from a different individual and developmental stage may influence contact patterns and thereby the final assemblies. Although minor scaffolding inaccuracies cannot be fully excluded, the overall correctness of the assemblies is supported by the strong conservation of synteny across core genomic regions, including those spanning scaffold joins (Fig 4A, S6C–S6E and S7A–S7C Fig). Contigs from earlier published references and other specimens from this study that could not be localised to chromosomes primarily map to the sub-telomeric regions of PowML03 and PmML03, and are typically AT-rich (S6D–S6F Fig). Two large unlocalised contigs in PowML04 and PowML09 correspond to the uncharacteristically long sub-telomeric region of chr13 in PowML03 (S6D Fig).

**Fig 4:**
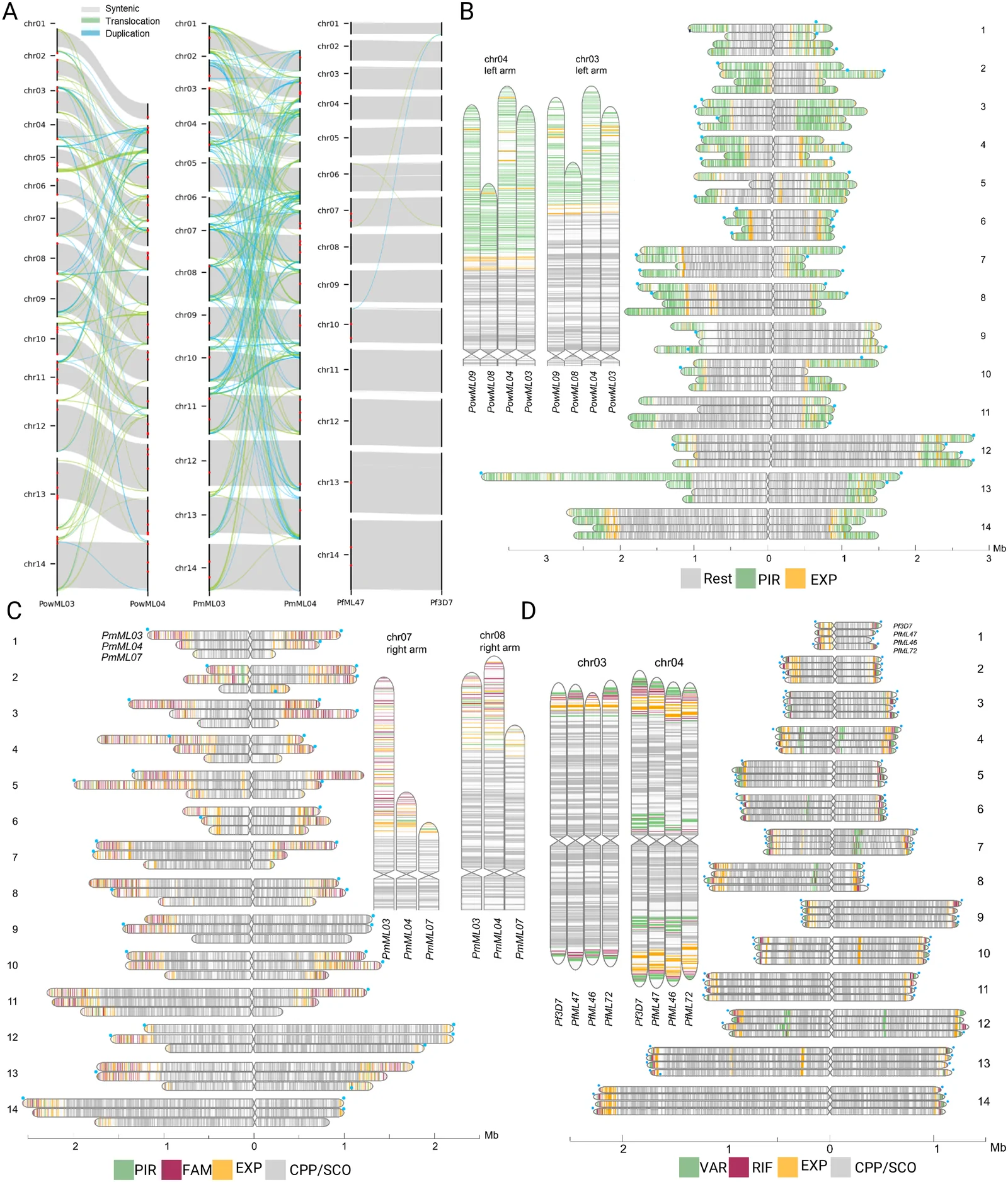
Syntenic analysis and dynamic recombination within sub-telomeric regions. **A,** Synteny plots between the two best genome assemblies available for each species shows synteny within the core genome and a high level of sequence movement within the subtelomeric regions in *P. ovale wallikeri* and *P. malariae*. **B-D,** Comparison of gene annotations across all assemblies generated here show a high degree of gene order conservation within the core genome, which is lost towards the subtelomeric regions. A close up of chromosomal ends is shown in the insets. This syntenic order within the core genome is less apparent in PmML07 due to the comparatively poorer quality of the assembly. Synteny is also disrupted around the internal *var* genes found in chromosomes 4, 7, 8, and 12 of *P. falciparum*. Blue circles correspond to the presence of *Plasmodium* telomeric repeats, indicating likely chromosomal ends. Genes are grouped into the displayed gene families into categories as described in **Methods**.

To assess sequence redundancy and conservation across isolates (i.e., the different genomes) of the expanded *pir* and *fam-m/l* multigene families, we classified genes across the annotations into three categories based on transcript and protein sequence identity: singletons, duplicates within a single isolate, and sequences shared across multiple isolates. Across PowML datasets, most protein and transcript sequences were identical across two or more isolates, followed by unique singletons. A pronounced fraction of transcript sequences showed redundancy both within, as described earlier for PowML03, and across the isolates (S6G–S6I Fig). For example, in PowML03, 1520 *pir* gene sequences were shared across two or more isolates, 199 occurred as copies both within an isolate and in at least one other isolate, and 12 were present only as within-isolate copies (S6G Fig). The relative contribution of these categories varied across datasets, reflecting differences in assembly completeness annotation quality, with the best assemblies of *P. ovale wallieri* (PowML03 and PowML09) showing high proportion of shared sequences due to increased gene annotation. Remarkably, the proportion of sequences shared across isolates was higher in *P. ovale wallikeri* compared with *P. malariae*, indicating low sequence variation (S6G–S6I Fig). Recent population genomic studies indicate that both *P. ovale wallikeri and P. malariae* exhibit lower nucleotide diversity compared to *P. falciparum* and *P. ovale curtisi* [1,2]. However, a head-to-head comparison between these species, particularly from this geographic region, is lacking.

Extensive nucleotide sequence redundancy observed within the *pir* and *fam-m/l* multigene families complicates the interpretation of alignment-based synteny analyses like SyRI. As many of these genes are highly similar to each other or even occur as multiple identical copies dispersed across the genome, synteny tools struggle to resolve duplicated or interchangeable loci. To increase confidence in the accurate identification of multicopy sequences, we restricted interpretation to SyRI-defined blocks that are > 10 kb in length (longer than most genes) and show >98% identity to each other, and only these high-confidence sequences were explored and visualised. We observe clear synteny across the core genome and extensive translocation and duplication in the non-core genome for both *P. ovale wallikeri* and *P. malariae* (Fig 4A, S7A Fig).

Additionally, we used gene annotations to assess and quantify the dynamics between genomes in the AT rich sub-telomeric regions. To minimise artefacts arising from annotation transfer, stringent filtering criteria were used: only genes present as single copies (no two genes with identical nucleotide sequence of the gene body) in the assemblies PowML03 and PmML03 were considered, and only those successfully transferred by Liftoff with high confidence (coverage > 0.9 and sequence identity > 0.8) were retained for downstream analyses. We define a block as a contiguous set of orthologous genes (minimum of two genes) that maintain relative order in the chromosome of one genome but were relocated in another. We identified several blocks that have shifted positions between chromosomes, representing transposition events (S7D–S7G, S8 and S9 Fig). For example, in *P. malariae*, one block on chromosome 4 in PmML03 (spanning genes PmML03_000122500 - PmML03_000123400) is instead found on chromosome 11 in PmML04 (S9A Fig). This replicated block consists of 10 genes, with clear one-to-one correspondences between the source and target chromosomes, with synteny lost beyond this block. We find transposed blocks containing up to 30 genes in *P. ovale wallikeri* and 20 in *P. malariae* which typically include members of *pirs* and *fams*, suggesting potential functional implications for parasite adaptation and antigenic variation (S7D, S7E Fig). In *P. falciparum*, we find a maximum of 3 genes in these blocks, which consist mostly of rifins and exported proteins (S7F Fig). Several more detailed examples for each species of block transposition are supported using both JBrowse [64] and SyRI [36] (S8 and S9 Fig).

For a broader visualisation of the loss of synteny in the sub-telomeric regions in these species, Companion-annotated genomes were oriented by their predicted centromere location (Fig 4B–4D, S2 Table). For all within species comparisons, we see high level of synteny within the core genome, supported by a similar pattern of conserved genes (single copy orthologs across human infecting *Plasmodium* species, and genes labelled as “conserved *Plasmodium* protein” depicted with grey lines) (Fig 4B–4D). However, this synteny is lost outside the core genome. Similar gene rearrangements in the subtelomeric regions were described for *P. chabaudi* [57]. Dynamic subtelomeric regions in *P. falciparum* are well‑documented, especially in relation to the *var* gene family, which undergoes frequent ectopic recombination, facilitating antigenic variation [65–67]. Comparable dynamics are evident here in *P. ovale wallikeri* and *P. malariae*, although the extent of this variation is striking given that these parasites were collected from the same village in a single malaria season. This observation underscores the capacity for rapid diversification in sub-telomeric regions even among closely related parasite populations in these two species.

Moreover, the extent of gene annotation discrepancies, upon transferring annotations from PowML03 and PmML03 to the other assemblies, within the multigene families compared to the conserved genes further points towards the extensive recombination events within the sub-telomeric regions, highlighting the rapidly evolving multigene families (**S6A**, **S6B Fig**).

### Other gene families, drug resistance markers, exported proteins

In addition to the two major gene families analysed above, we also surveyed other multi-copy and antigenic gene families across our assemblies, including SURFIN/STP1, MSPs, and genes commonly used as markers of invasion or sexual-stage biology (e.g., Pfg25/27). The assemblies PmML03 and PowML03 exhibit a dramatically expanded repertoire of SURFIN/STP1-type genes (~90–120 copies), in sharp contrast to the ~ 10–12 copies typical of *P. falciparum* (Table 2). SURFIN/STP1 proteins are implicated in host–parasite interactions, cytoadherence, and immune evasion. Similarly, substantial copy-number variation in MSPs and Pfg25/27 genes was observed in both *P. malariae* and *P. ovale wallikeri*. For example, we detect 14 tandem copies of MSP3 in PowML03 (chromosome 10) and 13 tandem copies in PmML03 (chromosome 6) (S10A Fig). MSP3 family members play roles in merozoite invasion and are known vaccine targets. The differences are equally striking for Pfg25/27, a key marker of gametocytes crucial for gametocytogenesis (S10B Fig). While *P. falciparum* (Pf3D7) and *P. ovale wallikeri* (PowML03) encode only 1–2 copies, PmML03 contains a large expansion of 24 copies (22 in PmML04 and PmUG01, 15 in PmML07) all arranged consecutively on chromosome 14 (S10B–S10C Fig) in addition to the single copy on chromosome 4 that is more closely related to the *P. falciparum* and *P. ovale* spp. orthologs. Such expansions suggest that *P. malariae* and *P. ovale wallikeri* may depend on a far broader set of exported or surface-exposed antigens. This could reflect differences in their strategies for immune modulation, chronicity, or host cell tropism. These patterns also highlight important implications for future diagnostics, where gene family expansion could influence molecular assay performance.

We also assessed known drug-resistance associated loci in *P. falciparum*. The triple *dhfr* mutant haplotype N51I/C59R/S108N, widely prevalent in Mali and often found close to 100%, was present in all three *P. falciparum* specimens sequenced here (S10D Fig). This is consistent with regional patterns of sustained SP drug pressure for intermittent preventive treatment in pregnancy. We did not observe evidence of amplifications, deletions or copy number variations at mdr1 or crt loci or plasmepsins. Two of the three *P. falciparum* specimens (PfML47 and PfML72) harbor the CVIET (chloroquine resistant) haplotype across residues 72–76, along with additional mutations A220S, Q271E, I356T, and R371I, which have been shown to contribute to the resistance phenotype [68,69] (S10E Fig). PfML46 and PfML72 carry the K189T mutation in *kelch13*, a common non-propeller polymorphism identified across Africa and Asia [70]. This mutation has been shown to be associated with a longer parasite clearance time in patients treated with Artemisinin-based Combination Therapies (ACTs) [71]. On the other hand, PfML47 isolate carries the S436A and G437A mutations in *dhps*, which are implicated in conferring resistance to sulfadoxine, particularly when occurring alongside other *dhps* or *dhfr* mutations [72]. While none of the *P. ovale wallikeri* or *P. malariae* specimens from this study harbored mutations in the above positions (except for the A437 residue in *dhps* existing as uniformly wild-type), data on drug-resistance markers in these non-*falciparum* species remain scarce. Genomic studies of *P. malariae* and *P. ovale* isolates across Africa and Asia identified mutations associated with reduced pyrimethamine susceptibility [23,73]. There is growing concern over emerging resistance, with a recent study from Mali identifying unusually high IC50 values for chloroquine, lumefantrine and artemether against *P. malariae* isolates, which are the current frontline drugs against *P. malariae* infections [74]. These reports highlight the need for comprehensive surveillance and resistance studies of these neglected species.

## Discussion

In this study, we generated substantially improved reference assemblies for *Plasmodium ovale wallikeri* (PowML03), *Plasmodium malariae* (PmML03) using PacBio ULI HiFi long read sequencing and Hi-C from very little starting material from natural infections. Producing reference-grade genomes directly from low-input clinical material bypasses the need for lab culturing that can be associated with artefacts and adaptations [75]. These assemblies offer more complete annotations of coding genes, pseudogenes, and non-coding elements across the nuclear, mitochondrial, and apicoplast genomes.

The genome of *P. brasilianum*, a simian infecting species considered genetically and biologically close to *P. malariae*, has been recently sequenced and assembled using PacBio from *ex vivo* schizont material [43]. Despite the significant amount of starting material and similar long-read technique, the assembly is similar to the existing PmUG01 and incomplete within the sub-telomeric regions. This highlights the utility of Hi-C data to obtain complete assemblies that better capture the full genomic complexity of these species, especially because of the repetitive nature of the sub-telomeric region genes. Hi-C data enabled scaffolding of 50 and 23 hifiasm contigs from PowML03 and PmML03, respectively into the 14 chromosomes of the final assembly. Altogether, PowML03 and PmML03 constitute the most complete genomes to date for *P. ovale wallikeri* and *P. malariae*.

These resources also open the door to more refined population, comparative, and functional genomics. Population genomics of these species remains in its infancy. Recent work has found limited structure in *P. ovale* spp. populations but evidence for differentiation in *P. malariae* [1,2]. The improved annotation sets and genomes will be especially valuable for designing accurate species-specific assays to better detect and monitor *P. ovale* and *P. malariae* in genomic surveillance programmes, an urgent need given their underappreciated epidemiological impact. Sexual development is poorly characterised for *P. ovale* spp. and *P. malariae*, especially with regard to regulators (e.g., AP2-G, GDV1), and developmental niches. These gaps underscore the need for stage-resolved single-cell studies to uncover conserved and species-specific regulatory strategies across the life cycles. The large, highly repetitive subtelomeric gene families we’ve identified here may complicate read mapping and quantification. Standard short-read RNA-seq methods often collapse or misassign expression among paralogues, masking true transcriptional diversity. Future projects may benefit from long-read transcriptomics, repeat-aware mappers, and curated gene family databases, to accurately resolve expression patterns across expanded multigene families. The chromosome-scale assemblies generated in this study will be a valuable foundation for such work, providing accurate gene models and complete subtelomeric regions for interpreting transcriptional dynamics in future bulk and single-cell transcriptomic analyses.

The improved resolution of long repetitive tracts here enabled our investigation of large structural variants and copy-number changes. Our findings reinforce a common theme across *Plasmodium* species, which display a highly conserved and syntenic core genome in contrast to highly dynamic subtelomeric regions enriched in rapidly evolving multigene families. We see pronounced lineage-specific expansions, with *pir* genes in *P. ovale* spp. and *fam-l/fam-m* genes in *P. malariae*, alongside *stp1*, *sicavar* and *phist* families, mirroring the compartmentalisation seen in *P. falciparum* and *P. knowlesi*, where subtelomeric diversification drives immune evasion while the core genome maintains essential functions. Sharp transitions in GC content and conserved orthologue boundaries further underscore this stable-core, flexible-subtelomere architecture.

Although the mechanisms underlying the extensive subtelomeric dynamism in *P. malariae* and *P. ovale* spp. remain unclear, they echo and potentially exceed patterns in *P. falciparum*, where *var* gene diversification is driven by frequent ectopic recombination and sequence exchange during mitosis [67,76]. This raises the possibility that processes that shape *var* dynamics may also shape *pir* and *fam-m/l* evolution. Given that mutually exclusive expression of *var* genes underpins antigenic variation in *P. falciparum*, the presence of large subtelomeric families in *P. malariae* and *P. ovale* spp. suggests that similar regulatory mechanisms may operate, highlighting *P. malariae* and *P. ovale* spp. as promising systems for future investigation into the mechanisms driving this sub-telomeric dynamism. Future transcriptomic and epigenomic studies, particularly at single-cell resolution, will be critical for determining whether these expanded repertoires are actively regulated, stage-specific, or partly pseudogenised. Their large numbers also raise intriguing evolutionary questions. Expansions in *P. ovale wallikeri* and *P. malariae* may reflect broader host ranges or cross-species transmission potential, as suggested by reports of *P. brasilianum* in primates and *P. ovale*-related parasites in chimpanzees. Dissecting the functional and regulatory diversity within these multigene families will be central to understanding antigenic variation, chronic infection, and host adaptation in these understudied parasites.

Together, these assemblies advance both the technical and biological frontiers. Technically, they demonstrate that ULI and long-read sequencing can generate high-quality *Plasmodium* references from minute DNA quantities, a crucial feature when working with scarce clinical samples. Biologically, they highlight conserved genome organisation principles across *Plasmodium* species while revealing unique lineage-specific adaptations. We recommend that the two new assemblies for *P. ovale wallikeri* and *P. malariae* be added to VEuPathDB and adopted as the new reference genomes, replacing the current reference assemblies hosted on the database. These resources will underpin comparative, functional and population level studies, enable improved molecular diagnostics, and strengthen collaborative initiatives such as the The Pan African Vivax and Ovale Network (PAVON).

## Methods

### Ethics statement

The study protocol and informed consent forms were reviewed and approved by the Comité d'Ethique des Facultés de Médecine/d'Odontostomatologie et de Pharmacie FMOS/FAPH, at the University of Sciences, Techniques, and Technologies of Bamako, in Bamako, Mali, and and by Malian regulatory authorities (No.2020/274/CE/FMOS/FAPH). The study was also consulted and approved by local administrative representatives and the village elders, and written informed consent was obtained from all the adults and from all the parents or guardians of the children. The research use and processing of the samples is described in the Research Contract Agreement No. 20–070 between the MRTC-USTTB (Malaria Research and Training Centre - Université des Sciences Techniques et Technologiques de Bamako) and Wellcome Sanger Institute.

### Genomic material and assembly generation

#### Sample origin and processing.

Study participants aged 6–19 with asymptomatic malaria or those admitted to clinics with uncomplicated malaria infections from Faladie, Mali were included in the study. For this analysis, participants with *P. ovale*, *P. malariae* or *P. falciparum*, either as a single or mixed infection with other species, as determined by light microscopy, were included in the study. Informed consent was taken from the patients to obtain 6 mL of blood. Blood was drawn into a CPDA vacutainer tube. Samples were processed according to the protocol described here [77]. Briefly, 3 mL of blood from the CPDA tube was mixed with 5mL of SA buffer (10 mM Tris-HCL, 166 mM NaCl, 10 mM Glucose, pH 7.37) and passed through a chilled MACS LS column to obtain late asexual stages and gametocytes. The flowthrough was passed twice through pre-PBS-washed Plasmodipur filters to remove leukocytes. This flowthrough was further washed with PBS before 100 µl of the pellet was subjected to Streptolysin-O lysis to remove uninfected RBCs, to obtain FT-SLO fraction. We observed variable lysis with SLO across the study participants, thus ending up anywhere from ~5 µl to ~100 µl of the fraction. The remaining flowthrough was lysed with 0.2% saponin, washed thrice with PBS before pelleting to obtain the FT-SAP fraction. The enriched fractions were profiled for single cell transcriptomics (not pertaining to this study) and the remaining material, if any, was stored at -80°C until DNA extraction. The fractions used for generating the assemblies are listed in S1A Fig.

All parasite enrichment was performed on-site in Mali. Frozen aliquots, stored at -80°C, were then shipped to the Wellcome Sanger Institute on dry ice for DNA extraction and sequencing

#### DNA extraction and Sequencing.

High molecular weight (HMW) DNA was extracted from enriched parasite fractions (listed in S1A Fig) using Qiagen’s MagAttract HMW DNA extraction kit on a KingFisher Apex Automated Extraction System from Thermo Fisher Scientific, and eluted in a volume of 100 μl. HMW DNA was sheared using the Covaris g-TUBE by passing the DNA through the g-Tube twice at 3500 rpm to obtain ~10 kb fragments required for library preparation. DNA quantity measured with a Qubit Fluorometer using Qubit’s dsDNA High Sensitivity Assay kit. Fragment size distribution was evaluated on the Agilent Femto Pulse system (S1A Fig). In case of presence of shorter DNA fragments, sheared DNA was further purified and concentrated using 3.7X Ampure XP SPRI to remove fragments below 3kb.

PacBio HiFi DNA sequencing libraries were constructed according to the manufacturers’ instructions. Both lowInput (LI) and Ultra LowInput (ULI) libraries were prepared using PacBio SMRTbell Express Template Prep Kit 2.0 and PacBio SMRTbell gDNA Sample Amplification (noting only ULI libraries were successful, as described below).

Of the 25 samples processed using the above DNA extraction and shearing, 7 samples (those with >250ng sheared product in a volume of 46 µl) underwent library preparation with the LI protocol and the other 18 samples (<250ng sheared product, 46 µl) underwent the ULI library protocol. We attribute this disparity in quantity to the varying amounts of host white blood cells and DNA across the sampled fractions that were retained in the MACS column or were not filtered out by the Plasmodipur columns. In the first batch, 7 LI samples and 11/18 ULI samples were multiplexed together on a single SEQUEL IIe flow cell each. The other 7 ULI samples were sequenced in a separate batch, sharing one 1/5 of a Revio flow cell. *Plasmodium* genomes were typically 20–30 Mb and these were multiplexed to generate much higher coverage than the 30x required for assembly in order to overcome substantial human DNA contamination. All 7 LI samples and 10/18 ULI samples failed due to high human contamination and thus low parasite content. However, the remaining 8 ULI samples produced sufficient parasite data, resulting in assemblies with high coverage.

Hi-C data were generated from SLO fractions of participants MSC23 (a *P. ovale wallikeri* infection) and MSC29 (mixed infection with *P. malariae* and *P. falciparum*). The SLO fractions for these two samples underwent minimal lysis upon incubation with the SLO enzyme, and ~200 µl and 100 µl of the samples respectively were frozen and available for Hi-C. We used the Arima2 kit and sequenced these two samples on an Illumina NovaSeq 6000 instrument using 1/8th of a lane, yielding ~22.74 million reads for MSC23 and ~94.72 million reads for MSC29. To provide an estimate of the amount of material present, the volumes of samples used for Hi-C correspond to approximately ~64 ng and ~82 ng of HMW DNA extracted from comparable sample volumes.

Supporting text (S1 Appendix) provides additional details on sample preparation, limitations, and related methodology, based on our experience generating high-quality assemblies directly from natural infections.

#### Heterozygosity of samples.

Genome-wide heterozygosity was estimated from HiFi reads mapped to to a combined reference of GRCh38 (GENCODE v32/Ensembl 98), Pf3D7, PowCR01, and PmUG01 (PlasmoDB v68) using minimap2 (map-hifi preset). Alignments were filtered to retain primary, high-MAPQ, species-assigned reads ((samtools view -F 0x900 -q 60, excluding secondary and supplementary alignments), and a species-specific BAM file was extracted based on chromosome/contig naming: Pf3D7-prefixed contigs for *P. falciparum*, PmUG01-prefixed contigs for *P. malariae*, and LT/FLRJ-prefixed contigs for *P. ovale wallikeri* (PowCR01). Genome-wide heterozygosity was estimated from these species-specific alignments. Variants were then called using bcftools mpileup/call (base quality ≥30, multiallelic model), then filtered to confident biallelic SNPs (DP ≥ 20). Within-host genetic diversity was then assessed using vcfR [1.16.0] in R. Allelic depth (AD) and total depth (DP) were extracted per sample and site, and minor allele frequency (MAF) was calculated as the ratio of the second most-abundant allele count to total depth. Sites with depth ≥20 × were considered adequately covered, and calls with MAF ≥ 0.10 at these sites were classified as mixed (polyclonal). Within-host diversity was calculated as the proportion of adequately covered sites classified as mixed. A depth of ≥5x is considered for heterozygosity estimates of samples pxPlaOval8 and the *P. malariae* component of the mixed infection pxPlaFalc46, due to their low coverage.

### Curation and assembly

Quality filtered and deduplicated PacBio HiFi reads were first mapped to the human reference genome (GRCh38.p14) using minimap2 (v2.27) with the option ‘-ax map-hifi’. Unmapped reads representing possible *Plasmodium* reads were extracted using samtools with the ‘-bf 0x4’ flag. These unmapped reads were assembled into contigs with hifiasm [32] using the ‘-l 0’ option to disable purging. Assembly was done both with and without human reads included, to test whether their presence affected assembly quality. The assemblies were concordant based on the size of the largest contig produced (Supplementary file S1 Table). We therefore chose to use unmapped reads only, which also improved assembly speed. The resulting assemblies were scaffolded with Hi-C data from participants MSC23 and MSC29 using YaHS [33]. MSC23 (*P. ovale wallikeri* infection) was used to scaffold PowML03, 04, 08 and 09, while MSC29 (mixed infection with *P. malariae* and *P. falciparum*) was used to scaffold the *P. malariae* and *P. falciparum* assemblies. Manual curation and checks for contamination were done as described previously [78]. Mitochondrial and apicoplast genomes were assembled using MitoHiFi [34]. Genome assembly completeness was assessed using BUSCO (Benchmarking Universal Single-Copy Orthologs) (Manni et al., 2021) against the plasmodium_odb12 lineage dataset.

### Analysis and visualization

#### Identifying *P. ovale* species.

To check the species identity of the *P. ovale* spp. specimens, we examined single nucleotide differences from the *cox1* gene locus upon mapping the raw reads of the *P. ovale* spp. specimens onto the published *P. ovale curtisi* PocGH01 (https://plasmodb.org/: v68) and *P. ovale wallikeri* Pow222 [28] reference genomes with minimap2 (v2.27) with the option ‘-ax map-hifi’. We confirmed that all *P. ovale* we sequenced were highly genetically similar to *P. ovale wallikeri.*

#### Identification of centromeric coordinates and core genomes.

Centromeric coordinates were inferred based on local GC-content profiles. For *P. ovale wallikeri* and *P. malariae* assemblies, which contain a relatively rich GC core, centromeres were identified by a sharp reduction in GC content within this region. This distinct GC-content drop was not observed visually in *P. falciparum* due to its overall high AT-richness. For *P. falciparum* assemblies, the centromeric regions were therefore defined as genomic intervals where GC content dropped below 5% across two consecutive 1000 bp bins. The boundaries of the core genome for *P. ovale wallikeri* and *P. malariae* assemblies were defined by the outermost position at each chromosomal end of a 1:1 ortholog conserved across all human-infecting *Plasmodium* species. As complementary evidence, gene content and GC content was examined across all these assemblies, and these align with the boundaries so identified.

#### Annotation with companion and annotation transfer.

Companion, which has various annotation tools integrated within (Liftoff, RATT, BRAKER2, AUGUSTUS, GeneMark, Aragorn, Infernal), was used to annotate the complete assemblies, including chrA, chrM and any unlocalised contigs [45–49]. We used the *P. ovale curtisi* PocGH01 annotation for PowML03, 04, 08, 09; the *P. malariae* PmUG01 annotation for PmML03, 04, 07; and the *P. falciparum* Pf3D7 annotation for PfML46, PfML47 and PfML72. Companion was used without using transcript evidence or pseudochrome contiguation, and using default settings (“No, do not use reference protein evidence”, “Yes, perform pseudogene detection”, “Yes, use BRAKER for structural annotation”, “No, use Liftoff to transfer reference gene models”).

In addition to the Companion derived annotations, for comparison of transfer between specimens, Liftoff was used to transfer annotations from PowML03/PmML03/Pf3D7 to the other specimens with “-copies” option to look for extra gene copies in the target genome, and default settings for the rest of the options. This was done to retain the same GeneIDs to enable comparison across these assemblies.

The categories of gene families tabulated in the figures and table comprise genes, both protein-coding and pseudogenes, whose description terms include the following text: fam-m/fam-l - “fam-m protein” or “fam-l protein”; pir - “PIR protein” or “*Plasmodium* vivax Vir protein, putative”; var - “*PfEMP1*” var genes; rifin/stevor - “Subtelomeric Variable Open Reading frame, putative” or “Rifin, putative”; sica - “SICA C-terminal inner membrane domain containing protein, putative” or “SICA_C domain-containing protein”; stp1 - “STP1 protein”; surfin - “surface-associated interspersed protein” or “SURFIN”; etramp - “early transcribed membrane protein” or “Malarial early transcribed membrane protein (ETRAMP), putative”; exp - “*Plasmodium* exported protein, unknown function” or “*Plasmodium* exported protein (hyp*), unknown function”; phist - “*PHIST*”; resa - “Plasmodium RESA N-terminal, putative” or “PRESAN domain-containing protein”; rbp - “reticulocyte binding protein*” genes; conserved - “conserved *Plasmodium* protein, unknown function” or “conserved protein, unknown function” or “conserved *Plasmodium* membrane protein, unknown function”; hypothetical - “hypothetical protein” or “hypothetical protein, conserved.”

In Fig 4 and S3 Fig, the genes are grouped into the displayed gene families based on the description terms including the following text: FAM or fam-m/l - “fam-m protein” or “fam-l protein”; PIR - “PIR protein” or “*Plasmodium* vivax Vir protein, putative”; VAR - “*PfEMP1*” var genes; RIF or RIF/STEVOR - “Subtelomeric Variable Open Reading frame, putative” or “Rifin, putative”; EXP - the following exported proteins, “SICA C-terminal inner membrane domain containing protein, putative”, “SICA_C domain-containing protein”, “STP1 protein”, “early transcribed membrane protein”, “*Plasmodium* exported protein, unknown function”, “*Plasmodium* exported protein (hyp*), unknown function”; “*PHIST*”; “*Plasmodium* RESA N-terminal, putative”, “PRESAN domain-containing protein”, “reticulocyte binding protein*”; CPP - “conserved *Plasmodium* protein, unknown function” or “conserved protein, unknown function” or “conserved *Plasmodium* membrane protein, unknown function”; HYPO - “hypothetical protein”; HYPOC “hypothetical protein, conserved.”

#### Identification of dynamic synteny blocks.

Companion annotations of PowML03 and PmML03, together with PlasmoDB v68 Pf3D7 annotations and Liftoff-derived annotations for the remaining assemblies (PowML04,08,09, PmML04,07, and PfML46,47,72), were used for this analysis. Gene coordinates (chromosome, start, end, gene ID) were extracted for the regions of interest. Syntenic relationships between regions were inferred by identifying genes that retained the same gene identifier between source and Liftoff-target assembly annotations (e.g., annotated by Companion in PowML03, and their corresponding transferred annotations in PowML04). Only single-copy genes (genes that don’t share 100% sequence identity with any other gene) in the source (PowML03, PmML03, Pf3D7), and those that transferred with ≥0.9 coverage and ≥0.8 sequence identity were retained. Blocks of genes were defined as contiguous sets of orthologs maintaining relative order in one chromosome of a sample but relocated in another sample.

Synteny plots were generated using R (4.1.2) with ggplot2, focusing on the blocks of interest flanked by a few surrounding genes.

#### Construction of gene similarity networks.

To examine sequence-level relationships among multigene families, we constructed protein similarity networks using an all-versus-all BLASTP approach. The fasta file of protein sequences of interest was used to build a BLAST protein database, and an all-versus-all BLASTP search was performed with an e-value threshold of 1 × 10^−5^. Pairwise connections (edges) between proteins were retained if they exhibited ≥50% amino-acid identity over ≥100 aligned residues. Self-hits were removed. The resulting edge and node tables were imported into Gephi for network visualization (Fig 3C,D, S4C, D Fig). Layouts were computed using the Fruchterman–Reingold force-directed algorithm to highlight clusters of closely related proteins. Nodes were annotated with gene family identity, chromosomal location, and predicted number of transmembrane (TM) domains, allowing visual comparison of family-specific and structural clustering patterns.

#### Tools for visualization.

Dotplot-based comparative analysis (S2A Fig) was done to visualise collinearity using the web application D-GENIES [35] by aligner minimap2 v2.28 and using the “Many repeats” option for repeatedness. For the dotplot-based comparative analysis of the non-core regions of the assemblies (S5 Fig), paf (Pairwise mApping Format) files were generated using minimap2 with the options “-k15 -w10 -m40 --cs”. The resulting files were filtered to remove alignments with less than 10,000 bp and then visualised using D-GENIES [35].

For gene based syntenic visualization (S7 Fig), the Circlize package in R was used. The chromoMap package was used for chromosomal level visualization of GC density (S1F Fig), coverage (S2D Fig) and telomeric repeat density (S2i Fig), across the chromosomes. Syntenic regions and structural variations between the assemblies were identified using SyRI and visualised using plotsr package [36,62] (Fig 2A,C,E, S1G Fig, S6A Fig). The input sam file for SyRi was generated by minimap2 (“minimap2 -ax asm5 --eqx ref.fa query.fa> input.sam”) and run using default parameters (“syri -c input.sam -r ref.fa -q query.fa -k -F S”).

Protein sequence structures of select genes were obtained through Protter (Fig 3) [79]. The remaining plots were generated in R using ggplot2. Packages and their version used to generate the data, tables and figures are listed below.

#### Tools and versions.

**Table:** 

R Package	Version	R Package	Version	R Package	Version
AnnotationHub	3.2.2	IRanges	2.28.0	RVenn	1.1.0
ape	5.8-1	MetBrewer	0.2.0	scales	1.4.0
BiocGenerics	0.40.0	Nebulosa	1.4.0	stringdist	0.9.15
BiocManager	1.30.22	pafr	0.0.2	stringr	1.5.0
Biostrings	2.62.0	plotly	4.10.3	tibble	3.2.1
BSgenome	1.62.0	plyr	1.8.8	tidygraph	1.2.3
chromoMap	4.1.1	png	0.1-8	tidyr	1.3.0
dplyr	1.1.2	Polychrome	1.5.1	tidyverse	2.0.0
ensembldb	2.18.4	purrr	1.0.2	vcfR	1.14.0
GenomeInfoDb	1.30.1	R.utils	2.12.2	VennDiagram	1.7.3
GenomicRanges	1.46.1	RColorBrewer	1.1-3	viridis	0.6.4
ggalluvial	0.12.5	RColorConesa	1.0.0	viridisLite	0.4.2
ggforce	0.4.1	readr	2.1.4	R	4.1.2
ggplot2	4.0.0	reshape2	1.4.4	**Others**	**Version**
ggraph	2.1.0	gViz	1.38.4	D-Genies	1.5.0
genoPlotR	0.8.11	RIdeogram	0.2.2	SyRI	1.7.0
ideogRam	0.1.1	Rsamtools	2.10.0	PretextView	1.0.5
igraph	1.4.2	rtracklayer	1.54.0	hifiasm	0.16.0

## Supporting information

S1 Fig *Ex vivo* enrichment of Plasmodium parasites directly from participant blood enables generation of high quality genomes using PacBio ULI HiFi long read sequencing and Hi-C. **A,** Sample workflow for enrichment of the parasite fractions directly from intravenous donor blood [31,77]. The enriched fractions used for assembly and participants whose material is used for Hi-C are indicated with a ‘&’ in the table below showing the metadata of study participants. In bold green are the best quality assemblies for each of the species. Species are denoted as follows: Po - *Plasmodium ovale* spp., Pm - *Plasmodium malariae*, Pf - *Plasmodium falciparum*. Sample fractions used for data generation are denoted by MACS, SLO and FT_PP, described in detail in the **Methods** Section: MACS - MACS enriched fraction, SLO - Post-MACS, WBC-depleted blood lysed with Streptolysin-O, FT-PP - Post-MACS, WBC-depleted blood lysed with Saponin. Other notations are indicated as follows: Y - Yes, N - No, M - Male, F - Female. MSC25 was a *P. falciparum/P. ovale* spp. mixed infection sample, with *P. falciparum.* **B,** *cox1* gene locus from each of the *P. ovale* reference genomes sequenced here, compared to published *P. ovale curtisi* (PocGH01, PlasmoDB.v68 and Poc221 [28], and *wallikeri* reference genomes (Pow222 [28], confirm each genome is *P. ovale wallikeri*. Raw long reads of the *P. ovale* spp. specimens from this study and from Poc221 and Pow222 were mapped onto the *P. ovale curtisi* PocGH0 (PlasmoDB.v68) and visualised in IGV. Each vertical bar represents a SNP relative to PocGH01 and those from this study match the alt allele in Pow222. C, Table shows the proportion of primary alignments to each reference (PocGH01 and PowCR01, PlasmoDB.v68 and GRCh38) upon mapping of raw HiFi reads to a combined reference genome. **D-F,** Pretext snapshots of individual chromosomes of (**D**) PowML03, (**E**) PmML03, and (**F**) PfML47. Cross-shaped interaction patterns typically associated with centromeric domains are indicated by circles, which were used to infer centromere coordinates, listed in **S2 Table**, of PowML03 and PmML03. Centromere coordinates of PfML47 were not ascertained this way as the stitch pattern is not apparent. **G,** Isochoric structure of the chromosomes of PowML03 is similar to species with similar GC content distribution, *P. vivax* PAM and *P. cynomolgi* M. Isochoric structure of the chromosomes of PmML03 is similar to species with similar GC content, *P. brasilianum* Bolivian I. *P. brasilianum*, a simian parasite was recently sequenced and assembled from *ex vivo* schizont material using high-fidelity PacBio long reads [43] and is regarded as nearly identical to *P. malariae*. *P. falciparum* doesn’t display a similar isochoric structure but does have high GC content in its telomeric ends (blue regions at the chromosome ends). **H,** SyRI synteny plots showing sequence similarity between the assemblies of PmUG01, PmML03, and *P. brasilianum* (Bolivian I).
(PDF)

S2 Fig Chromosomal level comparison of the assemblies and estimation of read coverages and GC content. **A-C)** Chromosome dotplots of the best curated genome compared to the 14 chromosomes of current published reference genomes for (**A**) *P. ovale wallikeri* (PowML03) against Pow222 [28], (**B**) *P. malariae* (PmML03) against PmUG01 [26] and **(C)** *P. falciparum* (PfML47) against Pf3D7. Chromosomes in PowML03, PmML03 and PfML47 and the assemblies from other specimens of these species are ordered, oriented and named according to their syntenic relationship with Pow22, PmUG01 and Pf3D7, respectively. The alignments are colored by identity values, as indicated in the legend, which is a BLAST-like alignment identity computed from the PAF file by D-GENIES [35]. **D,** Coverage across each chromosome of the curated assembly for *P. ovale wallikeri, P. malariae*, and *P. falciparum*, binned into 10,000 bp regions and coloured according to the ranges displayed in the legend. **E,** Density plot showing coverage along with GC% of 10000 bp bins across all the chromosomes, split by their presence in the core or non-core regions, defined by the outermost position on each chromosome end of a 1:1 ortholog conserved across all human-infecting *Plasmodium* species. Extreme coverage regions are observed in the non-core regions of the chromosomes of PfML47 in particular. The plots show the density contours around the individual data points of each bin. **F,** Boxplots showing coverage across genomes of the curated assemblies of the specimens of *P. ovale wallikeri*, *P. malariae*, and *P. falciparum*, binned into 1000 bp regions and displayed for each chromosome, apicoplast and mitochondrial contigs (in maroon, for chrM). **G,** Table showing the mean and median coverages for the curated assemblies across the nuclear genome, chrM, and chrA, and fold change between them which can indicate the mitochondrial and apicoplast copy number. **H,** Gains in lengths of the chromosomes in the assemblies of *P. ovale wallikeri*, *P. malariae* and *P. falciparum* compared to their existing best references (Pow222, PmUG01 and Pf3D7). **i,** Telomeric repeat regions, inferred using telofinder, across the chromosomes of *P. ovale* PowML03, *P. malariae* PmML03, and *P. falciparum* PfML47.
(PDF)

S3 Fig Summary of Companion annotations of PowML03 and PmML03, along with visualisation of GC content within the gene bodies of multigene families and of “hypothetical proteins” in the PfML47 assembly. **A-C,** Identity and coverage of the transferred Companion annotations in **(A)** PowML03 **(B)** PmML03 and **(C)** PfML47. NC denotes non-Liftoff annotations (done by *ab initio* gene prediction by BRAKER2 and non-coding gene prediction by Aragorn, both tools integrated within Companion), thereby lacking identity or coverage information relative to the source references used for annotations (PocGH01, PmUG01 and Pf3D7 respectively for PowML03, PmML03 and PfML47). **D-F,** Stacked histogram plots displaying GC% in the gene body, exonic, and intronic regions of genes belonging to the multigene families in (**D**) PowML03, (**E**) PmML03, and (**F**) Pf3D7, respectively, show their contribution to the skewed nucleotide ratio in the sub-telomeric regions. **G,** In addition to the gene body, intergenic regions are equally GC low in the sub-telomeric regions compared to the core genome. **H,** Companion annotations of “hypothetical proteins” beyond the *var* genes close to the telomeric ends are displayed across all the chromosomal ends of PfML47, along with an example of the gene distribution on the left arm of chr14. In the right panel, genes on the forward strand are coloured blue and those on the reverse strand, maroon. Genes are grouped into the displayed gene families in **A-F and H** into categories as described in Methods. **I,** Distribution of the width of exons in the “hypothetical proteins” annotated beyond the *var* genes, grouped by the number of exons. **J,** Sequence complexity comparison between amino acid sequences of “hypothetical proteins” and the remaining protein set. Points represent individual sequences plotted by Shannon entropy and k-mer diversity ratio, with lower values indicating greater repetitiveness/lower sequence complexity.
(PDF)

S4 Fig Features of *pir* and *fam-m/l* genes and proteins in PowML03 and PmML03. **A,** Exonic structures of PIRs in PowML03, displaying the most common example on top (black rectangles, with red block indicating transmembrane domain). **B,** Exonic structure of PIRs in PmML03, displaying the most common example on top (black rectangles, with red block indicating transmembrane domain). **C,** Protein similarity network of the *pir* genes in PmML03. Coloured by module, by chromosome ID, by number of transmembrane domains (TM), and by number of exons. **D,** Protein similarity network of the *pir* genes in PmML03 and PowML03. Coloured by module, species and by number of transmembrane domains (TM). Species form distinct, non-overlapping clusters, reflecting the sequence divergence of the pir gene family. **E,** Exonic structure of fam-m, fam-l genes in PmML03, displaying the most common example on top (black rectangles, with red block indicating transmembrane domain).
(PDF)

S5 Fig Self dotplots of the assemblies show shared sequence identity in their subtelomeric regions. **A-C,** Self dot plot of assemblies of PowML03, PmML03 and PfML47, showing shared sequence identity across the subtelomeric regions. **D-E,** Self dot plot of only the non-core regions of PowML03 and PmML03. The dotplot in **(D)** highlights the left arm of chr13 which shares little similarity with the rest of the chromosomes. For all these dotplots, the paf (Pairwise mApping Format) file was generated by minimap2 with the options “-k15 -w10 -m40 --cs”, filtering out alignments less than 10,000 bp and plotted using D-genies [35].
(PDF)

S6 Fig Gene based syntenic across the assemblies of each species. **A,** Annotations from PowML03 were transferred onto the other *P. ovale wallikeri* assemblies using Liftoff [46]. Sankey plots were then used to evaluate gene transfer across specimens. While conserved single-copy orthologs within the core genome transferred reliably (top panel, small fraction of genes from PowML03, in grey, lacking its counterpart in PowML04, etc.), multigene families (bottom panel, ~ 30% of genes from PowML03 lacking its counterpart in PowML04, etc.) in the subtelomeric regions showed poor transfer, likely reflecting the rearrangements and recombination characteristic of these regions. **B,** Same as **(A)** but for *P. malariae*. The relatively worse quality of the PmML07 assembly is reflected in the poor transfer of both conserved and multigene family annotations from PmML03 and PmML04. **C,** Using Liftoff-derived gene models within Companion, annotations from Pow222, PmUG01, and Pf3D7 mapped onto the PowML03, PmML03, and PfML47 assemblies, respectively, reveal clear patterns of conserved genomic regions, as visualised through Circos-based synteny plots. **D and E,** Feature based synteny of the unplaced contigs (grey) from lower quality *P. ovale* (**D**) (Pow222, PowML04, 08, 09) and (**E**) *P. malariae* (PmUG01, PmML04 and PmML07) assemblies was assessed in comparison to the best assembly generated here for each species. This shows that many unplaced contigs in poorer quality assemblies are found in the subtelomeric regions of the higher quality assemblies produced here. In particular, the two largest unplaced contigs in PowML04 and PowML09 are syntenic with the long sub-telomeric region of PowML03 chromosome 13. **F,** AT content of the unplaced contigs in the specimens from this study and references on PlasmoDB (v68). **G-I,** Stacked bar plots show the number of transcripts with sequences per dataset classified as unique singletons (orange), duplicated only within the same isolate (pink), only shared across multiple isolates (green), or present both within and across (purple) for **(G)** *pir* in PowML03,04,08,09, **(H)** *pir* in PmML03,04,07, and **(I)** *fam-m/l* in PmML03,04,07. Counts are shown in the table below for each category and sample, with the values in parentheses indicating the number of unique sequences amongst each category.
(PDF)

S7 Fig Identification of dynamic synteny blocks within *P. ovale wallikeri* and *P. malariae* assemblies. **A-C,** Synteny plots across the assemblies generated in this study for the three Plasmodium species showing synteny within the core genome, even across assembly breaks. For *P. falciparum*, Pf3D7 is included along with the other three specimens PfML46, 47 and 72. **D**, Gene movement blocks identified between *P. ovale wallikeri* assemblies (PowML03 with PowML04, PowML08, and PowML09). **E,** Gene movement blocks between *P. malariae* assemblies (PmML03 with PmML04 and PmML07). **F,** Gene movement blocks between *P. falciparum* Pf3D7 and assemblies PfML46, PfML47 and PfML72. Bar plots show the distribution of gene counts per block, and accompanying pie charts summarise the composition of genes within blocks. The categories of gene families displayed in the table are described in **Methods**.
(PDF)

S8 Fig Examples of three loci in PowML03 identified as gene-block movements. For each example, the top left panel shows the syntenic block of genes, while the top right panel highlights the corresponding regions on the chromosomes using SyRI plots. The lower panels show JBrowse views of the corresponding loci in genomes PowML03 (above) and PowML04 (below). Gene annotations for PowML03 were generated using Companion. For PowML04, gene annotations were transferred from PowML03. **A**, A locus on chromosome 5 of PowML03 (above) shares sequence identity with a locus on chromosome 2 of PowML04 (below), as indicated by the shaded pink band connecting the two regions. The gene content within these regions show perfect concordance, whereas this concordance is lost beyond the boundaries of the syntenic block on either side. Raw reads mapping uniquely to each locus in their respective assemblies are shown alongside the gene annotations, with no evidence of breaks or inconsistencies, indicating that these regions could represent true genomic rearrangements rather than assembly artefacts. **B, C,** two further examples of similar dynamics.
(PDF)

S9 Fig Examples of three loci in PmML03 identified as gene-block movements. **A-C** same as **Fig. S8** but with three examples from *P. malariae.*
(PDF)

S10 Fig Assessment of copy number differences and drug-resistance associated loci. **A,** Genomic region surrounding *msp3* in *P. ovale wallikeri* PowML03, *P. malariae* PmML03, and *P. falciparum* PfML47. PowML03 and PmML03 show substantial expansion of the MSP3 locus, with 14 tandem copies on chromosome 10 in PowML03 and 13 tandem copies on chromosome 6 in PmML03. **B,** Genomic region surrounding *pfg27/25* in PowML03, PmML03, and PfML47, highlighting a marked copy number expansion in PmML03 on chromosome 14 and the single copy on chr04 (PmML3_000151100), in the middle panel. **C,** Phylogenetic analysis of Pfg25/27 protein sequences from the specimens shown shows that the expanded *pfg25/27* locus is phylogenetically distinct from the canonical locus on chr04 (PmML3_000151100), which instead clusters more closely with the single copy orthologs from other species. **D,** Alignment of DHFR sequences shows the triple mutant haplotype N51I/C59R/S108N, which is widely prevalent in Mali. The remainder of the sequence is identical to the 3D7 reference. **E**, Alignment of CRT sequences shows the CVIET mutant haplotype across residues 72–76, along with the A220S, Q271E, I356T and R371I mutations in PfML47 and PfML72. The remainder of the sequence is identical to the 3D7 reference.
(PDF)

S1 Table Mapping and assembly statistics for human read filtering,de novo assembly summarizing the different stages of data processing, heterozygosity estimates, and NCBI scaffold information. (XLSX)

S2 Table Information pertaining to each assembly regarding number of gaps, telomere repeat regions, core genome and centromeric coordinates (file in zenodo repository). (XLSX)

S3 Table Gene summary tables from Companion derived annotations (file in zenodo repository). (XLSX)

S4 Table Gene summary tables from Liftoff derived annotations (file in zenodo repository). (XLSX)

S1 Appendix Additional text detailing sample preparation, limitations, and related methodology. (DOCX)
