# Supplementary material for: High quality chromosomal genome assemblies of three human Plasmodium species directly from natural infections
Source: PLoS Pathog. 2026 Oct 30;22(10):e1014754. doi: 10.1371/journal.ppat.1014754 (PMC13759101; doi:10.1371/journal.ppat.1014754)
Supplement: Supplementary file 1 [file ppat.1014754.s001.pdf]

A

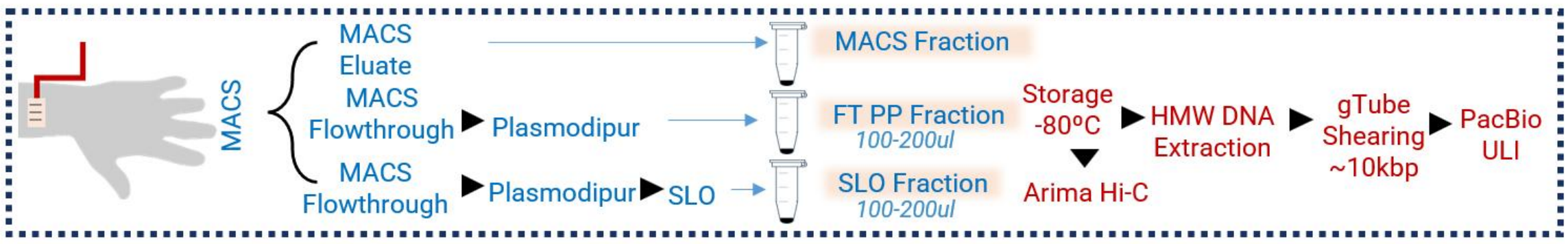

Donor ID	tolID	Assembly Name	Symptom Status	Sex	Age	Weight [kg]	Temp [°C]	Parasitemia	Species by microsopy	Species by sequencing	Sample fraction	Extracted DNA fragment peak	Amount for ULI
MSC23[&]	**pxPlaOval3**	**PowML03**	Sym	F	6	18	37.1	6960	Po	Po	FT_PP	133,000 bp	7.77 ng
MSC32	pxPlaOval4	PowML04	Sym	M	17	56	36.6	17290	Po	Po	MACS	162,000 bp	4.13 ng
MSC31	pxPlaOval8	PowML08	Sym	M	12	36	36.6	995	Po	Po	SLO	77,000 bp	50.6 ng
MSC25	pxPlaOval9	PowML09	Sym	F	6	18	41.2	1840	Po	Po	SLO	73,000 bp	6.67 ng
MSC29[&]	**pxPlaMala3**<br>pxplaFalc72	**PmML03**<br>PfML72	Sym	M	19	52	36.8	1000	Pm	Pf/Pm	FT_PP	146,000 bp	19.23 ng
MSC19	pxPlaMala4	PmML04	Asym	M	8	21	36.4	1200	Pm	Pm	SLO	168,000 bp	2.21 ng
MSC28	pxPlaFalc46<br>pxPlaMala7	PfML46<br>PmML07	Sym	F	8	28	38	3240 Pf,<br>144 Po	Pf/Pm	Pf/Pm	FT_PP	132,000 bp	19.87 ng
MSC61	**pxPlaFalc47**	**PfML47**	Asym	F	10	24	36.1	1440 Pf rings,<br>3008 gams	Pf	Pf	MACS	138,000 bp	11.59 ng

B

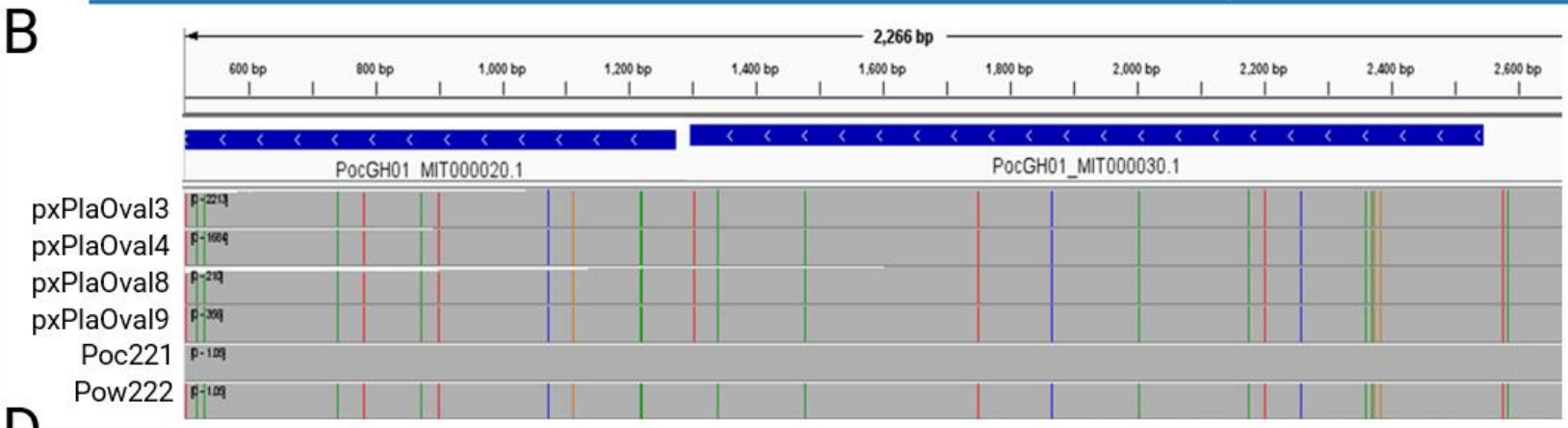

C

pxPlaOval	3	4	8	9
mapped	427063	368120	319222	258915
PowCR01	58.4	27.8	9.4	64.8
PocGH01	3.0	1.5	0.4	4.2
GRCh38	38.6	70.8	90.2	31.0

D E F

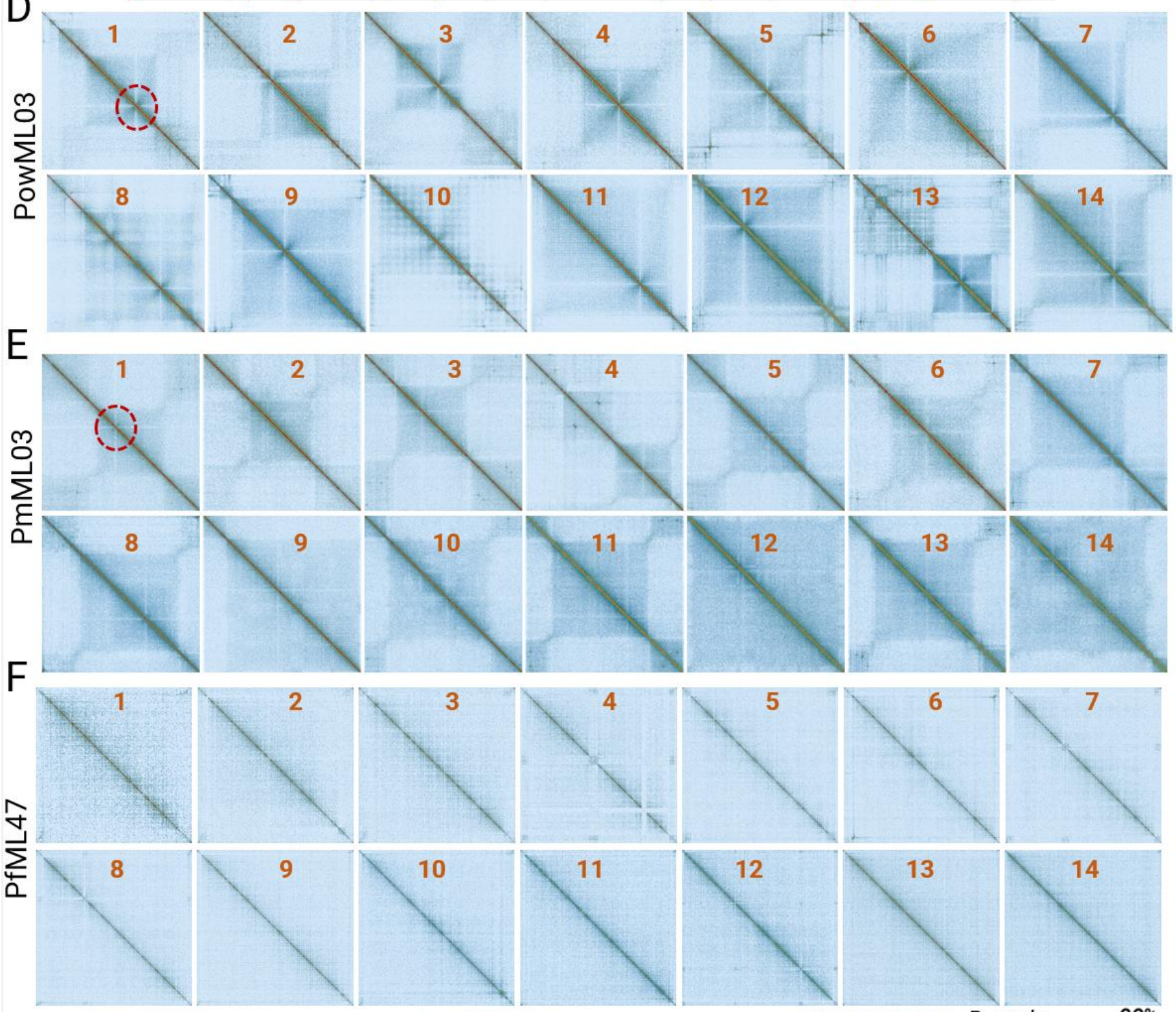

H

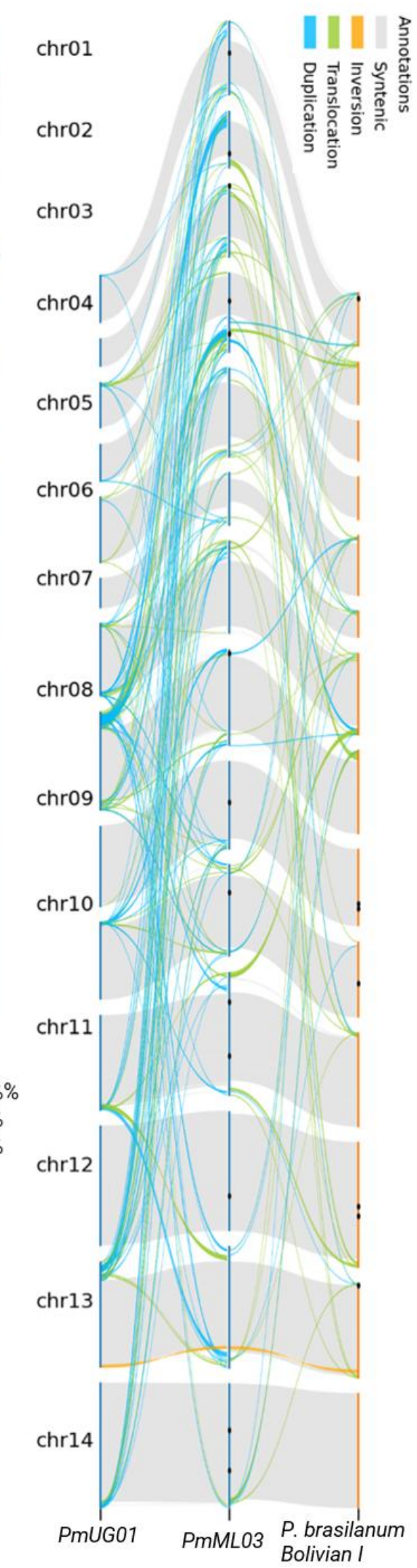

G

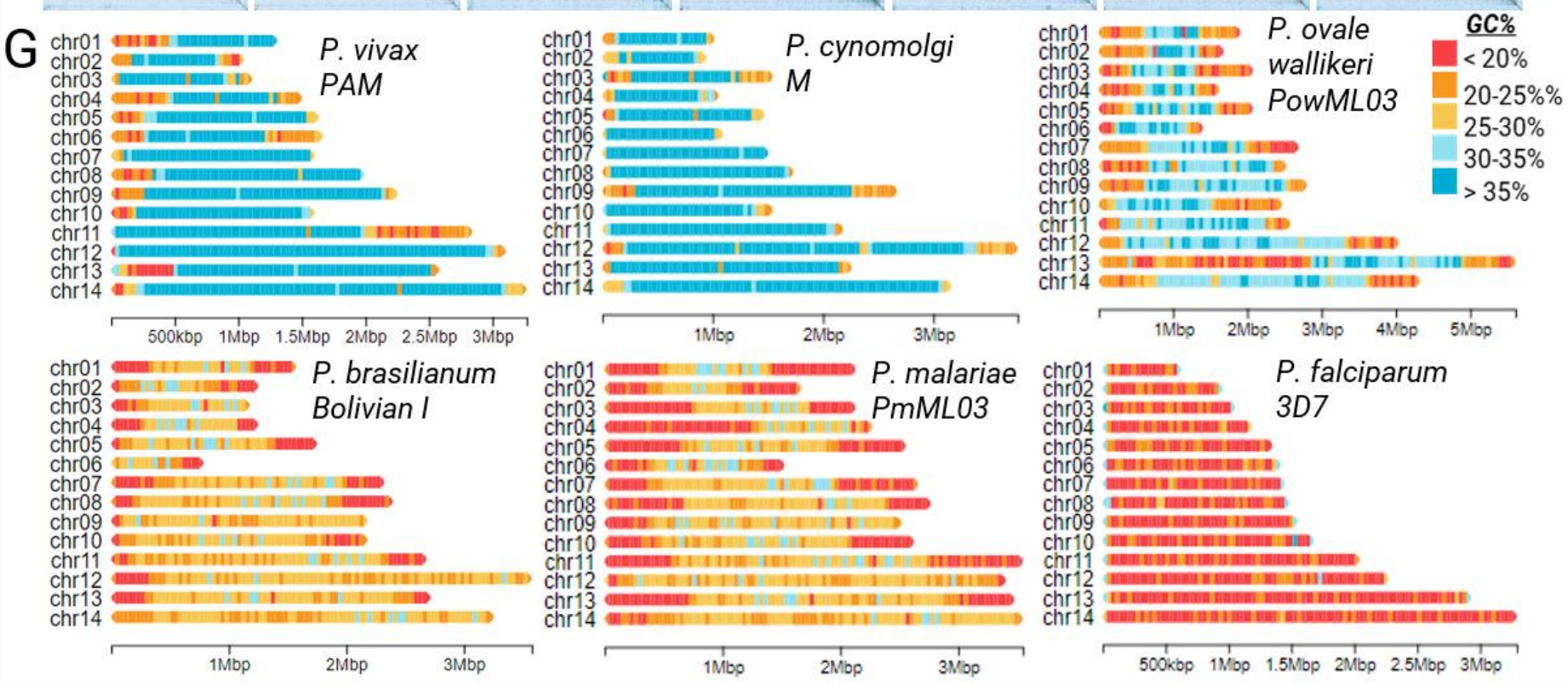

S1 Fig. *Ex vivo* enrichment of Plasmodium parasites directly from participant blood enables generation of high quality genomes using PacBio ULI HiFi long read sequencing and Hi-C. A, Sample workflow for enrichment of the parasite fractions directly from intravenous donor blood [31,77]. The enriched fractions used for assembly and participants whose material is used for Hi-C are indicated with a '&' in the table below showing the metadata of study participants. In bold green are the best quality assemblies for each of the species. Species are denoted as follows: Po - Plasmodium ovale spp., Pm - Plasmodium malariae, Pf - Plasmodium falciparum. Sample fractions used for data generation are denoted by MACS, SLO and FT_PP, described in detail in the Methods Section: MACS - MACS enriched fraction, SLO - Post-MACS, WBC-depleted blood lysed with Streptolysin-O, FT-PP - Post-MACS, WBC-depleted blood lysed with Saponin. Other notations are indicated as follows: Y - Yes, N - No, M - Male, F - Female. MSC25 was a *P. falciparum/P. ovale* spp. mixed infection sample, with *P. falciparum*. **B,** *cox1* gene locus from each of the *P. ovale* reference genomes sequenced here, compared to published *P. ovale curtisi* (PocGH01, PlasmoDB.v68 and Poc221, [28] and *wallikeri* reference genomes (Pow222, [28] confirm each genome is *P. ovale wallikeri*. Raw long reads of the *P. ovale* spp. specimens from this study and from Poc221 and Pow222 were mapped onto the *P. ovale curtisi* PocGH0 (PlasmoDB.v68) and visualised in IGV. Each vertical bar represents a SNP relative to PocGH01 and those from this study match the alt allele in Pow222. **C,** Table shows the proportion of primary alignments to each reference (PocGH01 and PowCR01, PlasmoDB.v68 and GRCh38) upon mapping of raw HiFi reads to a combined reference genome. **D-F,** Pretext snapshots of individual chromosomes of (**D**) PowML03, (**E**) PmML03, and (**F**) PfML47. Cross-shaped interaction patterns typically associated with centromeric domains are indicated by circles, which were used to infer centromere coordinates, listed in **S2 Table**, of PowML03 and PmML03. Centromere coordinates of PfML47 were not ascertained this way as the stitch pattern is not apparent. **G,** Isochoric structure of the chromosomes of PowML03 is similar to species with similar GC content distribution, *P. vivax* PAM and *P. cynomolgi* M. Isochoric structure of the chromosomes of PmML03 is similar to species with similar GC content, *P. brasilianum* Bolivian I. *P. brasilianum*, a simian parasite was recently sequenced and assembled from *ex vivo* schizont material using high-fidelity PacBio long reads [43] and is regarded as nearly identical to *P. malariae*. *P. falciparum* doesn't display a similar isochoric structure but does have high GC content in its telomeric ends (blue regions at the chromosome ends). **H,** SyRI synteny plots showing sequence similarity between the assemblies of PmUG01, PmML03, and *P. brasilianum* (Bolivian I).
