# Supplementary material for: High quality chromosomal genome assemblies of three human Plasmodium species directly from natural infections
Source: PLoS Pathog. 2026 Oct 30;22(10):e1014754. doi: 10.1371/journal.ppat.1014754 (PMC13759101; doi:10.1371/journal.ppat.1014754)
Supplement: Supplementary file 2 [file ppat.1014754.s002.pdf]

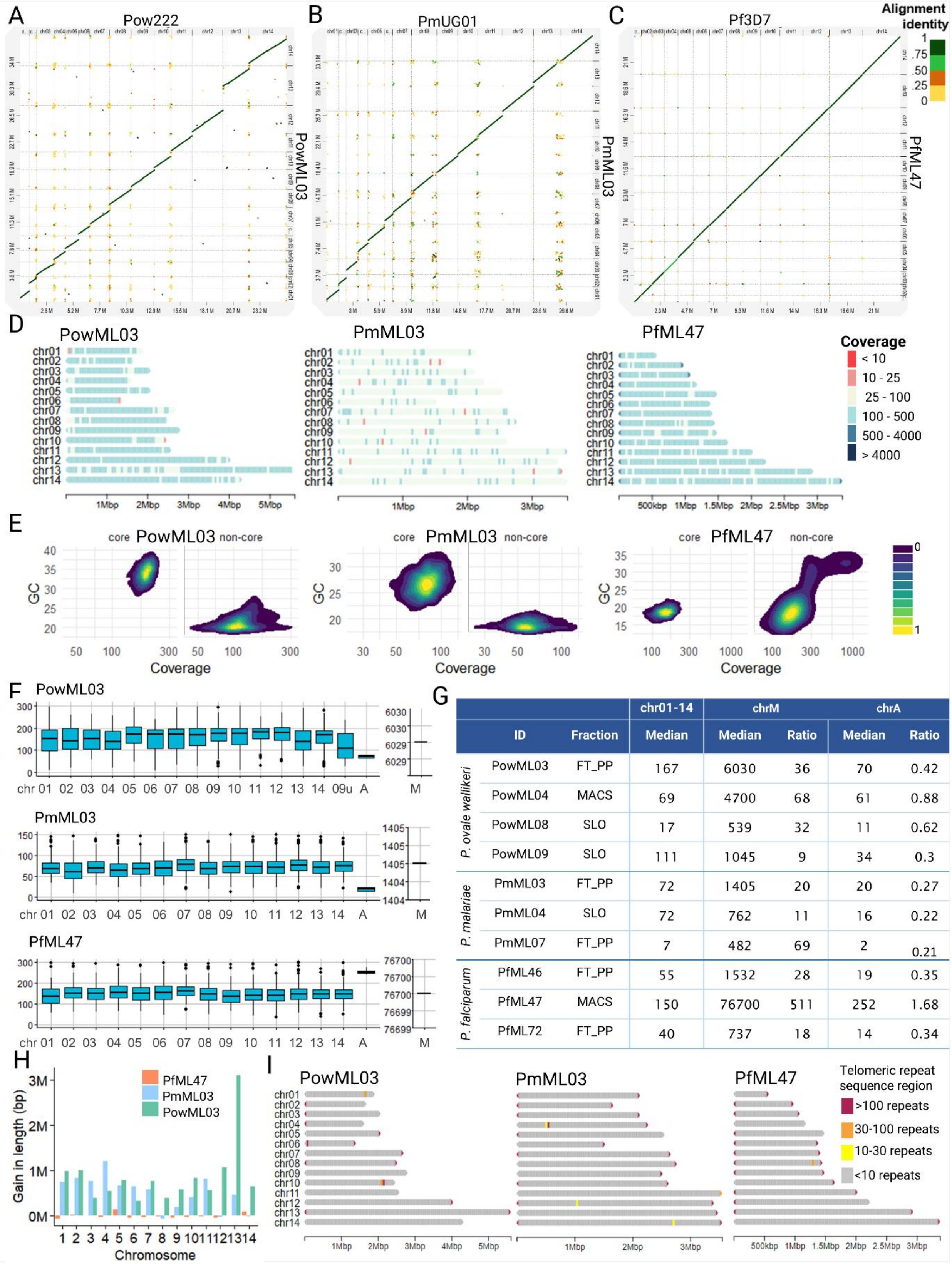

	ID	Fraction	chr01-14 Median	chrM Median	chrM Ratio	chrA Median	chrA Ratio
P. ovale wallikeri	PowML03	FT_PP	167	6030	36	70	0.42
	PowML04	MACS	69	4700	68	61	0.88
	PowML08	SLO	17	539	32	11	0.62
	PowML09	SLO	111	1045	9	34	0.3
P. malariae	PmML03	FT_PP	72	1405	20	20	0.27
	PmML04	SLO	72	762	11	16	0.22
	PmML07	FT_PP	7	482	69	2	0.21
P. falciparum	PfML46	FT_PP	55	1532	28	19	0.35
	PfML47	MACS	150	76700	511	252	1.68
	PfML72	FT_PP	40	737	18	14	0.34

S2 Fig. Chromosomal level comparison of the assemblies and estimation of read coverages and GC content. **A-C)** Chromosome dotplots of the best curated genome compared to the 14 chromosomes of current published reference genomes for (**A**) *P. ovale wallikeri* (PowML03) against Pow222 [28], (**B**) *P. malariae* (PmML03) against PmUG01 [26] and **(C)** *P. falciparum* (PfML47) against Pf3D7. Chromosomes in PowML03, PmML03 and PfML47 and the assemblies from other specimens of these species are ordered, oriented and named according to their syntenic relationship with Pow22, PmUG01 and Pf3D7, respectively. The alignments are colored by identity

values, as indicated in the legend, which is a BLAST-like alignment identity computed from the PAF file by D-GENIES [35]. **D,** Coverage across each chromosome of the curated assembly for *P. ovale wallikeri, P. malariae*, and *P. falciparum*, binned into 10,000bp regions and coloured according to the ranges displayed in the legend. **E,** Density plot showing coverage along with GC% of 10000 bp bins across all the chromosomes, split by their presence in the core or non-core regions, defined by the outermost position on each chromosome end of a 1:1 ortholog conserved across all human-infecting *Plasmodium* species. Extreme coverage regions are observed in the non-core regions of the chromosomes of PfML47 in particular. The plots show the density contours around the individual data points of each bin. **F,** Boxplots showing coverage across genomes of the curated assemblies of the specimens of *P. ovale wallikeri*, *P. malariae*, and *P. falciparum*, binned into 1000bp regions and displayed for each chromosome, apicoplast and mitochondrial contigs (in maroon, for chrM). **G,** Table showing the mean and median coverages for the curated assemblies across the nuclear genome, chrM, and chrA, and fold change between them which can indicate the mitochondrial and apicoplast copy number. **H,** Gains in lengths of the chromosomes in the assemblies of *P. ovale wallikeri*, *P. malariae* and *P. falciparum* compared to their existing best references (Pow222, PmUG01 and Pf3D7). **i,** Telomeric repeat regions, inferred using telofinder, across the chromosomes of *P. ovale* PowML03, *P. malariae* PmML03, and *P. falciparum* PfML47.
