# Supplementary material for: High quality chromosomal genome assemblies of three human Plasmodium species directly from natural infections
Source: PLoS Pathog. 2026 Oct 30;22(10):e1014754. doi: 10.1371/journal.ppat.1014754 (PMC13759101; doi:10.1371/journal.ppat.1014754)
Supplement: Supplementary file 3 [file ppat.1014754.s003.pdf]

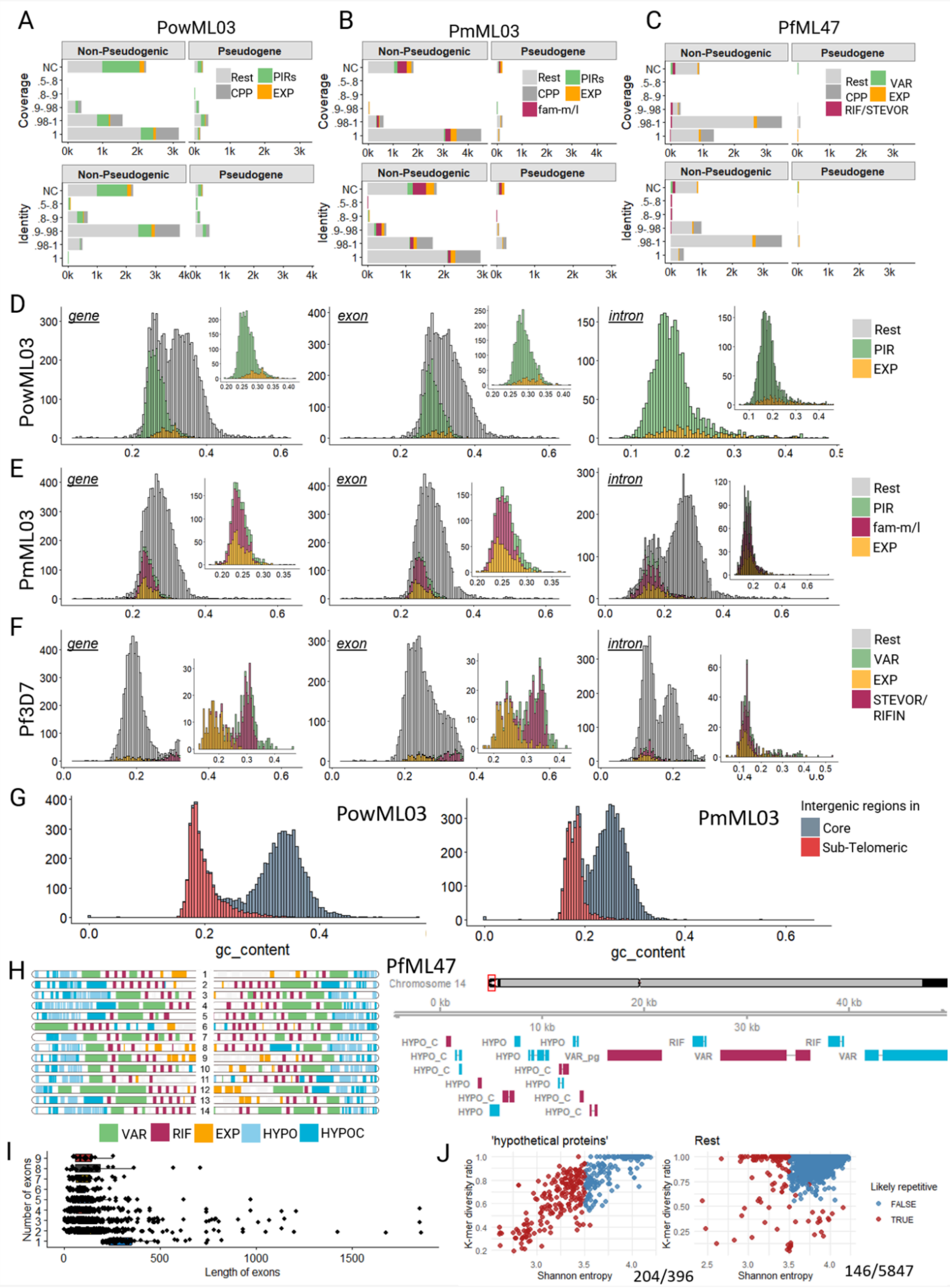

S3 Fig. Summary of Companion annotations of PowML03 and PmML03, along with visualisation of GC content within the gene bodies of multigene families and of "hypothetical proteins" in the PfML47 assembly . A-C, Identity and coverage of the transferred Companion annotations in **(A)** PowML03 **(B)** PmML03 and **(C)**

PfML47. NC denotes non-Liftoff annotations (done by *ab initio* gene prediction by BRAKER2 and non-coding gene prediction by Aragorn), thereby lacking identity or coverage information relative to the source references used for annotations (PocGH01, PmUG01 and Pf3D7 respectively for PowML03, PmML03 and PfML47). **D-F,** Stacked histogram plots displaying GC% in the gene body, exonic, and intronic regions of genes belonging to the multigene families in (**D**) PowML03, (**E**) PmML03, and (**F**) Pf3D7, respectively, show their contribution to the skewed nucleotide ratio in the sub-telomeric regions. **G,** In addition to the gene body, intergenic regions are equally GC low in the sub-telomeric regions compared to the core genome. **H,** Companion annotations of "hypothetical proteins" beyond the *var* genes close to the telomeric ends are displayed across all the chromosomal ends of PfML47, along with an example of the gene distribution on the left arm of chr14. In the right panel, genes on the forward strand are coloured blue and those on the reverse strand, maroon. Genes are grouped into the displayed gene families in **A-F and H** into categories as described in Methods. **I,** Distribution of the width of exons in the "hypothetical proteins" annotated beyond the *var* genes, grouped by the number of exons. **J,** Sequence complexity comparison between amino acid sequences of "hypothetical proteins" and the remaining protein set. Points represent individual sequences plotted by Shannon entropy and k-mer diversity ratio, with lower values indicating greater repetitiveness/lower sequence complexity.
