# Supplementary material for: High quality chromosomal genome assemblies of three human Plasmodium species directly from natural infections
Source: PLoS Pathog. 2026 Oct 30;22(10):e1014754. doi: 10.1371/journal.ppat.1014754 (PMC13759101; doi:10.1371/journal.ppat.1014754)
Supplement: Supplementary file 4 [file ppat.1014754.s004.pdf]

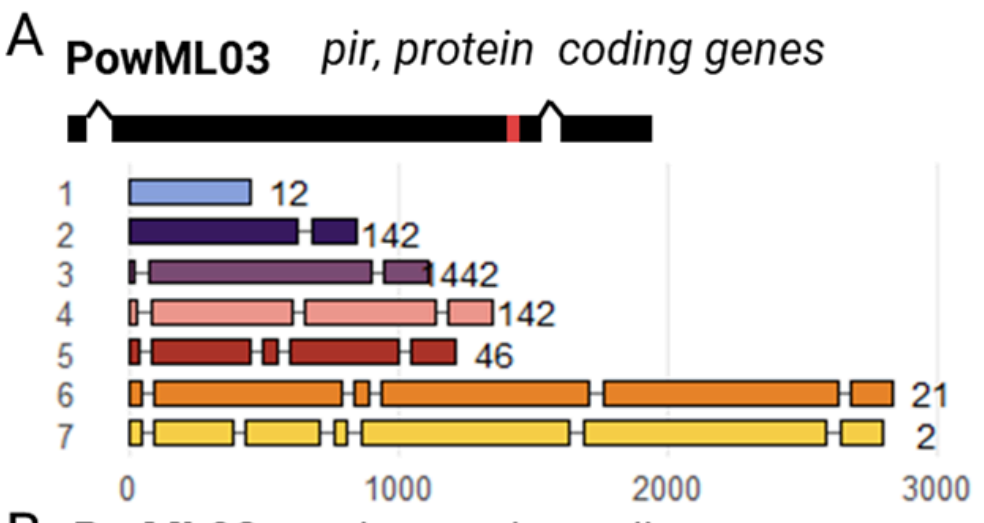

	exons	total	mRNA	mean	median	SP	TMs (0,1,2)	PEXEL canonical relaxed)
	1	38	12	453	455	0	11,1,0	0
	2	216	142	774	787	0	64,75,3	12 (21)
	3	1627	1449	991	1008	2	541,898,3	230 (341)
pir	4	170	142	1270	1094	0	57,84,1	29 (42)
	5	62	46	1217	1320	0	19,20,7	21 (28)
	6	27	21	2376	2589	0	3,17,1	5 (6)
	7	3	2	2501	2501	0	0,2,0	2 (2)

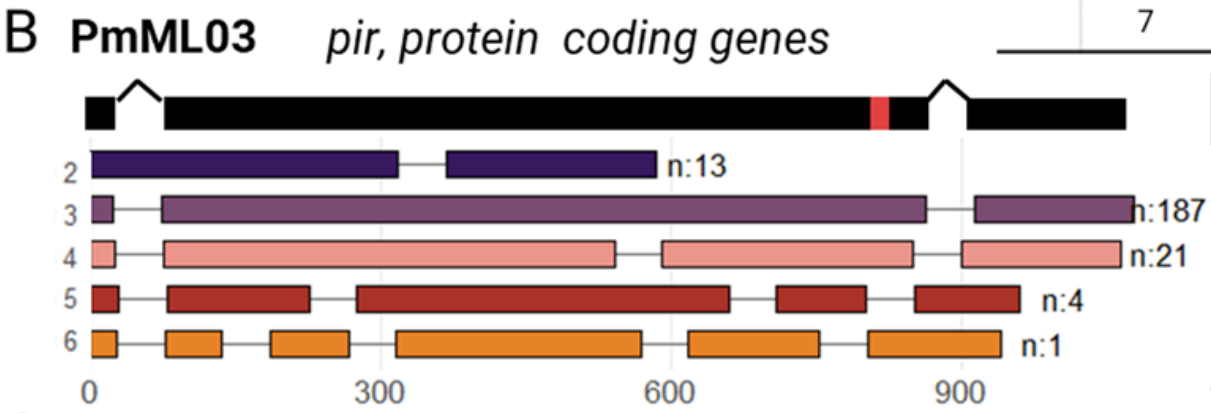

	exons	total	mRNA	median	mean	SP	TMs (0,1,2)	PEXEL canonical (relaxed)
	1	1	--	--	--	--	--	--
	2	21	13	589	577	--	4,7,2	1 (1)
	3	206	187	1156	987	--	27,160,0	59 (75)
pir	4	21	21	1107	1115	--	5,16,0	4 (6)
	5	4	4	870	883	--	1,3,0	0 (0)
	6	1	1	690	690	--	1,0,0	0 (1)

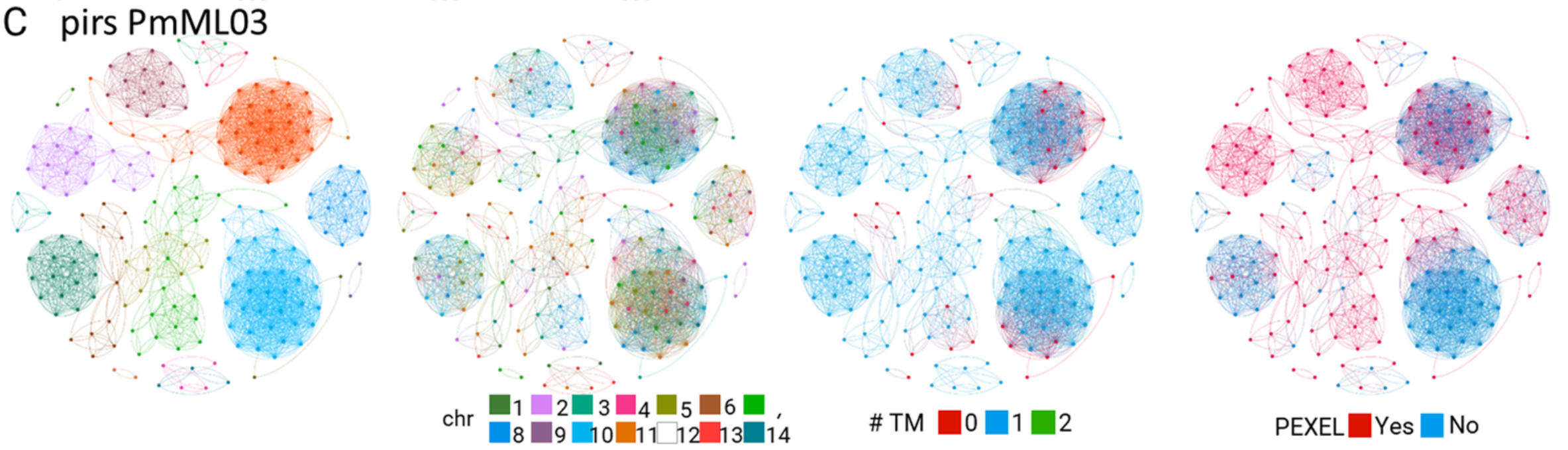

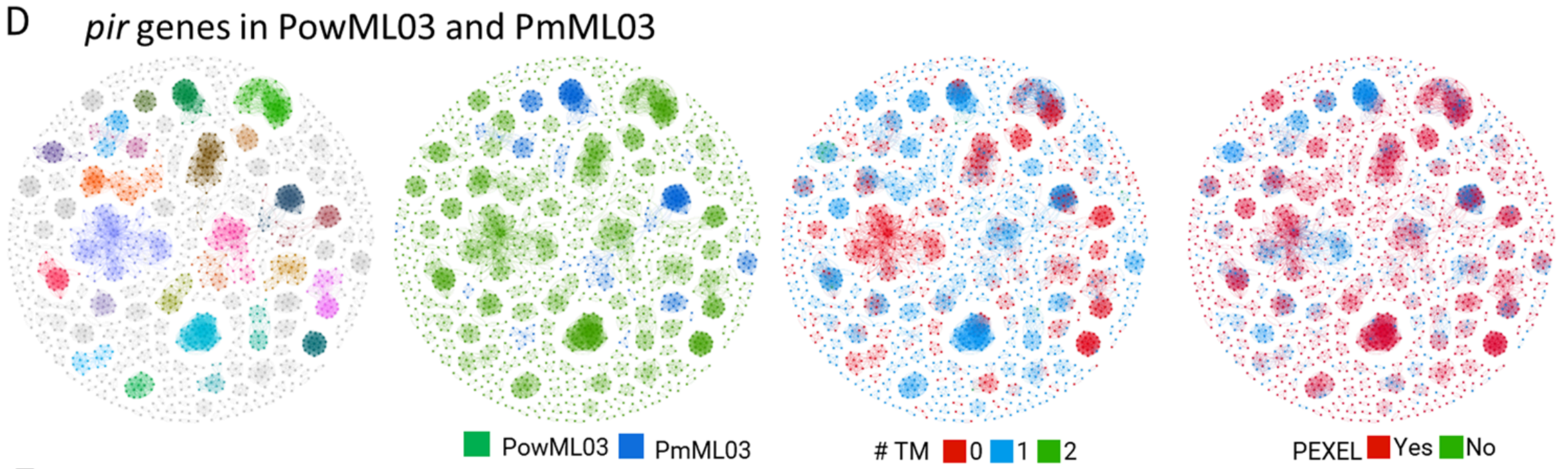

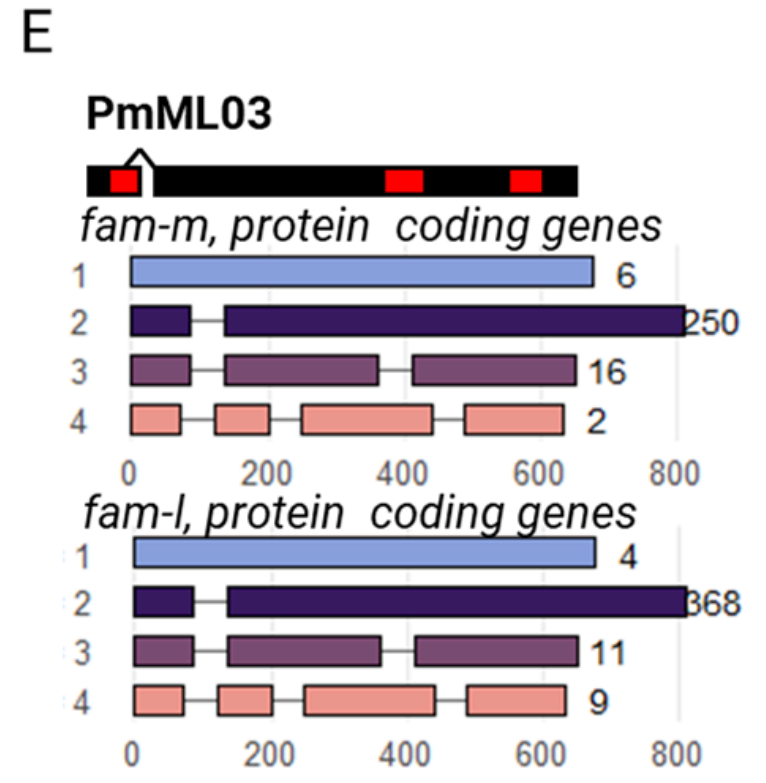

	exons	total	mRNA	mean	median	SP	TMs (0,1,2,3)	PEXEL canonical (relaxed
	1	14	6	590	674	0	0,0,6,0	0 (0)
fam-m	2	270	250	742	760	28	4,15,97,134	16 (24)
	3	28	16	537	596	0	3,3,8,2	0 (0)
	4	3	2	482	482	0	1,0,1,0	0 (0)
	5	1	--	--	--	--	--	--

	exons	total	mRNA	mean	median	SP	TMs (0,1,2,3,4,5)	PEXEL canonical (relaxed
	1	22	4	551	617	1	1,1,2,0,0,0	1 (1)
	2	380	368	848	847	42	1,25,92,233,15,2	45 (62)
fam-l	3	14	11	674	726	0	0,3,4,4,0,0	3 (3)
	4	11	9	574	578	1	2,1,4,2,0,0	0 (0)
	5	3	--	--	--	--	--	--
	6	1	--	--	--	--	--	--

S4 Fig. Features of *pir* and *fam-m/l* genes and proteins in PowML03 and PmML03. A, Exonic structures of PIRs in PowML03, displaying the most common example on top (black rectangles, with red block indicating transmembrane domain). **B,** Exonic structure of PIRs in PmML03, displaying the most common example on top (black rectangles, with red block indicating transmembrane domain). **C,** Protein similarity network of the *pir* genes in PmML03. Coloured by module, by chromosome ID, by number of transmembrane domains (TM), and by number of exons. **D,** Protein similarity network of the *pir* genes in PmML03 and PowML03. Coloured by module, species and by number of transmembrane domains (TM). Species form distinct, non-overlapping

clusters, reflecting the sequence divergence of the pir gene family. **E,** Exonic structure of fam-m, fam-l genes in PmML03, displaying the most common example on top (black rectangles, with red block indicating transmembrane domain).
