# Supplementary material for: High quality chromosomal genome assemblies of three human Plasmodium species directly from natural infections
Source: PLoS Pathog. 2026 Oct 30;22(10):e1014754. doi: 10.1371/journal.ppat.1014754 (PMC13759101; doi:10.1371/journal.ppat.1014754)
Supplement: Supplementary file 5 [file ppat.1014754.s005.pdf]

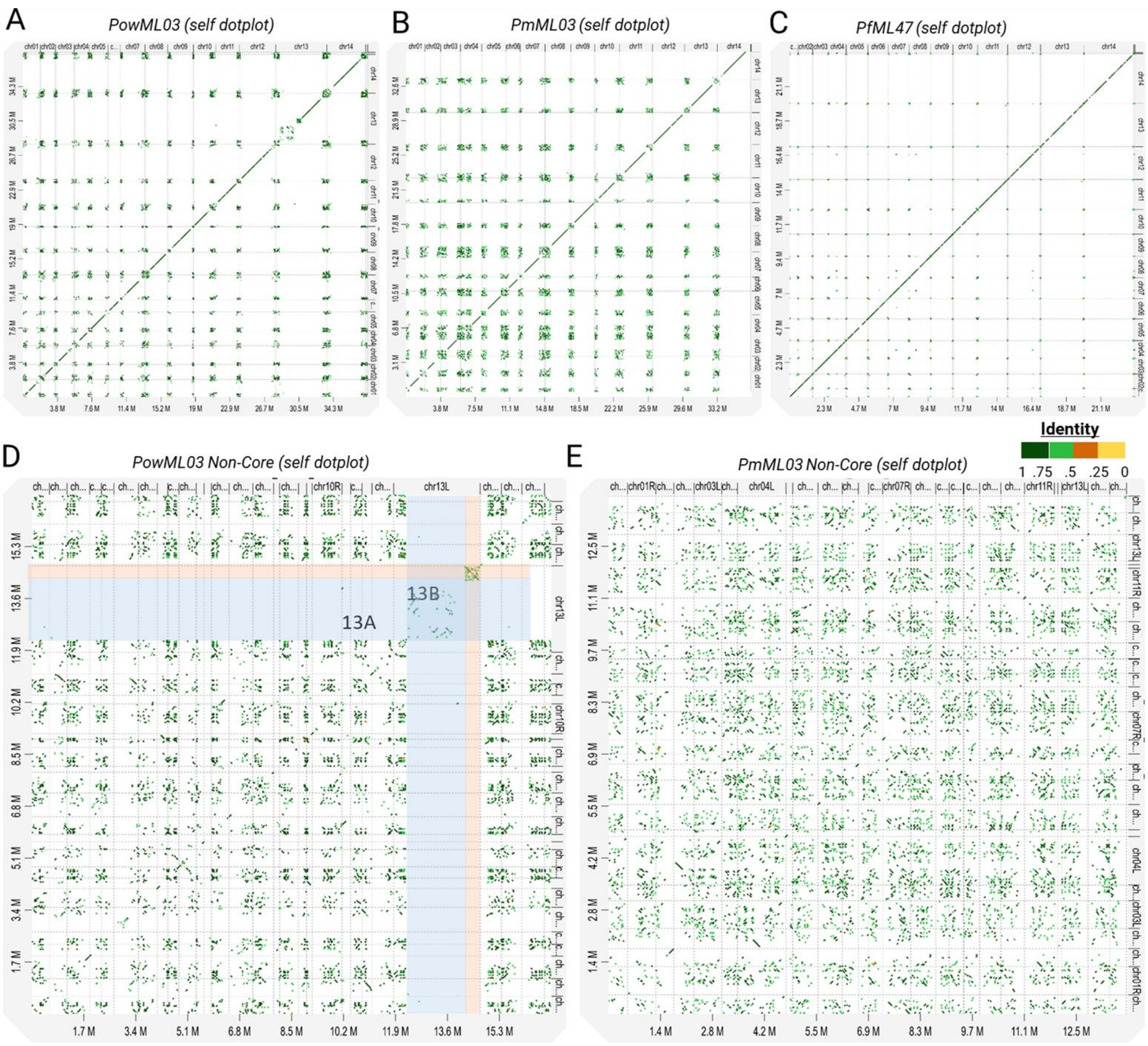

S5 Fig. Self dotplots of the assemblies show shared sequence identity in their subtelomeric regions. A-C, Self dot plot of assemblies of PowML03, PmML03 and PfML47, showing shared sequence identity across the subtelomeric regions. **D-E,** Self dot plot of only the non-core regions of PowML03 and PmML03. The dotplot in **(D)** highlights the left arm of chr13 which shares little similarity with the rest of the chromosomes. For all these dotplots, the paf (Pairwise mApping Format) file was generated by minimap2 with the options "-k15 -w10 -m40 --cs", filtering out alignments less than 10,000 bp and plotted using D-genies [35].
