# Supplementary material for: High quality chromosomal genome assemblies of three human Plasmodium species directly from natural infections
Source: PLoS Pathog. 2026 Oct 30;22(10):e1014754. doi: 10.1371/journal.ppat.1014754 (PMC13759101; doi:10.1371/journal.ppat.1014754)
Supplement: Supplementary file 6 [file ppat.1014754.s006.pdf]

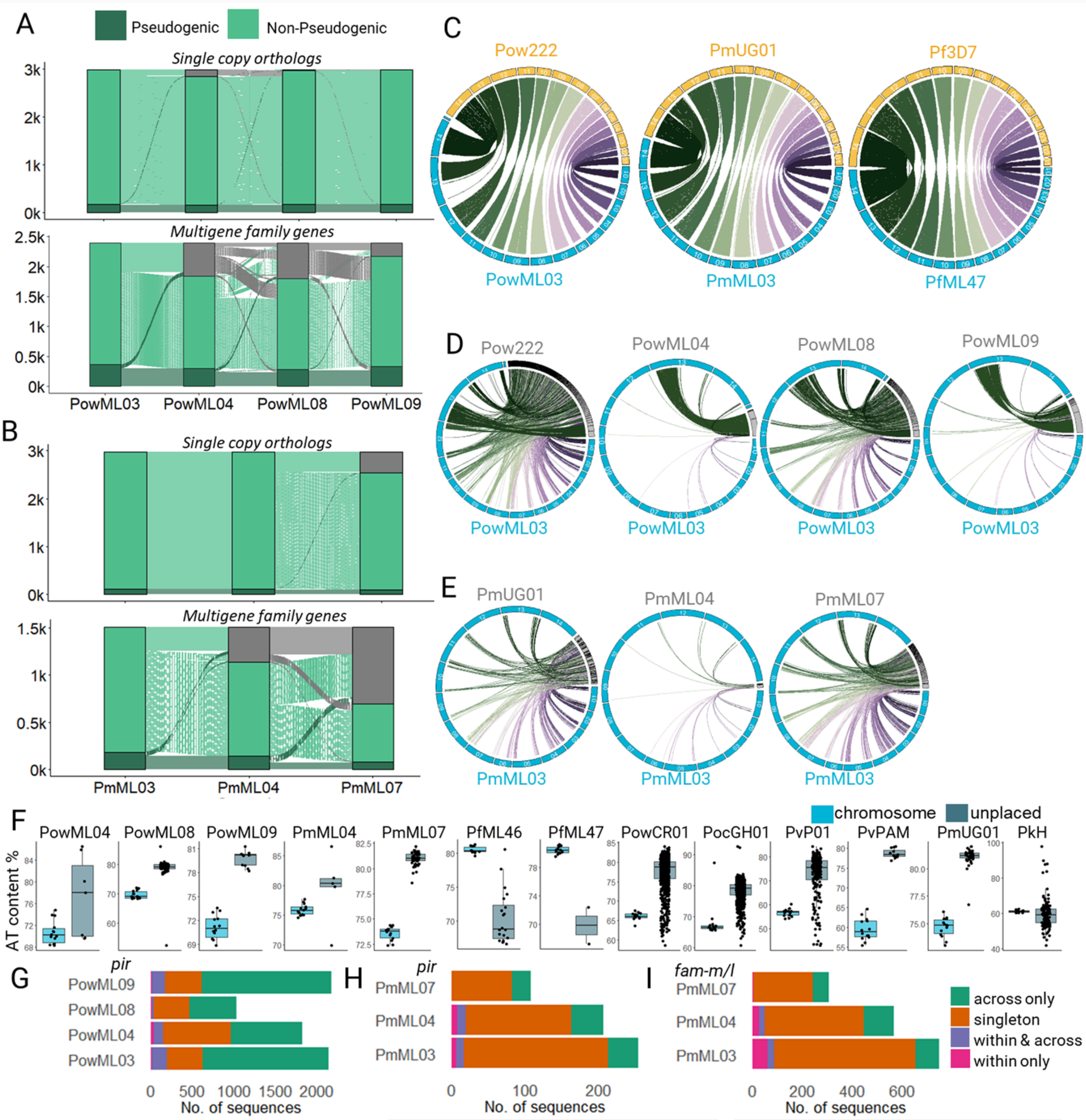

	across only	singleton	within & across	within only	sum
Pow ML03	1520 (1520)	431 (431)	182 (82)	12 (6)	2145 (2039)
Pow ML04	867 (867)	813 (813)	110 (49)	37 (18)	1827 (1747)
Pow ML08	566 (566)	439 (439)	22 (10)	8 (4)	1035 (1019)
Pow ML09	1560 (1560)	441 (441)	158 (70)	14 (7)	2173 (2078)

	across only	singleton	within & across	within only	sum
Pm ML03	41 (41)	196 (196)	11 (5)	6 (3)	254 (245)
Pm ML04	44 (44)	143 (143)	11 (4)	8 (4)	206 (195)
Pm ML07	26 (26)	82 (82)	0 (0)	0 (0)	108 (108)

	across only	single ton	within & across	Within only	sum
Pm ML03	94 (94)	569 (569)	25 (11)	60 (30)	748 (704)
Pm ML04	121 (121)	398 (398)	24 (11)	24 (11)	567 (541)
Pm ML07	66 (66)	236 (236)	2 (1)	2 (1)	306 (304)

S6 Fig. Gene based syntenic across the assemblies of each species. A, Annotations from PowML03 were transferred onto the other *P. ovale wallikeri* assemblies using Liftoff [46]. Sankey plots were then used to evaluate gene transfer across specimens. While conserved single-copy orthologs within the core genome transferred reliably (top panel, small fraction of genes from PowML03, in grey, lacking its counterpart in PowML04, etc.), multigene families (bottom panel, ~30% of genes from PowML03 lacking its counterpart in PowML04, etc.) in the subtelomeric regions showed poor transfer, likely reflecting the rearrangements and recombination characteristic of these regions. **B,** Same as **(A)** but for *P. malariae*. The relatively worse quality of the PmML07 assembly is reflected in the poor transfer of both conserved and multigene family annotations

from PmML03 and PmML04. **C,** Using Liftoff-derived gene models within Companion, annotations from Pow222, PmUG01, and Pf3D7 mapped onto the PowML03, PmML03, and PfML47 assemblies, respectively, reveal clear patterns of conserved genomic regions, as visualised through Circos-based synteny plots. **D and E,** Feature based synteny of the unplaced contigs (grey) from lower quality *P. ovale* (**D**) (Pow222, PowML04, 08, 09) and (**E**) *P. malariae* (PmUG01, PmML04 and PmML07) assemblies was assessed in comparison to the best assembly generated here for each species. This shows that many unplaced contigs in poorer quality assemblies are found in the subtelomeric regions of the higher quality assemblies produced here. In particular, the two largest unplaced contigs in PowML04 and PowML09 are syntenic with the long sub-telomeric region of PowML03 chromosome 13. **F,** AT content of the unplaced contigs in the specimens from this study and references on PlasmoDB (v68). **G-I,** Stacked bar plots show the number of transcripts with sequences per dataset classified as unique singletons (orange), duplicated only within the same isolate (pink), only shared across multiple isolates (green), or present both within and across (purple) for **(G)** *pir* in PowML03,04,08,09, **(H)** *pir* in PmML03,04,07, and **(I)** *fam-m/l* in PmML03,04,07. Counts are shown in the table below for each category and sample, with the values in parentheses indicating the number of unique sequences amongst each category.
