# Supplementary material for: High quality chromosomal genome assemblies of three human Plasmodium species directly from natural infections
Source: PLoS Pathog. 2026 Oct 30;22(10):e1014754. doi: 10.1371/journal.ppat.1014754 (PMC13759101; doi:10.1371/journal.ppat.1014754)
Supplement: Supplementary file 7 [file ppat.1014754.s007.pdf]

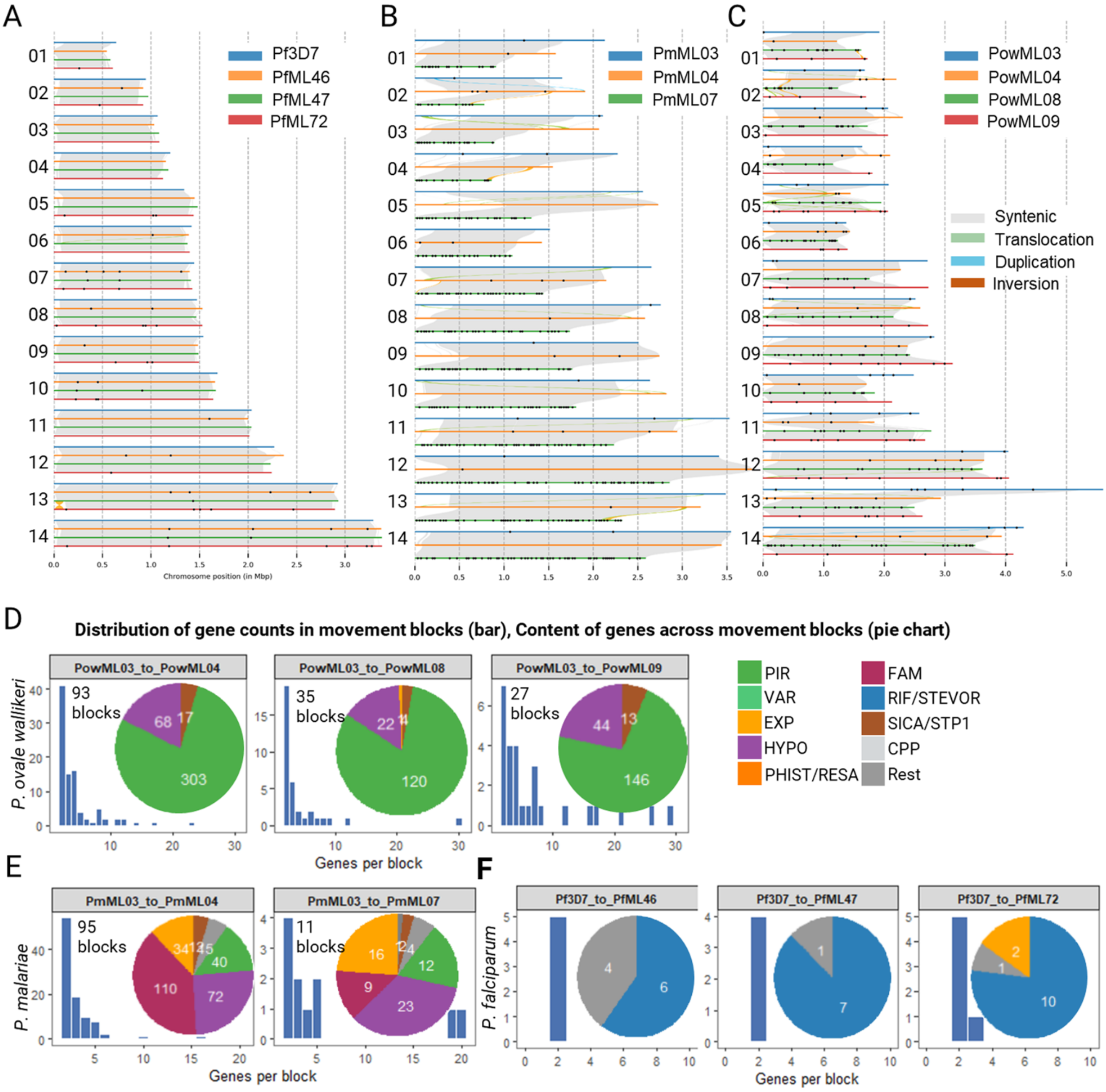

S7 Fig. Identification of dynamic synteny blocks within *P. ovale wallikeri* and *P. malariae* assemblies. A-C, Synteny plots across the assemblies generated in this study for the three Plasmodium species showing synteny within the core genome, even across assembly breaks. For *P. falciparum*, Pf3D7 is included along with the other three specimens PfML46, 47 and 72. **D**, Gene movement blocks identified between *P. ovale wallikeri* assemblies (PowML03 with PowML04, PowML08, and PowML09). **E,** Gene movement blocks between *P. malariae* assemblies (PmML03 with PmML04 and PmML07). **F,** Gene movement blocks between *P. falciparum* Pf3D7 and assemblies PfML46, PfML47 and PfML72. Bar plots show the distribution of gene counts per block, and accompanying pie charts summarise the composition of genes within blocks. The categories of gene families displayed in the table are described in Methods.
