# Supplementary material for: High quality chromosomal genome assemblies of three human Plasmodium species directly from natural infections
Source: PLoS Pathog. 2026 Oct 30;22(10):e1014754. doi: 10.1371/journal.ppat.1014754 (PMC13759101; doi:10.1371/journal.ppat.1014754)
Supplement: Supplementary file 8 [file ppat.1014754.s008.pdf]

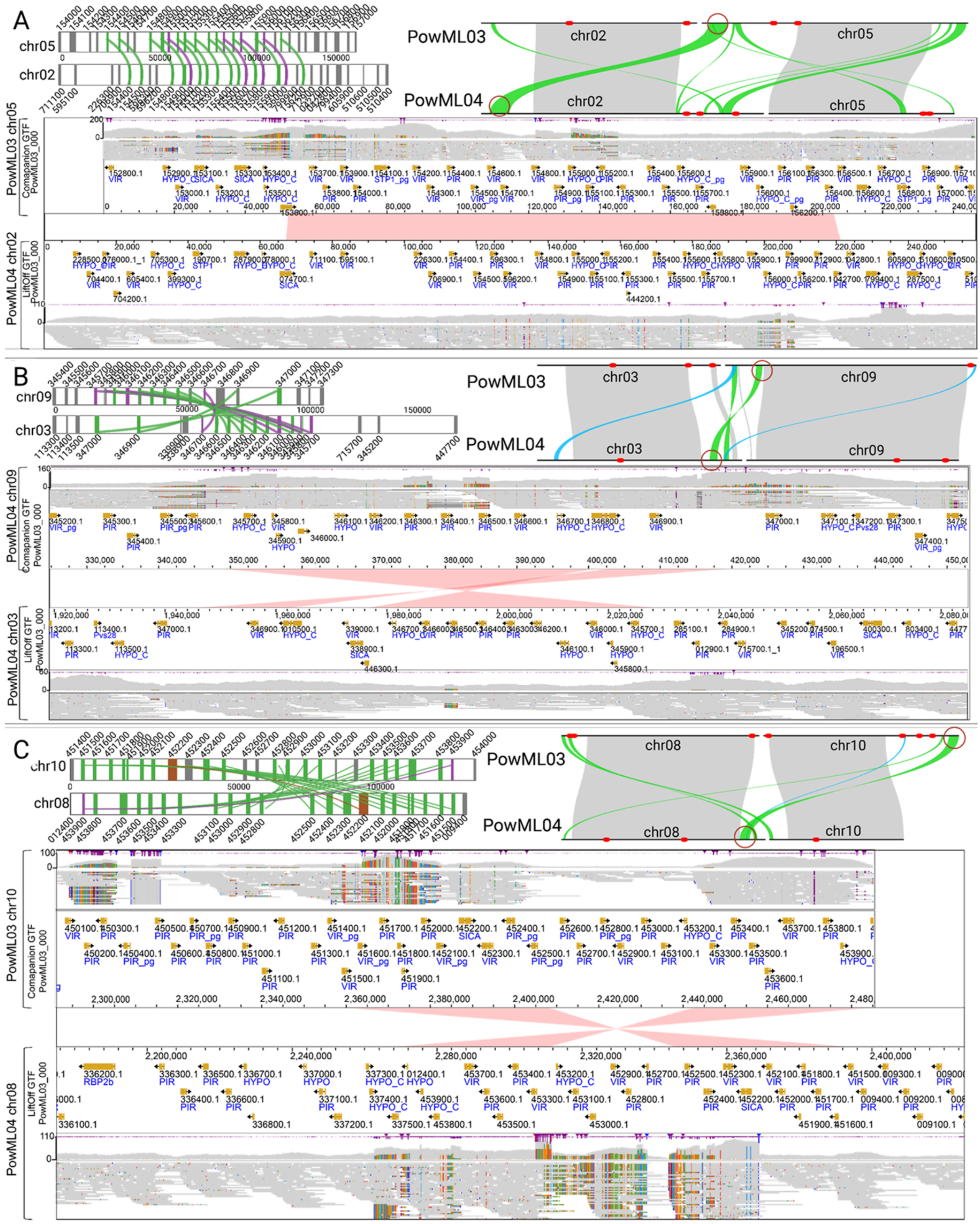

S8 Fig. Examples of three loci in PowML03 identified as gene-block movements. For each example, the top left panel shows the syntenic block of genes, while the top right panel highlights the corresponding regions on the chromosomes using SyRI plots. The lower panels show JBrowse views of the corresponding loci in genomes PowML03 (above) and PowML04 (below). Gene annotations for PowML03 were generated using Companion. For PowML04, gene annotations were transferred from PowML03. **A**, A locus on chromosome 5 of PowML03 (above) shares sequence identity with a locus on chromosome 2 of PowML04 (below), as indicated by the shaded pink band connecting the two regions. The gene content within these

regions show perfect concordance, whereas this concordance is lost beyond the boundaries of the syntenic block on either side. Raw reads mapping uniquely to each locus in their respective assemblies are shown alongside the gene annotations, with no evidence of breaks or inconsistencies, indicating that these regions could represent true genomic rearrangements rather than assembly artefacts. **B, C,** two further examples of similar dynamics.
