# Supplementary material for: High quality chromosomal genome assemblies of three human Plasmodium species directly from natural infections
Source: PLoS Pathog. 2026 Oct 30;22(10):e1014754. doi: 10.1371/journal.ppat.1014754 (PMC13759101; doi:10.1371/journal.ppat.1014754)
Supplement: Supplementary file 9 [file ppat.1014754.s009.pdf]

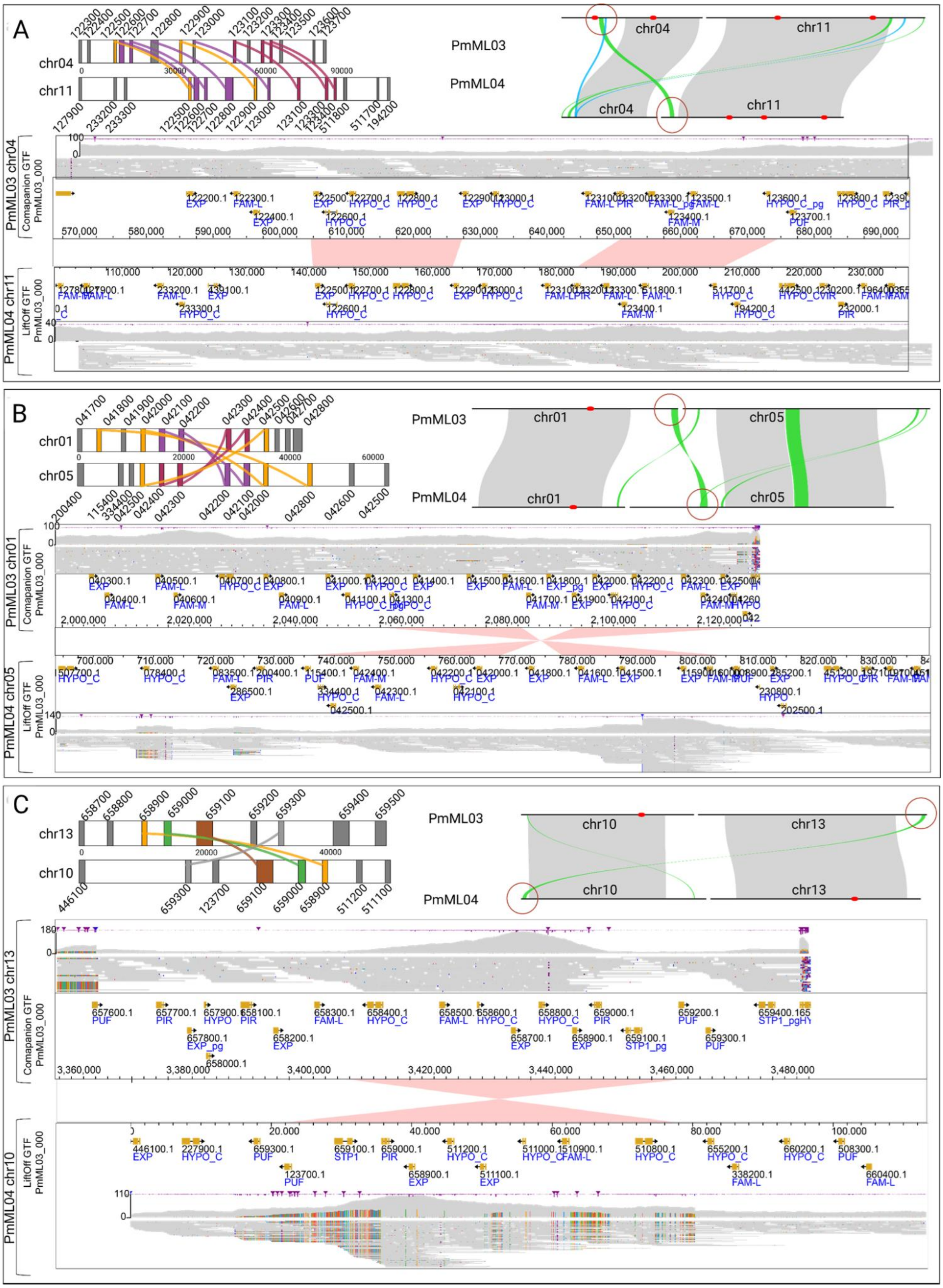

S9 Fig. Examples of three loci in PmML03 identified as gene-block movements. A-C same as **Fig. S8** but with three examples from *P. malariae*
