# Supplementary material for: High quality chromosomal genome assemblies of three human Plasmodium species directly from natural infections
Source: PLoS Pathog. 2026 Oct 30;22(10):e1014754. doi: 10.1371/journal.ppat.1014754 (PMC13759101; doi:10.1371/journal.ppat.1014754)
Supplement: Supplementary file 10 [file ppat.1014754.s010.pdf]

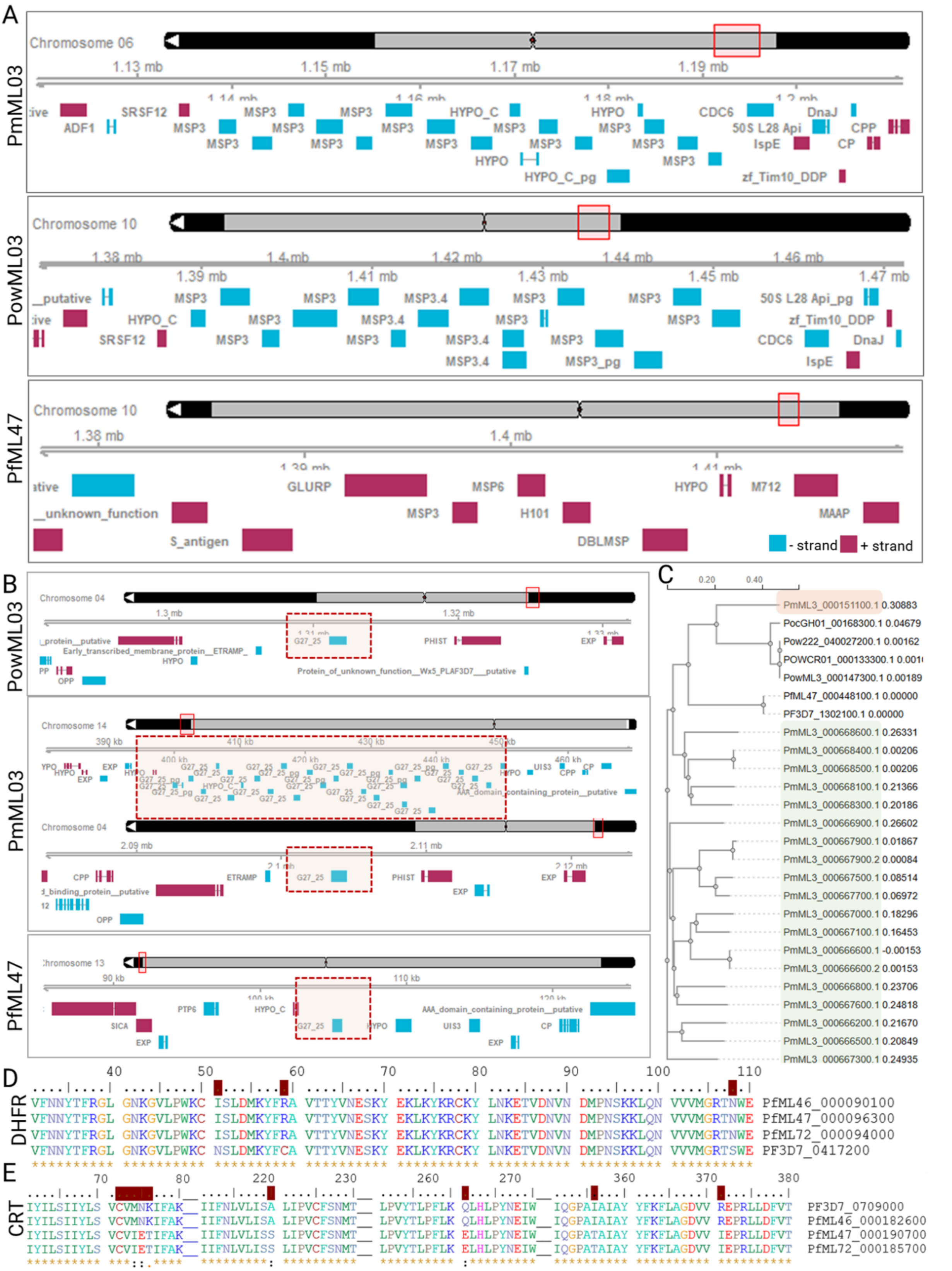

S10 Fig. Assessment of copy number differences and drug-resistance associated loci. A, Genomic region surrounding *msp3* in *P. ovale wallikeri* PowML03, *P. malariae* PmML03, and *P. falciparum* PfML47. PowML03 and PmML03 show substantial expansion of the MSP3 locus, with 14 tandem copies on chromosome 10 in PowML03 and 13 tandem copies on chromosome 6 in PmML03. **B,** Genomic region surrounding *pfg27/25* in PowML03, PmML03, and PfML47, highlighting a marked copy number expansion in PmML03 on chromosome

14 and the single copy on chr04 (PmML3_000151100), in the middle panel. **C,** Phylogenetic analysis of Pfg25/27 protein sequences from the specimens shown shows that the expanded *pfg25/27* locus is phylogenetically distinct from the canonical locus on chr04 (PmML3_000151100), which instead clusters more closely with the single copy orthologs from other species. **D,** Alignment of DHFR sequences shows the triple mutant haplotype N51I/C59R/S108N, which is widely prevalent in Mali. The remainder of the sequence is identical to the 3D7 reference. **E**, Alignment of CRT sequences shows the CVIET mutant haplotype across residues 72-76, along with the A220S, Q271E, I356T and R371I mutations in PfML47 and PfML72. The remainder of the sequence is identical to the 3D7 reference.
