# Supplementary material for: High quality chromosomal genome assemblies of three human Plasmodium species directly from natural infections
Source: PLoS Pathog. 2026 Oct 30;22(10):e1014754. doi: 10.1371/journal.ppat.1014754 (PMC13759101; doi:10.1371/journal.ppat.1014754)
Supplement: Supplementary file 11 [file ppat.1014754.s015.docx]

**S1 Appendix. Supporting text detailing sample preparation, limitations, and related methodology**

**Sample processing and sequencing**

In our study, all parasite enrichment procedures, using magnetic-activated cell sorting (MACS) and/or streptolysin O (SLO)- or Saponin-mediated erythrocyte lysis, were performed on-site in Bamako, Mali. Enriched fractions, frozen at -80°C, were shipped on dry ice to the Wellcome Sanger Institute (WSI), where the samples were stored at -80°C prior to High Molecular Weight (HMW) DNA extraction and long read and Hi-C sequencing. In principle, parasite enrichment and HMW DNA extraction can be performed in any standard molecular laboratory. Access to a long-read sequencer dictates where sequencing can take place

**Sample selection and assembly success**

The sample preparation method utilised for the generation of these assemblies was  optimised for single-cell parasite enrichment. The leftover material used for genome assembly in this study was a secondary application. The MACS fraction was derived from passing 1.5 to 3 mL of whole blood through a MACS column, followed by two on column washes to get rid of uninfected RBCs and WBCs. These fractions were used for scRNA-seq and the left over material from 7 samples (with varying amount of parasite material, yielding a median of 0.243 ng/μl in 100 μl) produced 2 high quality assemblies. The SLO (MACS-flowthrough, WBC-depleted, SLO-lysed) and FT_PP (MACS-flowthrough, WBC-depleted) fractions were derived from only 100 μl of WBC-depleted MACS-flowthrough. These fractions yielded 6 high quality assemblies (out of 15). Four other samples were derived from saponin lysis of the entire leucodepleted MACS flowthroughs (~1 mL). These all had high yield of DNA  (12.6 to 61.4 ng/μl in 100 μl), likely due to the presence of more WBCs in a bigger sample, and failed to generate enough parasite sequencing data.

The proportion of WBCs remaining in the sample was likely the main determinant of outcome, not parasitemia itself. DNA yield gave some early indication of likely success or failure because samples with a higher WBC content (and counterintuitively thus a greater chance of failing to assemble a parasite genome) tend to yield more DNA overall. We successfully obtained genomes from samples with as little as 10% *P. falciparum* material.  However, for future studies focusing on obtaining high quality assemblies, a quick qPCR check could be a more reliable and direct way to assess sample quality before committing to library preparation and sequencing. We had assumed that deeper sequencing could compensate for samples with high human DNA content, but this wasn't the case.

**Protocol optimisation options**

The sample handling protocol could be further optimised to remove more WBCs, for example, through additional washes of the MACS column, or an extra pass of the flow-through fraction through the Plasmodipur filter. Human DNA depletion kits are another option, though these would add to the cost of sample preparation. If not enriching for sexual or asexual fraction using MACS columns, we recommend starting with > 1 mL of whole blood with 2-3 rounds of Plasmodipur filter followed by freezing of 200 μl pellet. Plasmodipur filters were used in this study due to their high efficiency in leukocyte depletion with gentler effect on cells, preserving cell integrity, which was essential for single-cell transcriptomics. However, these filters are relatively expensive (~15–20£ per filter). Alternative, more cost-effective filtration methods, such as CF11, or the new substitute MN2100ff, cellulose columns, may offer a cheaper substitute, though this was not evaluated for this study.

**Sequencing**

For short read resequencing selective whole genome amplification (sWGA) is used to increase the amount of *Plasmodium* specific DNA, especially when extracted from non-leucodepleted blood or dried blood spots. Because the short motifs targeted by sWGA primer sets are unevenly distributed across the target genome, amplification is correspondingly uneven, producing sharp, uneven peaks in coverage. The sequence bias observed in long read genomes generated by sWGA was quite prominent (Oyola et al., 2016, Katlijn De Meulenaere et al mBio 2014). This unevenness is unlikely to compromise variant calling in most genic/core regions, which is important for surveillance applications. However, for applications that depend on even, contiguous coverage, such as generating contiguous *de novo* assemblies or inferring copy-number variants (CNVs) and other structural variants, sWGA fares much worse.

In our case we did not have access to a long read sequencing instrument on site. Sequencing via Oxford Nanopore Technologies (ONT) should be feasible in principle, but would not have worked for the amount of material we had access to. ONT's standard ligation-based kits require a higher amount than PacBio's amplification-based low-input workflows, since they are PCR-free by design and depend on ligation efficiency rather than amplification. It needs inputs more than 100ng and with lower starting material, generally requires either fragmenting the DNA to increase molecule number or amplifying the sample by PCR/sWGA. Parasite-enriched samples utilised in this study were in extremely limited quantities, resulting in correspondingly low DNA yields.

PacBio's AmpliFI option (which replaced Ultra Low Input (ULI) used in our manuscript)  works with as little as 2 ng for a 3 Gb genome. While the ULI and AmpliFI protocols rely on PCR-based amplification, which can introduce coverage bias across GC content, we didn't notice particularly uneven coverage amongst our samples (S2D,E Fig) and obtained assemblies with high contiguity.
