# Supplementary material for: High quality chromosomal genome assemblies of three human Plasmodium species directly from natural infections
Source: PLoS Pathog. 2026 Oct 30;22(10):e1014754. doi: 10.1371/journal.ppat.1014754 (PMC13759101; doi:10.1371/journal.ppat.1014754)
Supplement: Supplementary file 12 [file ppat.1014754.s017.docx]

**Journal Requirements:**

1) Please ensure that the CRediT author contributions listed for every co-author are completed accurately and in full.
 At this stage, the following Authors/Authors require contributions: Jesse Rop, Alex Makunin, Fiona Teltscher, Damon-Lee Pointon, Ying Sims, Marcela Uliano-Silva, James Torrance, Thomas Mathers, Jonathan Wood, Sekou Sissoko, Antoine Dara, Dinkorma Ouologuem, Abdoulaye Djimde, Mara Lawniczak, and Arthur Talman. Please ensure that the full contributions of each author are acknowledged in the "Add/Edit/Remove Authors" section of our submission form.
The list of CRediT author contributions may be found here:

<https://journals.plos.org/plospathogens/s/authorship#loc-author-contributions>

Response: Added full contributions to the submission form

2) Please provide an Author Summary. This should appear in your manuscript between the Abstract (if applicable) and the Introduction, and should be 150-200 words long. The aim should be to make your findings accessible to a wide audience that includes both scientists and non-scientists. Sample summaries can be found on our website under Submission Guidelines:
<https://journals.plos.org/plospathogens/s/submission-guidelines#loc-parts-of-a-submission>

Response: Author summary added to the manuscript.

3) We do not publish any copyright or trademark symbols that usually accompany proprietary names, eg ©, ®, or TM (e.g. next to drug or reagent names). Therefore please remove all instances of trademark/copyright symbols throughout the text, including:
 - TM on page: 35.

Response: Trademark/copyright symbols have been removed

4) Please upload all main figures as separate Figure files in .tif or .eps format. For more information about how to convert and format your figure files please see our guidelines:
<https://journals.plos.org/plospathogens/s/figures>

Response: Main figures provided as .tif files

5) Your current Financial Disclosure states, "Fund is in our acknowledgments section: This work was supported by a Medical Research Council research grant (MR/S02445X/1) to MKNL, 818 which supports SD. This work was also supported by Wellcome core funding to the Wellcome Sanger 819 Institute (Grant 206194/Z/17/Z), which supports MKNL and JCR. Part of the field work in Mali was 820 supported by the BMGF (INV-001927) through RCA No 20_356 between Sanger and USTTB". However, your funding information on the submission form not indicates the Grant 206194/Z/17/Z, INV-001927. Please indicate by return email the full and correct funding information for your study and confirm the order in which funding contributions should appear. Please be sure to indicate whether the funders played any role in the study design, data collection and analysis, decision to publish, or preparation of the manuscript.

Response: The acknowledgements section is correct and reflects the order of the funding. Funders did not play any role in anything other than funding. This will be checked with the submission form.

6) We have amended your Competing Interest statement to comply with journal style. We kindly ask that you double check the statement and let us know if anything is incorrect.

Response: We are happy with the changes.

**Reviewers' Comments:**

 Reviewer's Responses to Questions

**Part I - Summary**
 Please use this section to discuss strengths/weaknesses of study, novelty/significance, general execution and scholarship.

Reviewer #1: The manuscript by Dogga et al. describes the generation of several new genome assemblies for P. ovale wallikeri, P. malariae, and P. falciparum directly from blood samples using long-read DNA sequencing and HiC-based scaffolding. The generation of new genome references for parasites of medical relevance is a significant contribution for science.
 I consider the manuscript provides valuable information that deserves publication. However, there are major issues and flaws that must be addressed first.

Reviewer #2: If I count correctly, this is only the 4th paper to look at clinical genome sequences for P. malariae or P. ovale, and it is the only attempt to use this novel long-read sequencing strategy. These are the two main strengths of this paper. Additionally, prior papers have focused on nucleotide variation, here the authors have focused on protein family evolution and genome structural variation.

 The different approaches (long-read) and a structural focus have uncovered some significant findings of rather extreme structural diversity, rather exclusively in the sub-telomeric regions and involving particular gene families. The analyses of duplication events are particularly nice. I'm genuinely surprised by the levels of circulating sub-telomeric variation observed in isolates from the same region. Did you every observe two very structurally similar isolates? It may be worth adding a sentence or two to the discussion along these lines.

 There are also nice Hi-C findings with respect to centromeres in PowML03 relative to the other species. Quite interesting! This is a well written manuscript that pays particularly nice attention to experimental caveats. The study is well executed and has novel findings.

Reviewer #3: This manuscript presents new genome assemblies for P. malariae, P. ovale wallikeri, and P. falciparum (largely as a positive control) based on ultra-low input PacBio sequencing and HiC sequencing from field samples. This is a useful resource and includes many interesting and well-described analyses, with thoughtful visualizations. It presents (1) a method for recovering high quality assemblies from infected blood without parasite culture, (2) a new resource of reference genomes for P. malariae and P. ovale wallikeri, both of which are substantially more complete than the previous reference and (3) analyses of genome features and hypervariable genes across the three species. All three of these are valuable additions (and could be their own paper!), but the manuscript isn’t consistent about which of these are the focus.

Response: We thank the reviewers for their positive assessment of our manuscript. We addressed the main concerns raised and clarified the text where advised.

The manuscript’s primary contribution is a method to obtain high-quality genome assemblies directly from natural infections without *ex vivo* culturing and to provide data resources for *Plasmodium ovale* and *Plasmodium malariae*. These assemblies in turn allowed us to expand on the scope of the manuscript and look at the highly dynamic sub-telomeric regions, previously unexplored in *P. ovale* and *P. malariae*. We highlight the focus on genome assembly by adding in a new section, an appendix detailing sample preparation, limitations, and related methodology based on some of the queries and suggestions of the reviewers.

To address the above query of Reviewer#2, we didn’t recover any structurally similar isolates amongst our small sample set. It will be interesting to examine with long read data the subtelomeric dynamicity in a more densely sampled dataset and also from longitudinal sampling.

**Part II – Major Issues: Key Experiments Required for Acceptance**
 Please use this section to detail the key new experiments or modifications of existing experiments that should be absolutely required to validate study conclusions.

 Generally, there should be no more than 3 such required experiments or major modifications for a "Major Revision" recommendation. If more than 3 experiments are necessary to validate the study conclusions, then you are encouraged to recommend "Reject".

**Reviewer #1**: I consider the major flaws in this manuscript to lie in DNA sequencing, scaffolding, and annotation.

 First, the authors described that some samples were identified as mixed infections. What species were identified in those cases, and what was the proportion of DNAseq reads for each species? Given the conservation of some of “the core” genome among Plasmodium parasites, is it possible to separate them in silico? I consider some conserved regions across species to be difficult to separate in silico and can introduce chimeric assemblies into the final result. Please explain in detail how this limitation was overcome.

Response: The two mixed infections are indicated in Table 1 for samples MSC28 and MSC29, now marked clearly with a colored box around them. Both were *P. falciparum/P. malariae* co-infections. *P. falciparum* and *P. malariae* are highly diverged from one another at the nucleotide level, even in typically conserved loci such as rRNA genes. The nucleotide-level identity between them is low enough for long reads to be assembled unambiguously and the resulting contigs to be assigned to their species. We did not observe any evidence of species-chimeric contigs. Separation was straightforward given the sequence divergence between these species and we were able to produce two independent high-quality assemblies from each of the two mixed-infection donors, as noted in the manuscript.

Thanks for the suggestion. We added the read proportion upon mapping of raw HiFi reads onto a combined reference (three Plasmodium species + human reference) to S1 Table.

Second, what about polyclonal infections? Did the authors confirm that all sequenced samples correspond to single clones? I consider that the authors should confirm that heterozygosity levels from polyclonal infections do not introduce sequence bias. The gene copy number variability the authors describe across the manuscript, affecting different gene families and pseudogenes, and the statements in lines 195-197 and 367-368 suggest this limitation was not addressed.

Response: We added a statement and a section in the Methods, indicating the heterozygosity of the samples - “Heterozygosity estimates revealed a minor allele fraction below 0.10 across all samples, except for pxPlaOval8 (0.18, assembly PowML08) and P. malariae fraction of the mixed infection sample pxPlaFalc46 (0.37, assembly PmML07) (S1 Table). The estimates for the latter two samples should be interpreted with caution due to their low sequencing depth.”

And the following brief explanation as to how hifiasm deals with polyclonal samples: Regarding the assembly, when multiple genetically distinct clones are present within the sample, hifiasm can, in principle, separate them into distinct contigs rather than merging their variant positions into one sequence. We used the -l 0 option for hifiasm to disable purging which forces the assembler to keep variants. Purging normally removes divergent haplotig sequence that the assembler interprets as low frequency noise or ordinary heterozygosity. However, even with purging disabled, if a clone is present at low relative abundance, the assembler can still interpret it as background error and exclude from the primary assembly graph. In this case, the primary contig output (p_ctg) that is used for scaffolding, reflects only the majority clone’s sequence. If the minority clone’s sequence is distinct enough to survive, the assembler’s internal filtering will report its sequences in the alternate contig file (a_ctg).

If the clones are divergent [github.com/chhylp123/hifiasm/issues/70](http://github.com/chhylp123/hifiasm/issues/70)) hifiasm can fail to recognize the two divergent sequences as alternate haplotypes and instead places both into the primary contig file. Like they are two unrelated regions of the genome, rather than considering one as an alternate haplotye.

We did not observe evidence of alternate haplotypes that remained in the primary contig file (these will be apparent as duplications in the dotplots and in hic contact maps during scaffolding). In PfML47, only 2 unassembled contigs remained that belong to the subtelomeric region. In PmML03, all contigs from the p_ctg were assembled into the 14 chromosomes. In PowML03, only 1 unplaced contig remained, which likely belongs to chr09 based on hic contact but couldn’t be placed accurately.

The complete hifiasm outputs (#_ctg files) for all the assemblies are provided now in the accompanying Zenodo repository for reference.

Third, the authors only generated HiC data for two Plasmodium samples (Malariae and ovale) and used them to scaffold other isolates. Although the authors acknowledge this limitation, I consider it wasn’t properly addressed in the final result. Please provide evidence that this didn’t introduce assembly chimeras. I suggest including HiC coverage per sample, mapping rates, identification and confirmation of structural variants, etc. Why did the authors decide not to use those genomes for which DNAseq and HiC were generated as a reference for scaffolding the others? Instead of using the raw data and potentially introducing incorrect information.
 In addition, the authors used HiC data generated for P. malariae for scaffolding a P. falciparum sample (PfML47). Why did the authors choose malariae as a reference? But more importantly, why did the authors not use the P. falciparum 3D7 assembly as a reference for scaffolding it (PfML47)? In my opinion, that would make more sense than using HiC from a different species.

Response: We'd like to clarify a misunderstanding about the origin of the HiC data. This should address the core of the concern. All HiC scaffolding was performed using HiC reads from the same species being scaffolded, not from a different species. HiC data were generated from two study participants: one with a *P. ovale* mono-infection, and one with a *P. falciparum/P. malariae* (Pf/Pm) mixed infection. From the Pf/Pm donor (MSC29), given the divergence between Pm and Pf, HiC reads from the mixed infection sample successfully scaffolded all the Pm and Pf assemblies separately. PfML47 was therefore scaffolded using Pf-specific HiC reads coming from the donor MSC29 (which resulted in its own assemblies PmML03 and PfML46), not P. malariae HiC data.

From the MSC29-Pf/Pm HiC data:

- Pf reads scaffolded the assemblies PfML46, PfML47, PfML72
- Pm reads scaffolded the assemblies PmML03, PmML04, PmML07

From the MSC23-Pow HiC data:

- Pow reads scaffolded the assemblies PowML03, PowML04, PowML08, and PowML09

Because we have HiC data from a strain coming from the same geographical region, a reference-based approach using 3D7 was unnecessary and we didn't want to impose any further 3D7's structural biases on these genomes. Furthermore, the resulting assembly is highly syntenic with 3D7, as seen in Fig2.

Final assembly stats and the number of contigs resulting from hifiasm, and that further went through YaHS scaffolding and final manual curation are provided in Supplementary file Table S1.

 The genome annotation methodology is confusing, and it’s not clear to me why the authors chose to use a combination of different pipelines and tools. Would it not be better to perform de novo gene calling (for instance with BRAKER3) and then polish the annotation with tools like COMPANION (transferred annotation)?

Response: We used only one tool, COMPANION, to do the annotation, within which the *de novo* gene calling tools (BRAKER/Augustus, Infernal, Aragorn) and gene transfer tools (Liftoff) are integrated.

Liftoff, on its own, was used in cases where we intended to retain the same GeneIDs to enable comparison across assemblies.

We added statements to the Methods to clarify the above.

The methodology for identifying centromeres is equally confusing and not supported. First, why is it needed to identify them de novo? Have the centromere locations not already been described (especially in the Pf 3D7)? G+C peaks and drops along chromosomes have also been reported to be associated with different structural and sequence traits, so authors should provide evidence that the sites identified as bona fide centromere locations are genuine.

*P. falciparum* centromeres lack a universally conserved DNA motif. They are instead marked by an extremely AT-rich sequence region, very apparent in the case of *P. ovale* and *P. malariae*. We therefore used this AT-richness criterion to identify centromere locations in the newer assemblies. This approach successfully reproduced the previously reported centromere positions for Pf3D7, determined by centromeric histone variant PfCENH3 occupancy (Hoeijmakers et al, 2012, DOI: 10.1111/j.1462-5822.2012.01803.x) and Hi-C experiments (Bunnik et al, 2019; doi: 10.1073/pnas.1810815116; centromeric coordinates of Pf3D7 reported in http://[apicomplexan3d.lji.org/](http://apicomplexan3d.lji.org/)). This is presented in Supplementary file Table S2, and a separate sheet just summarising the centromere coordinates is added to the file. Further supporting this, these identified centromeric coordinates when used to anchor the gene annotations shown in Fig. 4D, preserve gene synteny across the chromosome.

| **Chromosome** | **Published coordinates from Bunnik et al, 2019** | **Coordinate inference by AT-richness** |
| --- | --- | --- |
| chr01 | 459,121 | 459,000 |
| chr02 | 448,875 | 449,000 |
| chr03 | 599,045 | 599,000 |
| chr04 | 643,266 | 643,000 |
| chr05 | 456,496 | 456,500 |
| chr06 | 479,811 | 480,000 |
| chr07 | 810,480 | 811,000 |
| chr08 | 300,241 | 300,000 |
| chr09 | 1,243,310 | 1,243,000 |
| chr10 | 945,000 | 945,000 |
| chr11 | 833,106 | 833,000 |
| chr12 | 1,283,920 | 1,284,000 |
| chr13 | 1,169,276 | 1,169,000 |
| chr14 | 1,073,306 | 1,073,000 |

Another major issue is the methodology used to analyze gene families (Figure 3). Authors should support the use of BLAST and arbitrary parameters over pre-existing tools, such as MCL, MMseqs, and CLANS. I consider that using BLAST alone can yield a significant number of false positives.

Response: The pre-existing tools use all-to-all blast for determining sequence similarity and we used stringent cutoffs for sequence length and similarity, to be considered for clustering. We mainly wanted to convey a few things: the uniqueness of a subset of PIR genes, the distinct clustering of fam-m and fam-l protein sequences, presence/lack of any clustering dependent on chromosome location, number of exons, transmembrane domains and PEXEL motifs. We believe that the current approach captures this information, although we agree that there might be some false candidates within the assigned clusters.

Finally, I consider the text needs major restructuring for better reading. The results section is redundant (the same results are described in more than one section), includes material and methods (i.e lines 488-491), and, more importantly, it is a combination of results and discussion. There is no discussion section; the current section named as such only describes the major conclusions.

Response: We agree that lines 488-491 mention the method used to annotate the genomes. We felt it re-iterates what annotation dataset was being used for comparison, but agree that it isn’t necessary and removed as suggested.

We appreciate the reviewer’s comment regarding the depth of the discussion. We’ve therefore added an appendix that expands specifically on one of our primary contributions, the method for generating high-quality assemblies directly from natural infections, and changed the section title to Conclusion. We discuss practical considerations from our experience applying this approach, including sample prep requirements and limitations of the method regarding sensitivity to human DNA presence. We feel that our original discussion was relevant to the scope and context of the manuscript, highlighting some key findings, including the subtelomeric dynamics.

There is no information about the completeness/coverage and annotation of the API and mtDNA genomes.

Response: Thanks for this suggestion. The lengths of the chrA and chrM contigs are in Table 1. We added annotation information for chA and chrM to Table 2, coverage information to S2g Fig and mentioned these in the manuscript text.

**Reviewer #2**: No new experiments suggested, but there are two analysie that would add value.
 1) How does the bioinformatics-based (GC-content) centromere prediction for the P. falciparum clinical isolate compare to experimental determination of the centromere in this species by others? Centromeres can and do move, so I am curious of how a bioinformatics analysis of a patient isolate compares to what was most likely an analysis of a laboratory strain.

Response: Our approach, when applied to Pf3D7 genome, quite accurately reproduced the previously reported centromere positions for Pf3D7 (Hoeijmakers et al, 2012, Bunnik et al, 2019, Centromere coordinates in the table below obtained from http://apicomplexan3d.lji.org/ ). We present this in the Supporting File Table_S2, and a separate sheet just summarising the centromere coordinates is added to the file now. Further supporting this, these identified centromeric coordinates when used to anchor the gene annotations shown in Fig. 4D, preserve gene synteny across the chromosome.

| **Chromosome** | **Published coordinates from Bunnik et al, 2019** | **Coordinate inference by AT-richness** |
| --- | --- | --- |
| chr01 | 459,121 | 459,000 |
| chr02 | 448,875 | 449,000 |
| chr03 | 599,045 | 599,000 |
| chr04 | 643,266 | 643,000 |
| chr05 | 456,496 | 456,500 |
| chr06 | 479,811 | 480,000 |
| chr07 | 810,480 | 811,000 |
| chr08 | 300,241 | 300,000 |
| chr09 | 1,243,310 | 1,243,000 |
| chr10 | 945,000 | 945,000 |
| chr11 | 833,106 | 833,000 |
| chr12 | 1,283,920 | 1,284,000 |
| chr13 | 1,169,276 | 1,169,000 |
| chr14 | 1,073,306 | 1,073,000 |

2) S2 Fig G focus only on the mitochondrial genome and not the apicoplast. Based on S2F, the number of reads mapping to the chrA is variable in the samples, as is the number of reads mapping to chrM. Why is there no analysis or discussion of chrA? Is seems to nicely mirror the relative levels of chrM.

Response: Thank you for this suggestion. Added this to S2g Fig and mentioned in the manuscript.

Reviewer #3: My major comments are related to providing additional information to support these, as described below, or clarify focus of the paper it not all are intended to be addressed.
 1) A new method for recovering high quality assemblies from field-collected samples without culturing. The methods describe in some detail approaches that did not work, and samples that were tried but failed, but this information is limited and not presented in any depth in results or discussion. Given that this is quite novel and useful for other researchers to learn of, there should be more discussion of the recommended approach and interpretation of limitations encountered. For example, it was not clear why samples were processed differently, or given that they were, what the authors conclude and would recommend based on the results found. Is the failure rate of samples inevitable, or could they have been screened better to select those that had higher parasite DNA content? Where was the work performed – was this in Mali or at Sanger? Why was assembly done both with and without human reads (Table S1)? Practical information and a discussion section about the experimental design is warranted given that the ability to build reference is a main novel component of the paper (and in the title).

Response: Thanks a lot for the comment and suggestion. We added an Appendix to the manuscript to provide more information and recommendations based on our experience.

Assembly was done both with and without human reads included, to test whether their presence affected assembly quality. The assemblies were concordant based on the size of the largest contig produced (Supplementary file S1 Table). We therefore chose to use unmapped reads only, which also improved assembly speed. Added this statement to the Methods to clarify the approach.

2) A new resource of reference genomes for P. malariae and P. ovale wallikeri. Will these be integrated into PlasmoDB or available in other places where reference genomes can be accessed? Are the resources developed sufficient that these should replace the current best references? Not that this is something the paper should dictate, but I felt it was not clear or sufficiently discussed what considerations would be and how the references can now be utilized.

Response: The best reported assembly from this study should replace the current references for these two species. We added this suggestion now to the conclusion. The accompanying annotations thereby are a significant improvement. In an ongoing study, we are generating short and long read transcriptomic data for these two species, which can be used to further curate and refine the annotations.

3) Analyses of genome features and hypervariable genes across the three species. These sections present lots of biologically interesting findings for genome evolution and detailed descriptions previously under-characterized gene families. However, it was often not clear which findings were *new* to this analyses vs replication of previous results. Since there is a previous reference, some patterns are already known. And, some of the purpose of the paper is to confirm that the new references are high quality and match previous observations. Perhaps it was the mixing of these two goals that made it challenging to see clearly what the new biological findings of these extended assemblies were. Clarifying this would help to highlight the biggest advances and surprising/new results. For example, the gene exon structures and protein prediction patterns must have been known as well as GC content patterns. The authors could bring this up in the discussion to guide the reader in a comparison of what is consistent with previous reports from the genome and what are the main new findings.

Response: We agree with the reviewer that this wasn’t conveyed well. We added a statement to preface the section describing these gene families While some of the patterns (like the GC content patterns for the multifamily genes in *P. ovale* and *P. malariae*) must have been known, they were not reported in detail.

**Part III – Minor Issues: Editorial and Data Presentation Modifications**
 Please use this section for editorial suggestions as well as relatively minor modifications of existing data that would enhance clarity.

Reviewer #1: Authors should emphasize the number of chromosomes showing telomeres and subtelomers at both ends, as this supports their completeness.

Response: This is added to Table 1 for all the assemblies, and mentioned in the main text when describing the telomeric repeat sequences at the ends of the chromosomes.

Authors should consider including BUSCO results for the new assemblies compared to the current genome references.

Response: Thanks for the suggestion. We added this analysis to the manuscript.

All three *P. falciparum* assemblies reached 99% completeness, and the best *P. ovale* and *P. malariae* assemblies (PowML03 and PmML03) scored 98.1% and 98.5%. The remaining Po and Pm assemblies also scored above 97%, except PmML07 at 81.3% (this was a secondary assembly from a mixed-infection sample).

 Given the arguments above, I consider the authors should temper their conclusions and rephrase the title, as generating chromosome-scale scaffolds does not necessarily imply high-quality genomes.

We believe that based on the representation of telomeric repeats or sub-telomeric regions at the ends of the chromosome, by the BUSCO completeness, and low number of gaps, that these genomes are indeed high-quality genomes.

 Line 139. What is a super scaffold? A long chromosome-like scaffold?

Response: A super scaffold is the refined chromosome-level scaffold put together from YaHS scaffolds using mapping coverage data or Hi-C data, as in the case here, by manual curation using PretextView.

Reviewer #2: Line 164 - I'm confused. If there is Hi-C evidence of contact between chr09u and chr09, as stated, why could the contig not be placed? Please provide a bit more detail, was there a minimum threshold required? Are there any long reads that can lend support to the Hi-C data?

Response: Hi-C interaction signals are able to localise the sequence contig to being part of chromosome 09, however because the sequence is part of the repetitive structure found at each end of the chromosome, the signal is ambiguous as to determining an order or orientation of the components.

Line 176 - add a space to 10kbp, 10 kbp

Response: Edited

Lines 238-242 - I'm confused about how the term clustering is used here. There is physical clustering of gene family members along chromosomes for example and then there is 3D clustering in the cell of regions of chromosomes involved in various activities. As the context of this paragraph is genome architecture, it was confusing to me thing about centromere clustering as there is only one on each chromosome. This section would benefit from a teasing out of the different types of clustering and exactly what is being compared.

Response: We thank the reviewer for pointing this out. We refer to the 3D clustering of chromosomal regions and how it can influence in turn the arraying of genes such as the multigene family and core genes. We edited the paragraph to make it clearer.

Lines 286-288 - I would like a more detailed breakout of the improved annotation by genome. As written it is not clear if pseudogenes and annotation differences were encountered in the organellar genome sequences as well.

Response: The counts of genes in chrA and chrM belonging to different gene types are added now to Table 2.

 Lines 289-290 - Based on the previous sentence it is unclear if these total are nuclear only or if the also include the organellar genomes. This point was not clarified in the methods section either. Please clarify here and in Table 2 including the table legend.

Response: The numbers stated in these lines include organellar genomes as well. We mention this in the first few sentences “*across nuclear, mitochondrial, and apicoplast genomes*“ and in the Methods section “Annotation with companion and annotation transfer“. The counts of genes in chrA and chrM belonging to different gene types is added to Table 2.

 Lines 314-315 - LncRNAs can have small open reading frames. It would be useful to the reader to have more detail on the open reading frame size distribution of the additional loci. As there are many P. falciparum expression data sets available, it should be possible to see, for example in PlasmoDB, if there is existing expression evidence for these new predictions.

Response: Thanks for the suggestion. We added a statement and figure (S3I Fig) showing the size distribution of exons of these “hypothetical proteins” in PfML47. We also added a short statement and figure (S3J Fig) highlighting the repetitive nature of the protein sequences arising from the genes. These “hypothetical proteins” have short exons and show strong signal of repetitive sequence motifs. They likely arise from the repetitive genomic regions of the sub-telomeric regions.

In another study from our team, exploring a genome from natural infection and matching scRNA-seq dataset (https://doi.org/10.64898/2026.07.30.740912), we see very few RNA-seq reads at these loci. While a few reads span mono-exonic candidates, we see no evidence for matching splicing patterns for the Companion-predicted multi-exonic genes.

 Lines 494-495 - You state transcript sequences, but no transcript data are presented, perhaps genes?

Response: Referring to transcripts as in the sequence potentially transcribed. Edited to say genes.

 Line 736 - ref for PlasmoDB

Response: Provided the reference to PlasmoDB

 Line 751 - define chrA and chrM at first use.

Response: Now defined in the section “Processing, sequencing, and curation of genome assemblies from natural malaria infections”

 Lines 762-773 - You list the terms that were included but don't clarify is pseudogenes with these terms were excluded or not. Please clarify.

Response: Yes, these category names are for genes with these descriptions, both protein coding and pseudogenes, as inferred by Companion. For example, for Table 2, the counts for both protein coding and pseudogenes is displayed in parentheses, as mentioned in the caption “Gene families’ counts in brackets indicate (coding genes, pseudogenes)”. Will make it clearer within the section mentioned above Lines 762-773

 Lines 802-803 - These parameters are quite lenient (much more so than for ortholog determination for example) - just to clarify, the All vs All was conducted only within families of previously determined orthologs?

Response: The all vs all blastp was performed genome-wide across all protein-coding pirs or fams. We used a lenient threshold with e 10^-5 but restricted it to hits with >50 identity over >100 aligned residues, since we expected them to be highly diverged and rapidly evolving. We were concerned that stricter thresholds would exclude a lot of hits. Our main interest with this analysis was to capture broader patterns like the uniqueness of a subset of pir genes, distinct fam-m/l clustering, and whether clustering was associated with chromosome location, exon number, transmembrane domains, or PEXEL motifs. We believe that the current approach captures some of these patterns nicely, though we agree that some pairwise connections within the assigned clusters may be erroneous or represent false positives.

 Line 819 - Are there parameters for how SyRI was run?

Response: SyRi was run on the *sam* file generated by minimap2. This is now added to the Methods.

 Line 1093 - S2 Fig. what do the colors indicate? a key is not provided.

Response: Legend added to the plot and mentioned in the figure caption

 Figure 1 - Please, for each species have three separate panels is labels, i.e. P. ovale A, B and C then P. malariae D, E, F etc so that the different panels can be clearly explained. Also it is not clear why the three chromosomes were chosen for each species, are these orthologous chromosomes that are being compared between the species? as written it is not clear.

Response: Thanks for the suggestion. These are not orthologous chromosomes being compared. The chromosomes on the left panel are arranged by size and a zoomed in snapshot of a region with three chromosomes was presented in the right panel. The zoomed in version was provided to more clearly illustrate the GC content demarcation aligniing with the Hi-C contact map domains. Similarly, individual Hi-C contact maps were provided for each chromosome in Fig S1C-E, since the cross-shaped pattern is not apparent at the resolution of contact maps in Fig. 1

We divided each panel into 3, to clearly explain the plots.

 Figure 2 - as above, please separate into two separate sub-panels for each species, i.e. P. ovale A and B, and then revise the Figure legend to better describe each of the panels.

Response: Edited as suggested. Thanks for the suggestion.

Reviewer #3: Additional comments:
 - How is it determined that infections are monoclonal vs. polyclonal?

Response: We added a statement in the main text and Methods to comment on the clonality of the samples. And a response as to how hifiasm deals with polyclonal samples is given above in response to Review 1’s major Comment 2.

- Did the authors confirm that the rest of the genome (besides cox1) also matches P. ovale wallikeri?

Response: Yes, we did. We now provide the proportion of reads mapping to each species to a combined reference of PowCR01, PocGH01 and GRCh38 in S1c Fig to provide further confirmation.

- What is the likely reason for remaining assembly gaps – is it genome complexity or low coverage?

Response: Most of the assembly gaps coincide with reduced sequencing coverage loci. A fraction of gaps, however, occur in loci with good coverage. These gaps likely arise from underlying genome complexity that prevented hifiasm’s assembly graph, with its strict resolution criteria, from resolving the join.

Minor comments
 - The coverage distribution should be mentioned in the main text (not just in supp fig). It’s hard to interpret what <1% of 10kb bins with < 25 reads means without some idea of overall coverage levels, or what “relatively uniform” means.

Response: This is now added to the main text.

- Keys to columns in supplementary tables would be helpful when it is not straightforward (e.g., “cat2_medium1”)

Response: Key added to the supplementary table

- It seems odd to show the HiC maps multiple times at different resolution within the same figure (and then again in the supplement).

Response: We felt this was necessary to illustrate the different features of the Hi-C contact maps. A zoomed in snapshot of a region with three chromosomes was presented in Fig 1 to clearly illustrate the GC content demarcation aligning with the Hi-C contact map domains, as well as the drop in GC content at the centromeric locations, not very clear in the panels on the left. Similarly, individual Hi-C contact maps were provided for each chromosome in Fig S1C-E, since the cross-shaped patterns are not apparent for all the chromosomes at the resolution of contact maps in Fig. 1
