# Supplementary material for: High quality chromosomal genome assemblies of three human Plasmodium species directly from natural infections
Source: PLoS Pathog. 2026 Oct 30;22(10):e1014754. doi: 10.1371/journal.ppat.1014754 (PMC13759101; doi:10.1371/journal.ppat.1014754)
Supplement: Peer review history

# Author comment: Author response to Decision Letter 0

**DOI:** 10.1371/journal.ppat.1014754.r001

**Transfer Alert**

This paper was transferred from another journal. As a result, its full editorial history (including decision letters, peer reviews and author responses) may not be present.

1 Jun 2026

# Peer review: Decision Letter 0

**DOI:** 10.1371/journal.ppat.1014754.r002

**Authors:** Tracey Lamb, Jacob Tennessen

20 Jul 2026

PPATHOGENS-D-26-00808

High quality chromosomal genome assemblies of three human Plasmodium species directly from natural infections

PLOS Pathogens

Dear Dr. Lawniczak,

Thank you for submitting your manuscript to PLOS Pathogens. After careful consideration, we feel that it has merit but does not fully meet PLOS Pathogens's publication criteria as it currently stands. Therefore, we invite you to submit a revised version of the manuscript that addresses the points raised during the review process.

Please submit your revised manuscript by Sep 18 2026 11:59PM. If you will need more time than this to complete your revisions, please reply to this message or contact the journal office at plospathogens@plos.org. When you're ready to submit your revision, log on to https://www.editorialmanager.com/ppathogens/ and select the 'Submissions Needing Revision' folder to locate your manuscript file.

Please include the following items when submitting your revised manuscript:

* A letter that responds to each point raised by the editor and reviewer(s). You should upload this letter as a separate file labeled 'Response to Reviewers'. This file does not need to include responses to any formatting updates and technical items listed in the 'Journal Requirements' section below.

* A marked-up copy of your manuscript that highlights changes made to the original version. You should upload this as a separate file labeled 'Revised Manuscript with Track Changes'.

* An unmarked version of your revised paper without tracked changes. You should upload this as a separate file labeled 'Manuscript'.

If you would like to make changes to your financial disclosure, competing interests statement, or data availability statement, please make these updates within the submission form at the time of resubmission. Guidelines for resubmitting your figure files are available below the reviewer comments at the end of this letter.

As the corresponding author, your ORCID iD is verified in the submission system and will appear in the published article. PLOS supports the use of ORCID, and we encourage all coauthors to register for an ORCID iD and use it as well. Please encourage your coauthors to verify their ORCID iD within the submission system before final acceptance, as unverified ORCID iDs will not appear in the published article. Only the individual author can complete the verification step; PLOS staff cannot verify ORCID iDs on behalf of authors.

We look forward to receiving your revised manuscript.

Kind regards,

Jacob A Tennessen

Guest Editor

PLOS Pathogens

Tracey Lamb

Section Editor

PLOS Pathogens

Sumita Bhaduri-McIntosh

Editor-in-Chief

PLOS Pathogens

orcid.org/0000-0003-2946-9497

Michael Malim

Editor-in-Chief

PLOS Pathogens

orcid.org/0000-0002-7699-2064

**Additional Editor Comments (if provided):**

**Journal Requirements:**

If the reviewer comments include a recommendation to cite specific previously published works, please review and evaluate these publications to determine whether they are relevant and should be cited. There is no requirement to cite these works unless the editor has indicated otherwise.

1) Please ensure that the CRediT author contributions listed for every co-author are completed accurately and in full.

At this stage, the following Authors/Authors require contributions: Jesse Rop, Alex Makunin, Fiona Teltscher, Damon-Lee Pointon, Ying Sims, Marcela Uliano-Silva, James Torrance, Thomas Mathers, Jonathan Wood, Sekou Sissoko, Antoine Dara, Dinkorma Ouologuem, Abdoulaye Djimde, Mara Lawniczak, and Arthur Talman. Please ensure that the full contributions of each author are acknowledged in the "Add/Edit/Remove Authors" section of our submission form.

The list of CRediT author contributions may be found here: https://journals.plos.org/plospathogens/s/authorship#loc-author-contributions

2) Please provide an Author Summary. This should appear in your manuscript between the Abstract (if applicable) and the Introduction, and should be 150-200 words long. The aim should be to make your findings accessible to a wide audience that includes both scientists and non-scientists. Sample summaries can be found on our website under Submission Guidelines:

https://journals.plos.org/plospathogens/s/submission-guidelines#loc-parts-of-a-submission

3) We do not publish any copyright or trademark symbols that usually accompany proprietary names, eg ©,  ®, or TM  (e.g. next to drug or reagent names). Therefore please remove all instances of trademark/copyright symbols throughout the text, including:

- TM on page: 35.

4) Please upload all main figures as separate Figure files in .tif or .eps format. For more information about how to convert and format your figure files please see our guidelines:

https://journals.plos.org/plospathogens/s/figures

5) Your current Financial Disclosure states, "Fund is in our acknowledgments section: This work was supported by a Medical Research Council research grant (MR/S02445X/1) to MKNL, 818 which supports SD. This work was also supported by Wellcome core funding to the Wellcome Sanger 819 Institute (Grant 206194/Z/17/Z), which supports MKNL and JCR. Part of the field work in Mali was 820 supported by the BMGF (INV-001927) through RCA No 20_356 between Sanger and USTTB". However, your funding information on the submission form not indicates the Grant 206194/Z/17/Z, INV-001927. Please indicate by return email the full and correct funding information for your study and confirm the order in which funding contributions should appear. Please be sure to indicate whether the funders played any role in the study design, data collection and analysis, decision to publish, or preparation of the manuscript.

6) We have amended your Competing Interest statement to comply with journal style. We kindly ask that you double check the statement and let us know if anything is incorrect.

**Reviewers' Comments:**

Reviewer's Responses to Questions

**Part I - Summary**

Please use this section to discuss strengths/weaknesses of study, novelty/significance, general execution and scholarship.

Reviewer #1: The manuscript by Dogga et al. describes the generation of several new genome assemblies for P. ovale wallikeri, P. malariae, and P. falciparum directly from blood samples using long-read DNA sequencing and HiC-based scaffolding. The generation of new genome references for parasites of medical relevance is a significant contribution for science.

I consider the manuscript provides valuable information that deserves publication. However, there are major issues and flaws that must be addressed first.

Reviewer #2: If I count correctly, this is only the 4th paper to look at clinical genome sequences for P. malariae or P. ovale, and it is the only attempt to use this novel long-read sequencing strategy. These are the two main strengths of this paper. Additionally, prior papers have focused on nucleotide variation, here the authors have focused on protein family evolution and genome structural variation.

The different approaches (long-read) and a structural focus have uncovered some significant findings of rather extreme structural diversity, rather exclusively in the sub-telomeric regions and involving particular gene families. The analyses of duplication events are particularly nice. I'm genuinely surprised by the levels of circulating sub-telomeric variation observed in isolates from the same region. Did you every observe two very structurally similar isolates? It may be worth adding a sentence or two to the discussion along these lines.

There are also nice Hi-C findings with respect to centromeres in PowML03 relative to the other species. Quite interesting! This is a well written manuscript that pays particularly nice attention to experimental caveats. The study is well executed and has novel findings.

Reviewer #3: This manuscript presents new genome assemblies for P. malariae, P. ovale wallikeri, and P. falciparum (largely as a positive control) based on ultra-low input PacBio sequencing and HiC sequencing from field samples. This is a useful resource and includes many interesting and well-described analyses, with thoughtful visualizations. It presents (1) a method for recovering high quality assemblies from infected blood without parasite culture, (2) a new resource of reference genomes for P. malariae and P. ovale wallikeri, both of which are substantially more complete than the previous reference and (3) analyses of genome features and hypervariable genes across the three species. All three of these are valuable additions (and could be their own paper!), but the manuscript isn’t consistent about which of these are the focus.

**********

**Part II – Major Issues: Key Experiments Required for Acceptance**

Please use this section to detail the key new experiments or modifications of existing experiments that should be absolutely required to validate study conclusions.

Generally, there should be no more than 3 such required experiments or major modifications for a "Major Revision" recommendation. If more than 3 experiments are necessary to validate the study conclusions, then you are encouraged to recommend "Reject".

Reviewer #1: I consider the major flaws in this manuscript to lie in DNA sequencing, scaffolding, and annotation.

First, the authors described that some samples were identified as mixed infections. What species were identified in those cases, and what was the proportion of DNAseq reads for each species? Given the conservation of some of “the core” genome among Plasmodium parasites, is it possible to separate them in silico? I consider some conserved regions across species to be difficult to separate in silico and can introduce chimeric assemblies into the final result. Please explain in detail how this limitation was overcome.

Second, what about polyclonal infections? Did the authors confirm that all sequenced samples correspond to single clones? I consider that the authors should confirm that heterozygosity levels from polyclonal infections do not introduce sequence bias. The gene copy number variability the authors describe across the manuscript, affecting different gene families and pseudogenes, and the statements in lines 195-197 and 367-368 suggest this limitation was not addressed.

Third, the authors only generated HiC data for two Plasmodium samples (Malariae and ovale) and used them to scaffold other isolates. Although the authors acknowledge this limitation, I consider it wasn’t properly addressed in the final result. Please provide evidence that this didn’t introduce assembly chimeras. I suggest including HiC coverage per sample, mapping rates, identification and confirmation of structural variants, etc. Why did the authors decide not to use those genomes for which DNAseq and HiC were generated as a reference for scaffolding the others? Instead of using the raw data and potentially introducing incorrect information.

In addition, the authors used HiC data generated for P. malariae for scaffolding a P. falciparum sample (PfML47). Why did the authors choose malariae as a reference? But more importantly, why did the authors not use the P. falciparum 3D7 assembly as a reference for scaffolding it (PfML47)? In my opinion, that would make more sense than using HiC from a different species.

The genome annotation methodology is confusing, and it’s not clear to me why the authors chose to use a combination of different pipelines and tools. Would it not be better to perform de novo gene calling (for instance with BRAKER3) and then polish the annotation with tools like COMPANION (transferred annotation)?

The methodology for identifying centromeres is equally confusing and not supported. First, why is it needed to identify them de novo? Have the centromere locations not already been described (especially in the Pf 3D7)? G+C peaks and drops along chromosomes have also been reported to be associated with different structural and sequence traits, so authors should provide evidence that the sites identified as bona fide centromere locations are genuine.

Another major issue is the methodology used to analyze gene families (Figure 3). Authors should support the use of BLAST and arbitrary parameters over pre-existing tools, such as MCL, MMseqs, and CLANS. I consider that using BLAST alone can yield a significant number of false positives.

Finally, I consider the text needs major restructuring for better reading. The results section is redundant (the same results are described in more than one section), includes material and methods (i.e lines 488-491), and, more importantly, it is a combination of results and discussion. There is no discussion section; the current section named as such only describes the major conclusions.

There is no information about the completeness/coverage and annotation of the API and mtDNA genomes.

Reviewer #2: No new experiments suggested, but there are two analysie that would add value.

1) How does the bioinformatics-based (GC-content) centromere prediction for the P. falciparum clinical isolate compare to experimental determination of the centromere in this species by others? Centromeres can and do move, so I am curious of how a bioinformatics analysis of a patient isolate compares to what was most likely an analysis of a laboratory strain.

2) S2 Fig G focus only on the mitochondrial genome and not the apicoplast. Based on S2F, the number of reads mapping to the chrA is variable in the samples, as is the number of reads mapping to chrM. Why is there no analysis or discussion of chrA? Is seems to nicely mirror the relative levels of chrM.

Reviewer #3: My major comments are related to providing additional information to support these, as described below, or clarify focus of the paper it not all are intended to be addressed.

1) A new method for recovering high quality assemblies from field-collected samples without culturing. The methods describe in some detail approaches that did not work, and samples that were tried but failed, but this information is limited and not presented in any depth in results or discussion. Given that this is quite novel and useful for other researchers to learn of, there should be more discussion of the recommended approach and interpretation of limitations encountered. For example, it was not clear why samples were processed differently, or given that they were, what the authors conclude and would recommend based on the results found. Is the failure rate of samples inevitable, or could they have been screened better to select those that had higher parasite DNA content? Where was the work performed – was this in Mali or at Sanger? Why was assembly done both with and without human reads (Table S1)? Practical information and a discussion section about the experimental design is warranted given that the ability to build reference is a main novel component of the paper (and in the title).

2) A new resource of reference genomes for P. malariae and P. ovale wallikeri. Will these be integrated into PlasmoDB or available in other places where reference genomes can be accessed? Are the resources developed sufficient that these should replace the current best references? Not that this is something the paper should dictate, but I felt it was not clear or sufficiently discussed what considerations would be and how the references can now be utilized.

3) Analyses of genome features and hypervariable genes across the three species. These sections present lots of biologically interesting findings for genome evolution and detailed descriptions previously under-characterized gene families. However, it was often not clear which findings were *new* to this analyses vs replication of previous results. Since there is a previous reference, some patterns are already known. And, some of the purpose of the paper is to confirm that the new references are high quality and match previous observations. Perhaps it was the mixing of these two goals that made it challenging to see clearly what the new biological findings of these extended assemblies were. Clarifying this would help to highlight the biggest advances and surprising/new results. For example, the gene exon structures and protein prediction patterns must have been known as well as GC content patterns. The authors could bring this up in the discussion to guide the reader in a comparison of what is consistent with previous reports from the genome and what are the main new findings.

**********

**Part III – Minor Issues: Editorial and Data Presentation Modifications**

Please use this section for editorial suggestions as well as relatively minor modifications of existing data that would enhance clarity.

Reviewer #1: Authors should emphasize the number of chromosomes showing telomeres and subtelomers at both ends, as this supports their completeness.

Authors should consider including BUSCO results for the new assemblies compared to the current genome references.

Given the arguments above, I consider the authors should temper their conclusions and rephrase the title, as generating chromosome-scale scaffolds does not necessarily imply high-quality genomes.

Line 139. What is a super scaffold? A long chromosome-like scaffold?

Reviewer #2: Line 164 - I'm confused. If there is Hi-C evidence of contact between chr09u and chr09, as stated, why could the contig not be placed? Please provide a bit more detail, was there a minimum threshold required? Are there any long reads that can lend support to the Hi-C data?

Line 176 - add a space to 10kbp, 10 kbp

Lines 238-242 - I'm confused about how the term clustering is used here. There is physical clustering of gene family members along chromosomes for example and then there is 3D clustering in the cell of regions of chromosomes involved in various activities. As the context of this paragraph is genome architecture, it was confusing to me thing about centromere clustering as there is only one on each chromosome. This section would benefit from a teasing out of the different types of clustering and exactly what is being compared.

Lines 286-288 - I would like a more detailed breakout of the improved annotation by genome. As written it is not clear if pseudogenes and annotation differences were encountered in the organellar genome sequences as well.

Lines 289-290 - Based on the previous sentence it is unclear if these total are nuclear only or if the also include the organellar genomes. This point was not clarified in the methods section either. Please clarify here and in Table 2 including the table legend.

Lines 314-315 - LncRNAs can have small open reading frames. It would be useful to the reader to have more detail on the open reading frame size distribution of the additional loci. As there are many P. falciparum expression data sets available, it should be possible to see, for example in PlasmoDB, if there is existing expression evidence for these new predictions.

Lines 494-495 - You state transcript sequences, but no transcript data are presented, perhaps genes?

Line 736 - ref for PlasmoDB

Line 751 - define chrA and chrM at first use.

Lines 762-773 - You list the terms that were included but don't clarify is pseudogenes with these terms were excluded or not. Please clarify.

Lines 802-803 - These parameters are quite lenient (much more so than for ortholog determination for example) - just to clarify, the All vs All was conducted only within families of previously determined orthologs?

Line 819 - Are there parameters for how SyRI was run?

Line 1093 - S2 Fig. what do the colors indicate? a key is not provided.

Figure 1 - Please, for each species have three separate panels is labels, i.e. P. ovale A, B and C then P. malariae D, E, F etc so that the different panels can be clearly explained. Also it is not clear why the three chromosomes were chosen for each species, are these orthologous chromosomes that are being compared between the species? as written it is not clear.

Figure 2 - as above, please separate into two separate sub-panels for each species, i.e. P. ovale A and B, and then revise the Figure legend to better describe each of the panels.

Reviewer #3: Additional comments:

- How is it determined that infections are monoclonal vs. polyclonal?

- Did the authors confirm that the rest of the genome (besides cox1) also matches P. ovale wallikeri?

- What is the likely reason for remaining assembly gaps – is it genome complexity or low coverage?

Minor comments

- The coverage distribution should be mentioned in the main text (not just in supp fig). It’s hard to interpret what <1% of 10kb bins with < 25 reads means without some idea of overall coverage levels, or what “relatively uniform” means.

- Keys to columns in supplementary tables would be helpful when it is not straightforward (e.g., “cat2_medium1”)

- It seems odd to show the HiC maps multiple times at different resolution within the same figure (and then again in the supplement).

**********

PLOS authors have the option to publish the peer review history of their article (what does this mean?). If published, this will include your full peer review and any attached files.

If you choose “no”, your identity will remain anonymous but your review may still be made public.

**Do you want your identity to be public for this peer review?**  For information about this choice, including consent withdrawal, please see our Privacy Policy.

Reviewer #1: No

Reviewer #2: No

Reviewer #3: No

[NOTE: If reviewer comments were submitted as an attachment file, they will be attached to this email and accessible via the submission site. Please log into your account, locate the manuscript record, and check for the action link "View Attachments". If this link does not appear, there are no attachment files.]

**Figure resubmission:**

**Reproducibility:**

To enhance the reproducibility of your results, we recommend that authors of applicable studies deposit laboratory protocols in protocols.io, where a protocol can be assigned its own identifier (DOI) such that it can be cited independently in the future. Additionally, PLOS ONE offers an option to publish peer-reviewed clinical study protocols. Read more information on sharing protocols at https://plos.org/protocols?utm_medium=editorial-email&utm_source=authorletters&utm_campaign=protocols

# Author comment: Author response to Decision Letter 1

**DOI:** 10.1371/journal.ppat.1014754.r003

29 Sep 2026

Attachment Submitted filename: Reviewers Comments.docx

# Editor report: Decision Letter 1

**DOI:** 10.1371/journal.ppat.1014754.r004

**Authors:** Tracey Lamb, Jacob Tennessen

3 Oct 2026

Dear Dr. Lawniczak,

We are pleased to inform that your manuscript, "High quality chromosomal genome assemblies of three human Plasmodium species directly from natural infections", has been editorially accepted for publication at PLOS Pathogens.

Before your manuscript can be formally accepted and sent to production, you will need to complete our formatting changes, which you will receive by email within a week. Please note that your manuscript will not be scheduled for publication until you have made the required changes.

IMPORTANT NOTES

(1) Copyediting and Proofreading: The corresponding author will receive a typeset proof for review, to ensure errors have not been introduced during production. Please review the PDF proof of your manuscript carefully, as this is the last chance to correct any errors. Please note that major changes, or those which affect the scientific understanding of the work, will likely cause delays to the publication date of your manuscript.

(2) Appropriate Figure Files: Please remove all name and figure # text from your figure files. Please also take this time to check that your figures are of high resolution, which will improve the readbility of your figures and help expedite your manuscript's publication. Please note that figures must have been originally created at 300dpi or higher. Do not manually increase the resolution of your files. For instructions on how to properly obtain high quality images, please review our Figure Guidelines, with examples at: http://journals.plos.org/plospathogens/s/figures.

We encourage you to use PACE (http://pace.apexcovantage.com/), a figure-checking tool which helps authors ensure all figures meet PLOS requirements so that figures can publish in the highest quality. PACE is a digital diagnostic and conversion tool for figure files. It will provide information about any failed check(s) and, if able, will automatically convert the figure into an acceptable file that passes quality checks.

To use PACE, you must first register as a user. Then, login and navigate to the UPLOAD tab, where you will find detailed instructions on how to use the tool.

(3) Striking Image: Please upload a striking still image to accompany your article if one is available (you can include a new image or an existing one from within your manuscript). Should your paper be accepted, this image will be considered for our monthly issue image and may also appear on our website to feature your article. Please upload this as a separate file, selecting "striking image" as the file type upon upload. Please also include a separate "Other" file with a caption, including credits and any potential copyright information. Please do not include the caption in the main article file. If your image is from someone other than yourself, please ensure that the artist has read and agreed to the terms and conditions of the Creative Commons Attribution License at http://journals.plos.org/plospathogens/s/content-license. Please note that PLOS cannot publish copyrighted images.

(4) Press Release or Related Media: If you or your institution will be preparing press materials for this manuscript, please inform our press team in advance at plospathogens@plos.org as soon as possible. We ask that you contact us within one week to plan ahead of our fast Production schedule. If you need to know your paper's publication date for related media purposes, you must coordinate with our press team, and your manuscript will remain under a strict press embargo until the publication date and time.

(5) Update your Profile Information: Now that your manuscript has been provisionally accepted, please log into Editorial Manager and update your profile, if needed. Go to http://www.editorialmanager.com/ppathogens, log in, and click on the "Update My Information" link at the top of the page. Please update your user information to ensure an efficient production and billing process.

(6) LaTeX users only: Our staff will ask you to upload a TEX file in addition to the PDF before the paper can be sent to typesetting, so please carefully review our Latex Guidelines http://journals.plos.org/plospathogens/s/latex in the meantime.

Thank you again for supporting Open Access publishing; we are looking forward to publishing your work in PLOS Pathogens.

Best regards,

Jacob A Tennessen

Guest Editor

PLOS Pathogens

Tracey Lamb

Section Editor

PLOS Pathogens

Dominique Soldati-Favre

Editor-in-Chief

PLOS Pathogens

***********************************************************

# Editor report: Acceptance letter

**DOI:** 10.1371/journal.ppat.1014754.r005

**Authors:** Tracey Lamb, Jacob Tennessen

Dear Dr. Lawniczak,

We are delighted to inform you that your manuscript, "High quality chromosomal genome assemblies of three human Plasmodium species directly from natural infections," has been formally accepted for publication in PLOS Pathogens.

We have now passed your article onto the PLOS Production Department who will complete the rest of the pre-publication process. All authors will receive a confirmation email upon publication.

Thank you again for supporting open-access publishing; we are looking forward to publishing your work in PLOS Pathogens.

Best regards,

Dominique Soldati-Favre

Editor-in-Chief

PLOS Pathogens
